# Early catarrhine evolution in the light of semicircular canal and vestibule shape

**DOI:** 10.1073/pnas.2535242123

**Published:** 2026-09-24

**Authors:** Alessandro Urciuoli, Florian Bouchet, Clément Zanolli, Sergio Almécija, Júlia Arias-Martorell, Oriol Monclús-Gonzalo, Salvador Moyà-Solà, David M. Alba

**Affiliations:** ^a^ Department of Paleontology, University of Zurich, Karl-Schmid-Strasse 4, Zurich 8006, Switzerland; ^b^ Institut Català de Paleontologia Miquel Crusafont (ICP-CERCA), Universitat Autònoma de Barcelona, Edifici ICTA-ICP, c/ Columnes s/n, Cerdanyola del Vallès, Barcelona 08193, Spain; ^c^Division of Palaeoanthropology, Senckenberg Research Institute and Natural History Museum Frankfurt, Senckenberganlage 25, Frankfurt am Main 60325, Germany; ^d^ Universidad de Alcalá, Cátedra de Otoacústica Evolutiva y Paleoantropología (HM Hospitales-UAH), Departamento de Ciencias de la Vida, Alcalá de Henares, Madrid 28871, Spain; ^e^ Univ. Bordeaux, CNRS, MCC, PACEA, UMR 5199, Pessac F-33600, France; ^f^ Institució Catalana de Recerca i Estudis Avançats, Pg. Lluís Companys 23, Barcelona 08010, Spain; ^g^ Division of Anthropology, American Museum of Natural History, Central Park West at 79th Street, New York, NY 10024; ^h^ McDonald Institute for Archaeological Research, University of Cambridge, Cambridge CB2 3ER, United Kingdom

**Keywords:** fossil primates, Cercopithecoidea, Hominoidea, inner ear, Pliopithecoidea

## Abstract

The phylogenetic relationships of early catarrhines (Old World monkeys and apes) are contentious, particularly regarding pliopithecoids, which are considered a clade of Miocene stem catarrhines more derived than most of their Oligocene counterparts. However, their debated affinities and the uncertain phylogenetic relationships of the Oligocene catarrhine *Saadanius* leave their systematic position unresolved. Notably, *Saadanius* displays a mosaic morphology, coupling primitive craniodental traits with a fully ossified ectotympanic (as opposed to the annular/incompletely ossified ear tube of stem catarrhines and early stem hominoids), a characteristic customarily considered as a shared-derived trait of crown catarrhines. To clarify the early evolutionary history of catarrhines, we analyzed the semicircular canal and vestibule morphology of *Saadanius*, the pliopithecoid *Pliobates*, the stem cercopithecoid *Victoriapithecus*, and the stem hominoid *Ekembo*. Using diffeomorphic deformation-based geometric morphometrics on a broad anthropoid sample, we quantified shape variation and inferred ancestral morphotypes. Our results reveal that *Saadanius* retains a primitive, platyrrhine-like inner ear, lacking all crown catarrhine synapomorphies. We also recover similarities between *Pliobates* and the other analyzed pliopithecoid (*Epipliopithecus*), as these taxa share some derived traits with crown catarrhine (particularly, in the anterior semicircular canal), while missing most hominoid and cercopithecoid synapomorphies. Furthermore, *Victoriapithecus* displays a derived, cercopithecoid-like morphology indistinguishable from extant Old World monkeys, whereas *Ekembo* exhibits unexpected derived, ape-like features. These findings support that pliopithecoids are stem catarrhines more derived than *Saadanius* and imply that its tubular ectotympanic must have evolved independently from crown catarrhines, thus representing a remarkable instance of homoplasy.

## Significance

Analysis of inner ear semicircular canal shape across Oligocene and Miocene primates provides insight into long-standing controversies about Old Word anthropoid (catarrhine) evolution. Using advanced 3D geometric morphometrics, we demonstrate that the Oligocene taxon *Saadanius* possesses a highly primitive vestibular apparatus, placing it phylogenetically more basal than more derived stem catarrhines like pliopithecoids. This result strongly implies that a derived, fully ossified tubular ectotympanic evolved independently in *Saadanius* and crown catarrhines. Furthermore, we reconstruct the most reliable morphology for the crown catarrhine last common ancestor, demonstrating marked conservatism in the cercopithecoid lineage versus complex, homoplastic morphological evolution in stem hominoids. These findings provide a definitive anatomical framework for calibrating the mode and tempo of catarrhine primate evolution.

**Fig. 1.**
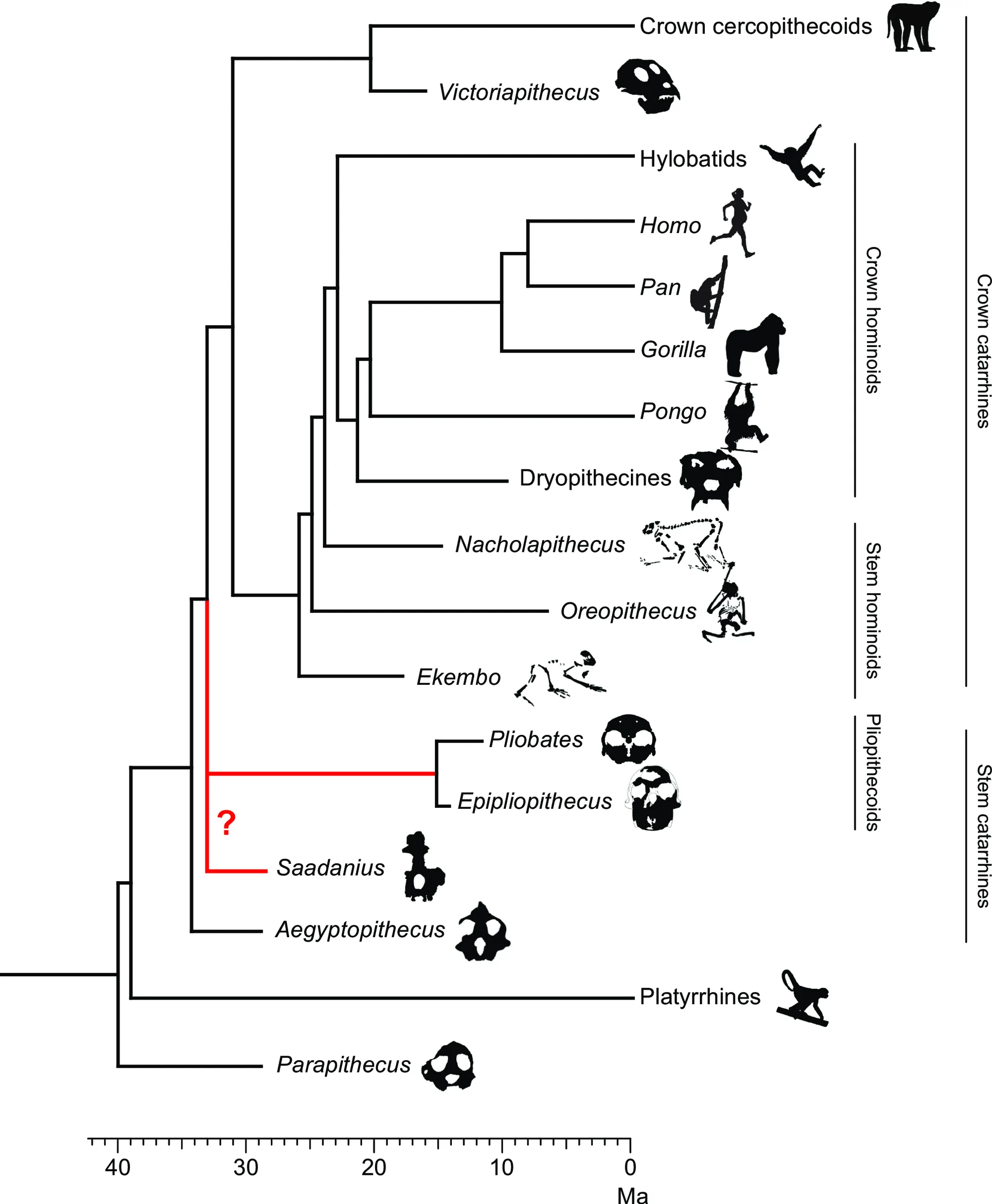
Phylogenetic tree of anthropoid primates showing major clades and fossil taxa analyzed in the present study. The red branches of the trichotomy and question mark highlight the uncertainties at early stages of catarrhine evolution. Silhouettes of extant and extinct primates are not to scale. Branch lengths are based on a molecular tree for anthropoid primates (*SI Appendix*, Fig. S6).

The phylogenetic relationships of Miocene catarrhines are still a matter of contention, including not only unambiguous hominoids (see review in ref. 1) but also small-bodied catarrhines, whose hominoid status is debated (Fig. 1). This is best illustrated by *Kapi* from the Middle Miocene (~13.0 to 12.5 Ma) of India—interpreted as a hylobatid (2, 3) or, alternatively, as a pliopithecoid (4)—and *Pliobates* from the Middle/Late Miocene (~11.6 Ma) (5, 6) of Spain, originally described as a stem hominoid (7) and subsequently reinterpreted as a pliopithecoid (8, 9). Similarly, *Saadanius* from the Late Oligocene of Saudi Arabia (29.0 to 28.0 Ma) (10) has been interpreted as a putative stem catarrhine more derived than both propliopithecoids and pliopithecoids (10, 11). However, some analyses (7, 8, 12) recovered *Saadanius* as more basal than pliopithecoids, and some others (2, 13) even as stem cercopithecoid more basal than *Victoriapithecus* (15 Ma) (14, 15). Although missing data play a role in controversies about the phylogenetic position of some early catarrhine species (e.g., *Kapi*, known from a single molar), they also apply to others known on the basis of more complete material, such as *Saadanius* (a cranium) and *Pliobates* (a partial skeleton with cranium). Hence, the contrasting phylogenetic results obtained for these taxa by different authors largely stem from the contradictory phylogenetic signal provided by different anatomical regions (i.e., the contradictory phylogenetic placement supported by different regions), as illustrated by *Pliobates* in the case of craniodental vs. postcranial evidence (8).

*Saadanius* is exclusively known based on a partial cranium, which displays morphological similarities to propliopithecoids such as *Aegyptopithecus*, from the early Oligocene of Egypt (~30 Ma) (16). Nevertheless, unlike the latter, *Saadanius* shows a completely ossified and tubular ectotympanic (10), coupled with a larger body mass (15 to 20 kg) (10) than *Aegyptopithecus* (7 to 15 kg) (17). Pliopithecoids, from the Early to Late Miocene of Eurasia, display a wide body mass range (3 to 20 kg) (18) and, based on the European *Pliobates* and *Epipliopithecus*, show a partial ossification of the ectotympanic (7, 19), intermediate between earlier stem catarrhines other than *Saadanius* and crown catarrhines. Some authors have regarded an ossified and tubular acoustic meatus as a crown catarrhine synapomorphy, with pliopithecoids representing an intermediate morphological step (20, 21). Nevertheless, the presence of a small and almost completely ossified ectotympanic (approaching the morphology of extant catarrhines) in both the stem cercopithecoid *Victoriapithecus* and the stem hominoid *Ekembo* does not enable conclusively discounting some degree of independent acquisition of this trait in cercopithecoids and hominoids (and maybe *Saadanius*) (7, 18). In turn, *Epipliopithecus* retains multiple cranial and postcranial catarrhine symplesiomorphies, such as the partially ossified ectotympanic (7, 18, 19, 22), while showing superficial similarities in cranial morphology and body proportions with hylobatids (19, 23), as well as some postcranial resemblances to crown hominoids (24). *Pliobates* also shows a partially ossified ectotympanic, yet its postcranium displays more similarities with crown hominoids (7). However, both *Epipliopithecus* (24) and *Pliobates* (8, 9) are currently included in the Pliopithecoidea, with postcranial similarities with hominoids being attributed to convergent evolution (8).

The incongruent signal provided by different anatomical areas, due to frequent homoplasy in catarrhine evolution, is a source of major disagreements regarding the phylogenetic relationships among extinct and extant taxa (1, 25, 26). The bony labyrinth, found in the inner ear surrounding the vestibular system, embeds strong phylogenetic signal and has been proven useful to discriminate major anthropoid clades (27–31). Given the sensory function of the vestibular system, its bony encasing (including the semicircular canals and the vestibule) has been frequently used to make locomotor inferences (32–36). However, other analyses have shown instead that the bony labyrinth morphology is phylogenetically informative (with several synapomorphies identified for crown catarrhines, crown hominoids, and crown hominids) and, hence, useful for assessing the relationships among extinct and extant primates (27–31, 37–40). Based on the analysis of semicircular canal and vestibule variation in extant and extinct anthropoids, *Aegyptopithecus* has been found to retain the plesiomorphic condition for anthropoids, similar to that of the late Oligocene stem anthropoid *Parapithecus* and most extant platyrrhines (27), in agreement with the former’s status as one of the basal-most stem catarrhines. In turn, *Epipliopithecus* shows some synapomorphies of crown catarrhines, while still retaining anthropoid symplesiomorphies, in agreement with a stem catarrhine status more derived than *Aegyptopithecus* (27). However, the condition of early crown catarrhines, such as the stem cercopithecoid *Victoriapithecus*, the stem hominoid *Ekembo,* and the stem catarrhines *Pliobates* and *Saadanius,* are currently unknown. The two latter taxa are key to more reliably reconstructing the ancestral crown catarrhine condition without relying exclusively on extant taxa, which would in turn enable more accurately testing the systematic position of pliopithecoids relative to other stem catarrhines such as *Saadanius*.

With these aims in mind, here we describe the semicircular canal and vestibule shape of the stem catarrhines *Saadanius* and *Pliobates*, as well as the early crown catarrhines *Ekembo* and *Victoriapithecus*. We then use a diffeomorphic deformations approach (DDA) (41, 42) to compare these taxa with a wide sample of extant and other extinct anthropoid primates (the stem catarrhines *Aegyptopithecus* and *Epipliopithecus*, and the putative stem hominoids *Nacholapithecus* and *Oreopithecus*) using a phylogenetically informed shape analysis method. The inclusion of the early crown catarrhines *Ekembo* and *Victoriapithecus* allows for a more reliable reconstruction of the crown catarrhine ancestral morphotype than based exclusively on extant or more derived extinct hominoids and cercopithecoids. We therefore aim to more reliably reconstruct the semicircular canal and vestibule morphotype of the crown catarrhine last common ancestor (LCA) to specifically test whether pliopithecoids constitute a clade instead of a paraphyletic assemblage, as well as to reevaluate their systematic position relative to *Saadanius*.

## Results

### Shape Analysis.

**Fig. 2.**
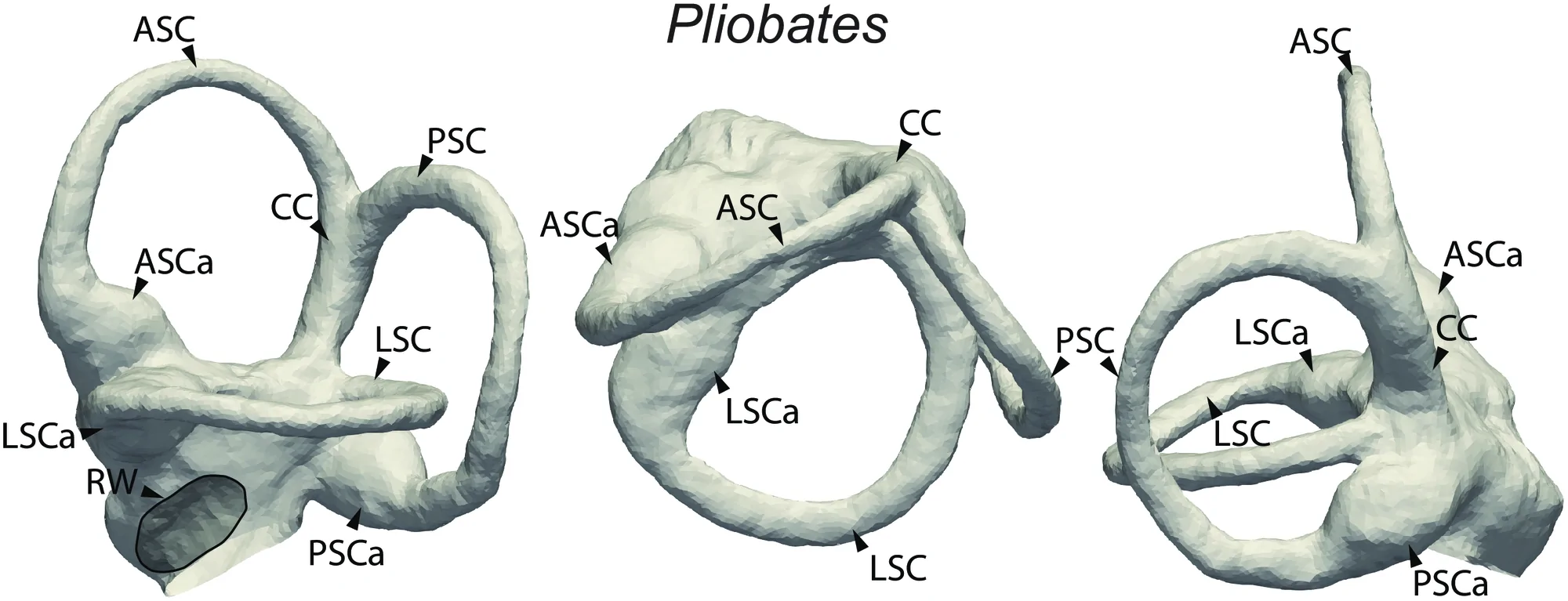
Rendering of the semicircular canal and vestibule 3D model of *Pliobates cataloniae* (IPS58443). Arrows and shaded area highlight the main features used for anatomical descriptions in the present study. Abbreviations: ASC = anterior semicircular canal; ASCa = ampulla of the anterior semicircular canal; CC = common crus; LSC = lateral semicircular canal; LSCa = ampulla of the lateral semicircular canal; PSC = posterior semicircular canal; PSCa = ampulla of the posterior semicircular canal; RW = round window.

**Fig. 3.**
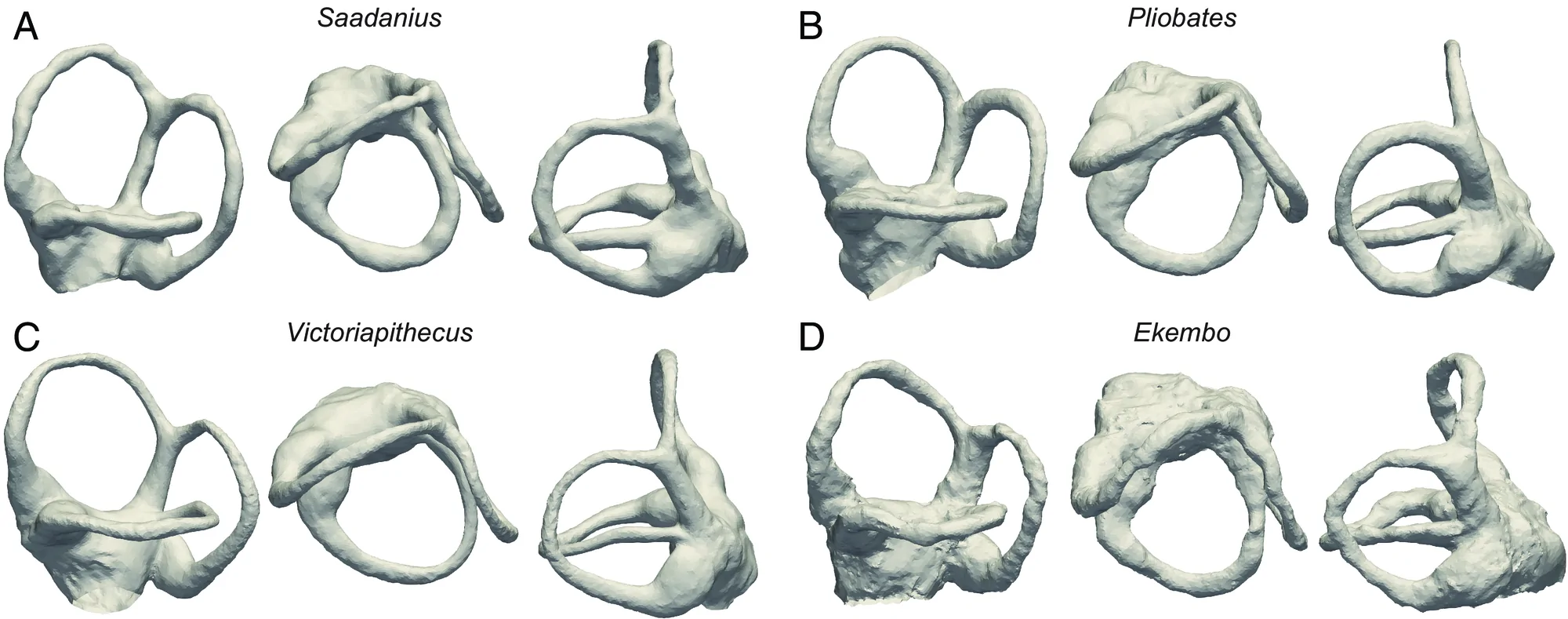
Rendering of the semicircular canal and vestibule 3D models of selected extinct anthropoid primates, from left to right in posterolateral, superior, and posteromedial views (not to scale); (*A*) *Saadanius hijazensis* (SGS-UM 2009-002); (*B*) *Pliobates cataloniae* (IPS58443); (*C*) *Victoriapithecus macinnesi* (KNM-BM 29100); (*D*) *Ekembo heseloni* (KNM-RU 2036al).

**Fig. 4.**
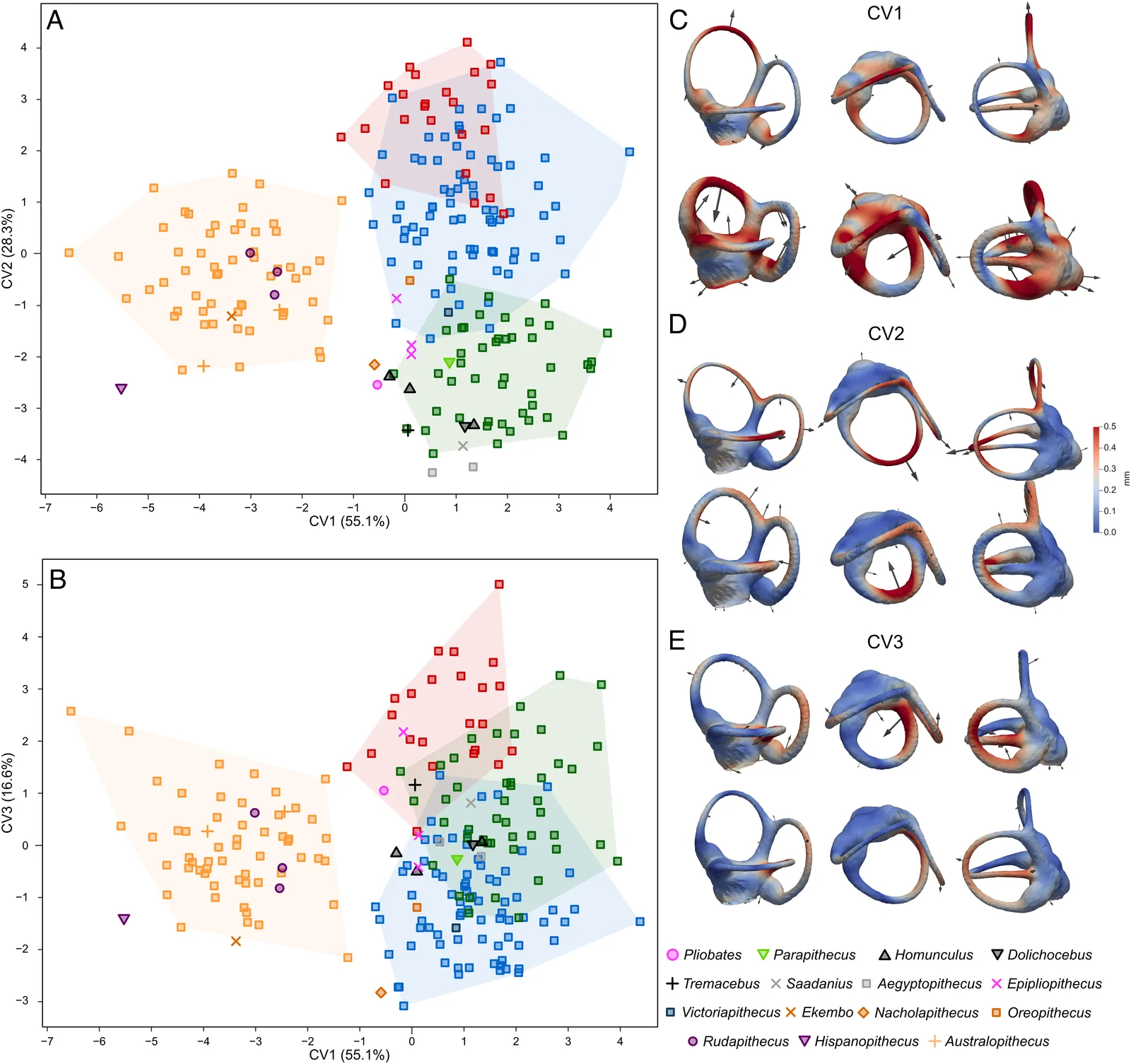
Patterns of vestibular shape variation among anthropoids as captured by a canonical variate analysis (CVA) performed on the first seven principal components of a principal component analysis computed on the raw shape data derived from diffeomorphic deformation analysis. Bivariate plots: (*A*) CV2 vs. CV1; (*B*) CV3 vs. CV1. Variance for each CV is given within parentheses. To the right, renderings of 3D surfaces, in posterolateral (*Left*), superior (*Middle*), and posteromedial (*Right*) views, corresponding to the maximum (*Top*) and minimum (*Bottom*) conformations for: (*C*) CV1; (*D*) CV2; (*E*) CV3. Cumulative deformations from the sample mean are rendered as pseudocolors, with warmer and colder colors corresponding to maximum and minimum displacement, respectively. Symbols are colored based on taxonomy as follows: stem anthropoids = light green; stem platyrrhines = black; stem catarrhines = gray; stem cercopithecoids = navy blue; crown cercopithecoids = dark turquoise blue; stem hominoids = dark orange; stem hominids = purple; crown platyrrhines = dark green; crown hylobatids = red; crown hominids = light orange. Convex hulls in for major extant anthropoid clades are colored in semitransparency as follows: crown platyrrhines = dark green; crown cercopithecoids = dark turquoise blue; crown hylobatids = red; crown hominids = light orange.

The descriptions of the SCs and vestibule of the studied early catarrhine taxa (Figs. 2 and 3 and *SI Appendix*, Figs. S3 and S4) are provided in *SI Appendix, Supplementary Text* 1. A CVA with four extant catarrhine clades distinguished a priori (platyrrhines, cercopithecoids, hylobatids, and hominids), based on the PCA results (*SI Appendix*, Fig. S1), reveals good discrimination among them (Fig. 4 *A* and *B* and Dataset S1), with 96.5% correctly classified individuals after cross-validation. Platyrrhines, cercopithecoids, and hominids only minimally overlap with one another, while hylobatids and cercopithecoids are superimposed to a greater extent. The first canonical variate (CV1, 55.1% of variance) captures differences in the shape of all three semicircular canals (Fig. 4*C*) and separates hominids (on negative scores) from other anthropoids. Negative CV1 scores correlate with thick and small canals (relative to the extent of the vestibule); vertically compressed anterior (ASC) and posterior (PSC) semicircular canals, which are also smaller than the lateral semicircular canal (LSC); laterally compressed LSC (owing to a more laterally positioned slender portion); non-coplanar ASC and LSC (the latter with a more superiorly positioned ampullary portion); and short common crus (CC). The opposite condition is found in the positive space of CV1. *Pliobates* (Fig. 3*B*) and *Epipliopithecus* (*SI Appendix*, Fig. S3*F*) fall on slightly negative scores within the range of hylobatids (*SI Appendix*, Fig. S4*F*) and cercopithecoids (*SI Appendix*, Fig. S4 *C*–*E*), and close to that of platyrrhines (*SI Appendix*, Fig. S4 *A* and *B*), due to its quite slender SCs, moderately long CC, and somewhat vertically elongate ASC and, especially, PSC. *Saadanius* (Fig. 3*A*) shows even greater platyrrhine affinities in the vertically elongate ASC and PSC, long CC, and flat LSC, showing closest similarities with the Oligocene stem catarrhine *Aegyptopithecus* (*SI Appendix*, Fig. S3*E*). The stem cercopithecoid *Victoriapithecus* (Fig. 3*C*) displays clear affinities with extant cercopithecoids in the possession of large, equally sized, and round SCs, a moderately long CC, and a flat LSC that intersects the plane of the PSC. The putative stem hominoids *Nacholapithecus* and *Oreopithecus* (*SI Appendix*, Fig. S3 *G* and *H*) fall on moderately positive scores, whereas the stem hominoid *Ekembo* (Fig. 3*D*) shows a much more hominid-like morphology on this axis (particularly in the vertically compressed ASC and short CC), displaying positive scores outside the range of nonhominid anthropoids. The remaining analyzed fossil taxa fall on more negative scores, within the range of extant platyrrhines, which seem to retain the plesiomorphic anthropoid condition.

The CV2 (28.3% of variance) clearly discriminates between hylobatids and platyrrhines (on positive and negative scores, respectively), whereas hominids and, more extensively, cercopithecoids overlap with both groups. Hence, platyrrhines are distinguished from hylobatids by the presence of moderately thick canals and similarly sized canal radii, long CC, vertically elongate ASC and PSC, and mediolaterally compressed LSC. Conversely, hylobatids possess an anterosuperiorly projecting anterior portion of the ASC and a much larger LSC radius relative to both the ASC and PSC (Fig. 4*D*). *Saadanius* (Fig. 3*A*) shows markedly negative scores due to the possession of vertically elongate ASC and PSC, and a very long CC, most closely resembling the condition of *Aegyptopithecus* (*SI Appendix*, Fig. S3*E*), which shows the most negative scores in the sample. *Pliobates* (Fig. 3*B*) and, to a lesser extent, *Epipliopithecus* (*SI Appendix*, Fig. S3*F*) fall on negative scores along CV2, mainly as consequence of their vertically elongate PSC and round ASC, as well as the similarly sized semicircular canal radii. The latter two features, albeit coupled with a round PSC, are also found in *Nacholapithecus* (*SI Appendix*, Fig. S3*H*), which also falls on negative scores. Both *Ekembo* (Fig. 3*D*) and *Oreopithecus* (*SI Appendix*, Fig. S3*G*) display intermediate scores, owing to their vertically compressed ASC coupled with a round PSC, and a short CC. The intermediate score of *Oreopithecus* (*SI Appendix*, Fig. S3*G*) is also the result of the contrasting signal caused by its anterosuperiorly projecting (hylobatid-like) ASC and small (platyrrhine-like) LSC. Last, *Victoriapithecus* (Fig. 3*C*) possesses moderately negative scores due to its round and similar-sized SCs.

The differences captured by CV3 (18.7% of variance; Fig. 4 *B* and *E*) are more subtle, and the four analyzed groups extensively overlap. Only hylobatids and cercopithecoids can be clearly distinguished from one another on opposite ends of the axis (positive and mostly negative, respectively). This is mainly due to the presence in hylobatids (more variably so in hominids and platyrrhines) of a clear separation between the LSC and the PSC plane, whereas in most cercopithecoids the LSC broadly protrudes inside the PSC trajectory (Fig. 4*E*). Cercopithecoids are further distinctive in the possession of a laterally elongate PSC, a condition also found in some hominids (such as *Gorilla*), *Saadanius*, and *Pliobates* (Fig. 3 *A* and *B*). Together with other stem catarrhines, *Saadanius* and *Pliobates* fall on intermediate CV3 scores, in a portion of the morphospace where all the considered groups overlap. This is due to their possession of a LSC that is somewhat separated from the PSC plane, as well as a round PSC. However, one of the *Epipliopithecus* individuals (*SI Appendix*, Fig. S3*F*) shows a broader separation between the LSC and PSC, and thus falls well within the range of hylobatids and outside that of cercopithecoids. *Nacholapithecus* and *Oreopithecus* (*SI Appendix*, Fig. S3 *G* and *H*) show the opposite pattern, particularly regarding the relative position of the LSC and PSC, but also more laterally projecting PSC, thus falling within the range of cercopithecoids only. In turn, *Ekembo* and *Victoriapithecus* (Fig. 3 *C* and *D*) fall on moderate negative scores as a consequence of their less separate LSC and PSC (especially in the latter).

The posterior and typicality probabilities based on the CVA scores, which respectively indicate the closest phenetic affinities and the potential outlier status of extinct taxa, are reported in *SI Appendix*, Table S1. They confirm that *Saadanius* and *Pliobates* (Fig. 3 *A* and *B*) lack the derived condition of crown catarrhines, as they are classified as platyrrhines with 100% and 98% posterior probabilities, respectively, similarly to the stem catarrhines *Parapithecus* (*SI Appendix*, Fig. S3*A*) and *Aegyptopithecus* (*SI Appendix*, Fig. S3*E*). This is due to the retention of all (*Saadanius*; Fig. 3*F*) or some (*Pliobates*; Fig. 3*B*) anthropoid symplesiomorphies, which cause them to be outliers relative to all the considered crown catarrhines clades, as confirmed by typicality probabilities (*SI Appendix*, Table S1). A similar result is obtained for *Epi-pliopithecus*, as both individuals are classified as platyrrhines with the highest posterior probability and considered outliers relative to all crown catarrhine clades (*SI Appendix*, Table S1)—even though one individual (*SI Appendix*, Fig. S3*F*) is also recovered as an outlier relative to platyrrhines (*SI Appendix*, Table S1). The stem hominoids included in the analysis also show a mixed signal. In particular, *Ekembo* (Fig. 3*D*) is classified as a hominid based on posterior and typicality probabilities (representing an outlier for all other clades), whereas both *Nacholapithecus* and *Oreopithecus* (*SI Appendix*, Fig. S3 *G* and *H*) are in contrast classified as cercopithecoids with high posterior probabilities, although typicality probabilities for *Nacholapithecus* indicate that it is an outlier for all the considered groups (*SI Appendix*, Table S1). Last, *Victoriapithecus* (Fig. 3*C*) is classified as a cercopithecoid based on posterior and typicality probabilities, although it also fits within the platyrrhine range of variation, only being an outlier relative to hylobatids and hominids (*SI Appendix*, Table S1).

### Phylogenetically Aligned Component Analysis, Evolutionary Model, and Inferred Ancestral States.

**Fig. 5.**
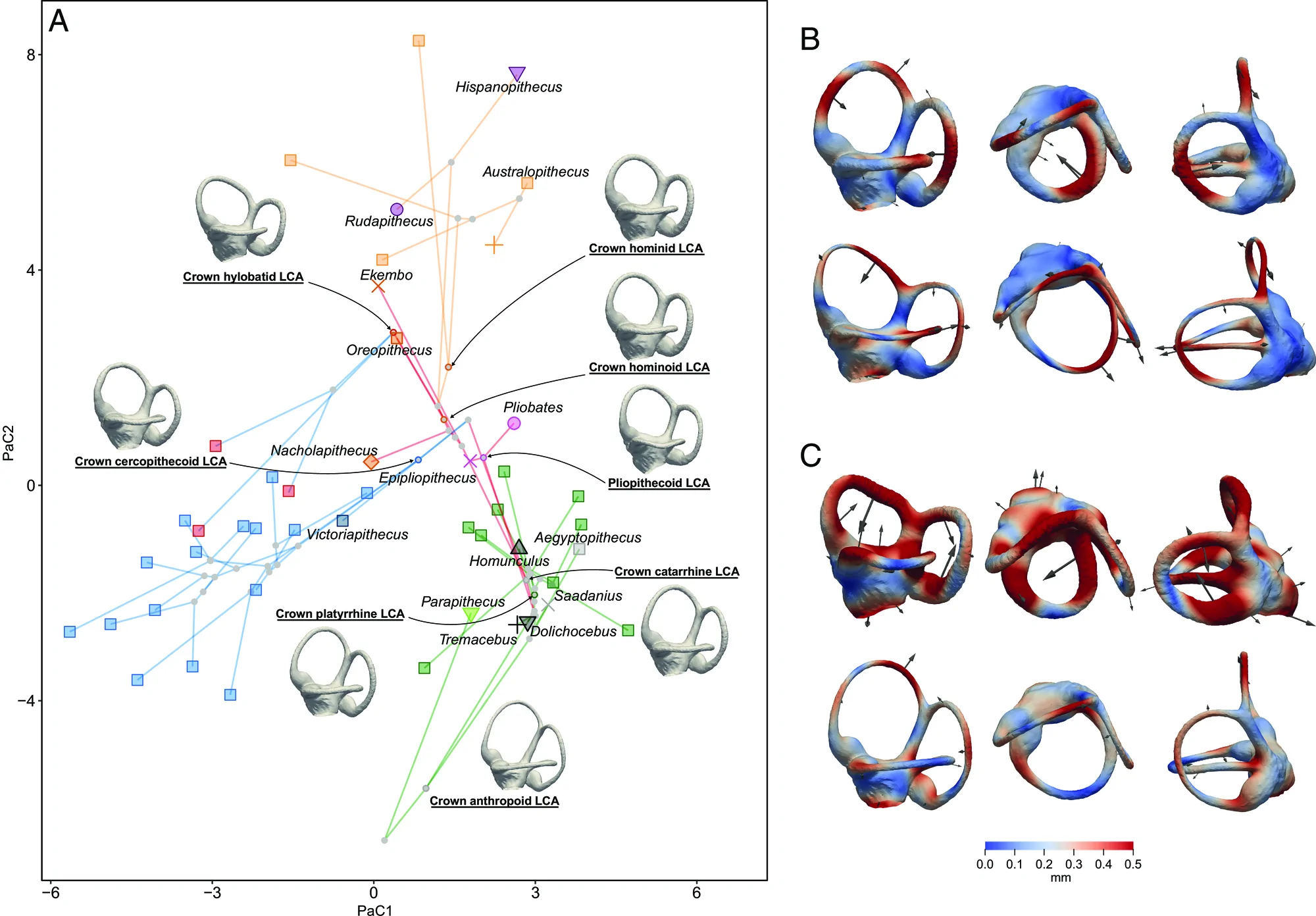
Phylomorphospace based on the results of the phylogenetically aligned component analysis by plotting the phylogenetic tree with *Saadanius* as a stem catarrhine (*SI Appendix*, Fig. S6*A*; see the phylomorphospace plot with the alternative phylogenetic hypothesis in *SI Appendix*, Fig. S5) onto the morphospace and estimating the internal nodes with maximum likelihood based on an Ornstein–Uhlenbeck evolutionary model of evolution with four adaptive optima. (*A*) Bivariate plot of PaC2 vs. PaC1. The 3D models (in posterolateral view) of the estimated LCAs are shown next to the corresponding nodes. To the right, renderings of 3D surfaces, in posterolateral (*Left*), superior (*Middle*), and posteromedial (*Right*) views, corresponding to the maximum (*Top*) and minimum (*Bottom*) conformations for: (*B*) PaC1; (*C*) PaC2. Cumulative deformations from the sample mean are rendered as pseudocolors, with warmer and colder colors corresponding to maximum and minimum displacement, respectively. Symbols are colored based on taxonomy as follows: stem anthropoids = light green; stem platyrrhines = black; stem catarrhines = gray; stem cercopithecoids = navy blue; crown cercopithecoids = dark turquoise blue; stem hominoids = dark orange; stem hominids = purple; crown platyrrhines = dark green; crown hylobatids = red; crown hominids = light orange. Branches are colored based on the assigned adaptive regime: green = base anthropoid; blue = cercopithecoid; orange = hominid; red = stem hominoid.

The morphospace resulting from the phylogenetically aligned component analysis (PaCA), which maximizes the amount of phylogenetic signal captured by each component based on its covariance with shape, is reported in Fig. 5 and *SI Appendix*, Fig. S5 (see Datasets S2 and S3 for interactive 3D plots). This analysis provides a good separation between the considered anthropoid clades, except that hylobatids cluster closely with cercopithecoids, regardless of the phylogenetic hypothesis used for computing the morphospace (i.e., with *Saadanius* included as a stem catarrhine less derived than pliopithecoids or as a stem cercopithecoid less derived than *Victoriapithecus*; *SI Appendix*, Fig. S6). The first two phylogenetically aligned components (PaC1 and PaC2) capture most of the covariance between shape and phylogenetic signal (96.8% of total covariance), thus being the ones reported here. The PaC1 axis (69.3% of covariance) distinguishes well among cercopithecoids plus hylobatids (on negative scores), hominids (intermediate scores), and platyrrhines (positive scores), whereas hominids separate from all other anthropoids along PaC2 (27.5% of covariance).

Based on the observed distribution of the taxa in the morphospace, we tested the likelihood of different evolutionary models—i.e., Brownian motion (BM), early burst (EB), as well as single and multiple regime Ornstein–Uhlenbeck (OU) stabilizing selection model—by means of the Akaike information criterion (AICc) (43), and assessed the most probable evolutionary model for the semicircular canal and vestibule shape. For the two considered phylogenetic hypotheses, the OU model with multiple optima shows the best fit (*SI Appendix*, Table S2), indicating that the shape of the semicircular canals and vestibule has not evolved randomly or constantly, but under changing selection pressures through time. However, the number of optima (and underlying adaptive regimes) differs slightly depending on whether *Saadanius* is considered a stem catarrhine or a stem cercopithecoid. Under the first hypothesis (Fig. 5, *SI Appendix*, Figs. S5 and S6*A*, and Dataset S2), the best supported evolutionary model implies the presence of four optima (i.e., adaptive peaks; *SI Appendix*, Figs. S7*A* and S8 *A*–*D* and Dataset S4). When *Saadanius* is considered a stem cercopithecoid, an evolutionary model with only three adaptive optima is best supported (*SI Appendix*, Figs. S5, S6*B*, S7*B*, S8 *E*–*G* and Datasets S3 and S4). Under both hypotheses, semicircular canal and vestibule shape of *Parapithecus, Aegyptopithecus*, *Saadanius*, and the platyrrhine total group coalesce into an adaptive optimum that resembles the plesiomorphic condition for anthropoids. Such morphology is characterized by a very long CC, as well as vertically elongated ASC and PSC (*SI Appendix*, Fig. S8*A*). A second adaptive optimum is supported for stem hominoids and pliopithecoids, which gravitate toward an intermediate morphology between extant anthropoid major clades (*SI Appendix*, Fig. S8*B*). In turn, *Victoriapithecus* and extant cercopithecoids are attracted to a third adaptive optimum, implying long and slender semicircular canals, with inflated and well-defined ampullae (*SI Appendix*, Fig. S8*C*). Hylobatids appear to have secondarily converged toward the same optimum as cercopithecoids from a more hominid-like ancestral condition. Finally, crown and stem hominids coalesce toward the fourth optimum, characterized by the presence of voluminous yet relatively small semicircular canals and a large vestibule (*SI Appendix*, Fig. S8*D*). In contrast, when *Saadanius* is considered a stem cercopithecoid, no distinct optimum is identified for pliopithecoids and stem hominoids: the former coalesce instead toward the same adaptive optimum as platyrrhines, *Parapithecus,* and *Aegyptopithecus* (*SI Appendix*, Fig. S8*E*), whereas the stem hominoids *Ekembo* and *Oreopithecus* become attracted toward the same optimum as hominids (*SI Appendix*, Fig. S8*F*), and the other stem hominoid (*Nacholapithecus*) secondarily converges toward the cercopithecoid one (*SI Appendix*, Fig. S8*G*). However, it is noteworthy that, when *Saadanius* is considered a stem cercopithecoid, an evolutionary model with the same four optima as under the other hypothesis is only slightly less supported than the three optima model (*SI Appendix*, Table S2), with the only exception that only *Ekembo* and *Oreopithecus* coalesce toward stem hominoids, whereas *Nacholapithecus* still secondarily converges with cercopithecoids (Dataset S4).

**Fig. 6.**
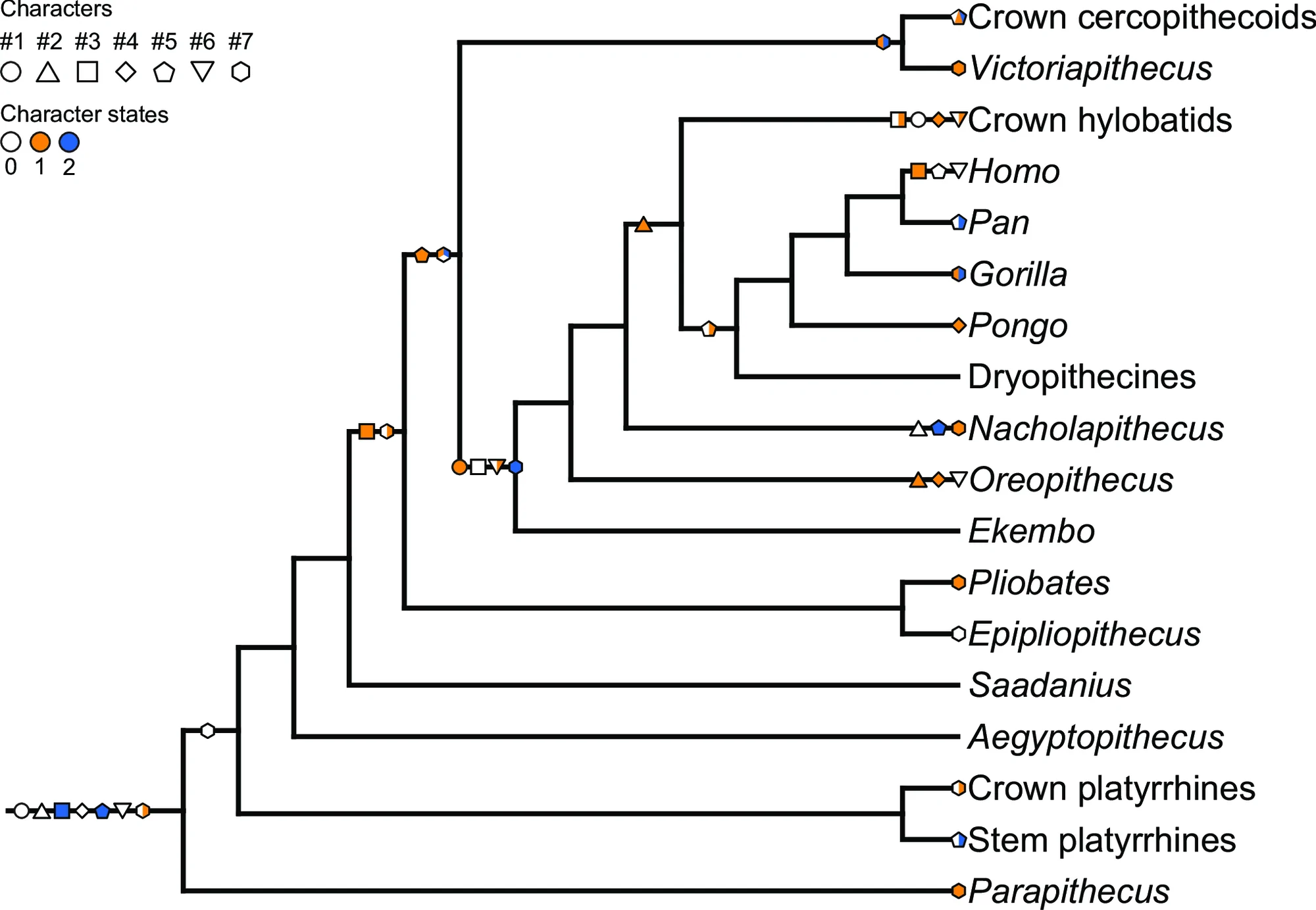
Simplified cladogram of extant anthropoids and selected extinct taxa mapping the morphological characters defined for the semicircular canal and vestibule based on refs. 8 and 27, and reported in *SI Appendix*, Fig. S10 and Table S7. Cladogram topology is based on the stem catarrhine phylogenetic hypothesis for *Saadanius* (8) (see *SI Appendix*, Fig. S11 for the results based on the alternative phylogenetic hypothesis). Character definitions are as follows: #1 Size of the vestibule relative to the SCs: 0 = small; 1 = large; #2 Robusticity of the SCs: 0 = slender; 1 = stout; #3 Shape of the anterior SC: 0 = vertically compressed; 1 = rounded; 2 = elongated superiorly; #4 Shape of the anterior portion of the anterior SC: 0 = nonprojecting anterosuperiorly; 1 = anterosuperiorly projecting; #5 Shape of the posterior SC: 0 = vertically compressed; 1 = rounded; 2 = elongated superiorly; #6 Shape of the lateral SC ampullary portion: 0 = flat or only slightly bent superiorly; 1 = markedly bent superiorly; #7 Length of the CC: 0 = long; 1 = intermediate; 2 = short. Shape and colors correspond to characters and coding.

**Fig. 7.**
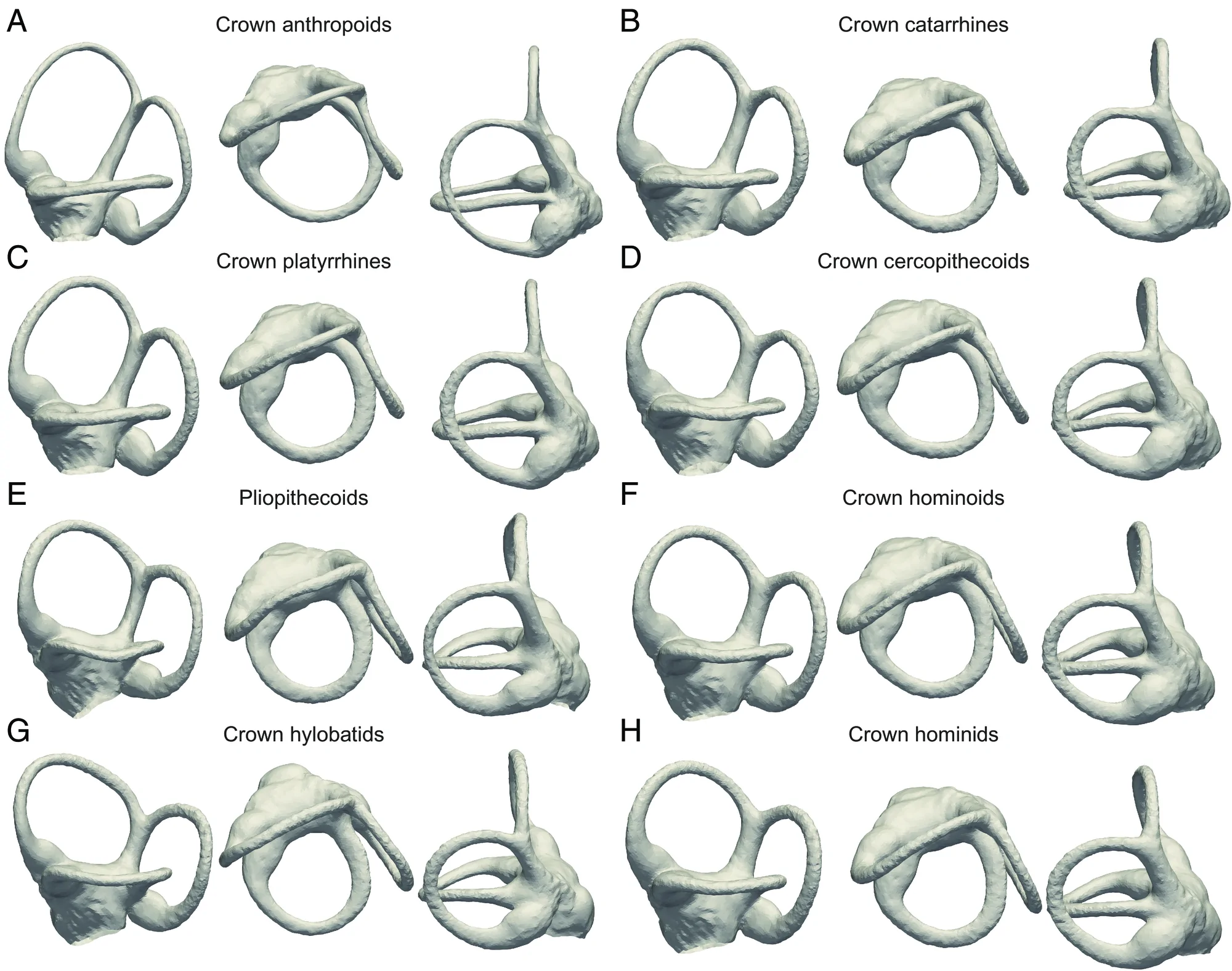
Shape of the last common ancestors (LCAs) of major anthropoid clade estimated using maximum likelihood (assuming an Ornstein–Uhlenbeck evolutionary model of evolution with four adaptive optima) based on the results of the PaCA performed using the phylogenetic tree including *Saadanius* as a stem catarrhine (8) and shown in Fig. 5. The estimated scores of the LCAs are then rotated back into the shape space to visualize the corresponding shape. The 3D models (not to scale) are shown in posterolateral (*Left*), superior (*Middle*), and posteromedial (*Right*) view; (*A*) crown anthropoid LCA; (*B*) crown catarrhine LCA; (*C*) crown platyrrhine LCA; (*D*) crown cercopithecoid LCA; (*E*) pliopithecoid LCA; (*F*) crown hominoid LCA; (*G*) crown hylobatid LCA; (*H*) crown hominid LCA.

When the phylogenetic trees (*SI Appendix*, Fig. S6) are plotted onto the shape space and the ancestral nodes are estimated using maximum likelihood (assuming the best supported evolutionary model for each phylogenetic hypothesis), the resulting phylomorphospaces are fairly similar to one another (Fig. 5, *SI Appendix*, Fig. S5, and Dataset S2 and S3). The differences are identifiable in the reconstructed LCA of a few clades. When *Saadanius* is considered a stem catarrhine (Fig. 5), both the crown anthropoid and crown catarrhine LCA are reconstructed (Figs. 6 and 7 *A* and *B* and *SI Appendix*, Table S2) as slightly more extant anthropoid- and extant catarrhine-like, respectively, than in the alternative phylogenetic hypothesis (*SI Appendix*, Fig. S9 *A* and *B*). Indeed, they show less vertically elongated ASC and PSC, and somewhat shorter and less posteriorly inclined CC. Irrespective of the phylogenetic hypothesis, the crown anthropoid LCA falls in an unoccupied area of the morphospace, whereas the crown catarrhine LCA shows closest similarities with *Saadanius* (Fig. 3*A*) and the stem platyrrhine *Dolichocebus* (*SI Appendix*, Fig. S3*D*). The estimated crown cercopithecoid LCA (Fig. 7*D* and *SI Appendix*, Table S3) appears moderately extant cercopithecoid-like (with closest similarities to *Chlorocebus*, *Nacholapithecus*, *Epipliopithecus*, and to a lesser extent *Victoriapithecus*; Fig. 3*C* and *SI Appendix*, Figs. S3 *F* and *H* and S4*E*) in the round ASC and PSC, and intermediate CC length, while the crown hylobatid LCA is inferred similar to the crown hominid LCA (Fig. 7*G* and *SI Appendix*, Table S3), closest to *Oreopithecus* (*SI Appendix*, Fig. S3*G*). Under the alternative stem cercopithecoid hypothesis, the position of *Saadanius* pulls the crown cercopithecoid LCA toward the anthropoid plesiomorphic optimum, resulting in a more platyrrhine-like morphology, characterized by a long CC and vertically elongated ASC and PSC (*SI Appendix*, Fig. S9*D*), closest to *Callicebus* (*SI Appendix*, Fig. S4*B*) and other platyrrhines. Conversely, the crown hylobatid LCA is reconstructed as great ape-like, showing thick and voluminous canals, as well as a marked vertical ASC compression (*SI Appendix*, Fig. S9*G*), closest to *Rudapithecus* (*SI Appendix*, Fig. S3*J*). Independently from the phylogenetic hypothesis, both crown cercopithecoids and crown hominoids appear more derived (i.e., more distant from the crown catarrhine LCA) than pliopithecoids (Fig. 5). Among cercopithecoids, *Victoriapithecus* (Fig. 3*C*) and cercopithecines (*SI Appendix*, Fig. S4 *C* and *E*) fall closest to the crown cercopithecoid LCA (Fig. 7*D* and *SI Appendix*, Fig. S9*D*). Crown hominids and crown hylobatids are roughly equidistant from the total group and crown hominoid LCAs (Figs. 6*F* and 7 and *SI Appendix*, Table S3). *Oreopithecus* (*SI Appendix*, Fig. S3*G*) falls into an area of the morphospace that is devoid of other taxa but closer to crown hominids and the estimated LCAs of crown hominoids (Fig. 7*G*) and crown hominids (Figs. 5 and 7*H*), as well as close to *Ekembo* (Fig. 3*D*), which in turn shows similarities with *Pan*.

## Discussion

### The Evolution of the Semicircular Canals and Vestibule in Anthropoids.

Our analysis of SC and vestibule shape in a broad sample of extant and extinct anthropoids discriminates well among extant catarrhine clades and, hence, confirms the potential of this anatomical structure for assessing phylogenetic relationships (27–30, 38, 44–48). The inclusion of the early catarrhines *Saadanius*, *Pliobates*, *Victoriapithecus*, and *Ekembo,* along with other stem catarrhines and stem hominoids in the analysis, allows us to revise the polarity of previously coded character states for the semicircular canals and vestibule shape (8, 27, 29, 30). Our results confirm that the SC and vestibule shape of stem and crown platyrrhines is largely symplesiomorphic (27, 29), as illustrated by their similarities with the stem anthropoid *Parapithecus*. Some of these plesiomorphic anthropoid features are also retained by stem catarrhines, which explains their closest phenetic affinities with platyrrhines as retrieved by the CVA (*SI Appendix*, Table S1).

#### Size of the vestibule relative to the semicircular canals.

A large vestibule, with relatively small SC radii, was originally suggested to be either a synapomorphy of the hominoid total group reversed in hylobatids or as a crown hominid synapomorphy independently evolved in *Oreopithecus* (27, 30). The presence in *Ekembo* and *Nacholapithecus* (Fig. 3*D*, Table 1, and *SI Appendix*, Fig. S3*H*) of a large vestibule relative to the SC radii allows us to favor the former interpretation, as further supported by the inferred morphology for the LCA (Fig. 6 and *SI Appendix*, Fig. S10*A* and Table S3). The morphology of hylobatids would thus represent a reversal to the catarrhine (and anthropoid) plesiomorphic condition.

**Table 1.** Character states coded for the studied extinct catarrhines (*Saadanius*, *Pliobates, Victoriapithecus,* and *Ekembo*) and the other fossil comparative species^*^

Species	#1	#2	#3	#4	#5	#6	#7
*Parapithecus grangeri*	0	0	2	0	2	0	1
*Dolichocebus gaimanensis*	0	0	2	0	2	0	0
*Homunculus patagonicus*	0	0	2	0	B	0	0
*Tremacebus harringtoni*	0	0	2	0	2	0	0
*Aegyptopithecus zeuxi*	0	0	2	0	2	0	0
*Saadanius hijazensis*	0	0	2	0	2	0	0
*Epipliopithecus vindobonensis*	0	0	1	0	2	0	0
*Pliobates cataloniae*	0	0	1	0	2	0	1
*Victoriapithecus macinnesi*	0	0	1	0	1	0	1
*Ekembo heseloni*	1	0	0	0	1	1	2
*Oreopithecus bambolii*	1	1	0	1	1	0	2
*Nacholapithecus kerioi*	1	0	0	0	2	1	1
*Hispanopithecus laietanus*	1	1	0	0	1	1	2
*Rudapithecus hungaricus*	1	1	0	0	0	1	2
*Australopithecus africanus*	1	1	0	0	1	1	2

^*^Character definitions (following ref. 27) are as follows: #1 Size of the vestibule relative to the SCs: 0 = small; 1 = large; #2 Robusticity of the SCs: 0 = slender; 1 = stout; #3 Shape of the anterior SC: 0 = vertically compressed; 1 = rounded; 2 = elongated superiorly; #4 Shape of the anterior portion of the anterior SC: 0 = nonprojecting anterosuperiorly; 1 = anterosuperiorly projecting; #5 Shape of the posterior SC: 0 = vertically compressed; 1 = rounded; 2 = elongated superiorly; #6 Shape of the lateral SC ampullary portion: 0 = flat or only slightly bent superiorly; 1 = markedly bent superiorly; #7 Length of the CC: 0 = long; 1 = intermediate; 2 = short. B = (1,2). Characters for a few taxa were previously used in refs. 8 and 27.

#### Robusticity of the semicircular canals.

The paucity of available early hominoid temporal bones did not allow previous studies to clarify whether the possession of robust and thick SCs (characterized by a large canal lumen) in *Oreopithecus* is homologous or homoplastic relative to hominids (27, 29, 30). Slender SCs are displayed by the stem hominoids *Ekembo* and *Nacholapithecus* (Fig. 3*D*, Table 1, and *SI Appendix*, Fig. S3*H*), as well as the reconstructed crown hominoid LCA (Fig. 6 and *SI Appendix*, Figs. S9*G* and S10*B* and Table S3). In turn, a qualitative inspection (as the 3D model is not available) of the SC morphology of *Nyanzapithecus alesi* (see ref. 13) shows the presence of fairly slender SCs, albeit somewhat thicker than in *Ekembo* (Fig. 3*D* and Table 1) and, particularly, *Nacholapithecus* (Table 1 and *SI Appendix*, Fig. S3*H*). Based on this evidence, the hominid-like condition of *Oreopithecus* (*SI Appendix*, Fig. S3*G*) is most likely the result of independent evolution, being possibly related to size-scaling effects (46).

#### Shape of the anterior semicircular canal.

The possession of a superiorly elongate ASC in both stem platyrrhines (such as *Tremacebus*; *SI Appendix*, Fig. S3*B*) and in the Oligocene stem catarrhine *Aegyptopithecus* (*SI Appendix*, Fig. S3*E*) supports the interpretation that this character state is symplesiomorphic for anthropoids (27). This feature is also found in *Saadanius* (Fig. 3*A*), which would either represent a plesiomorphic retention (if *Saadanius* is included as stem catarrhine) or a reversal (if *Saadanius* is considered a stem cercopithecoid) toward the anthropoid plesiomorphic state. Both *Pliobates* and *Epipliopithecus* (Fig. 3*B* and *SI Appendix*, Fig. S3*F*), as well as the stem cercopithecoid *Victoriapithecus* (Fig. 3*C*), have a round ASC, which is thus interpreted as a catarrhine synapomorphy uniting pliopithecoids and crown catarrhines. Crown hominoids are further derived in the possession of a vertically compressed ASC, which is also displayed by the stem hominoids *Ekembo, Nacholapithecus, Oreopithecus* (Fig. 3*D* and *SI Appendix*, Fig. S3 *G* and *H*), and, possibly, *N. alesi* (based on the images published in ref. 13). Hence, a vertically compressed ASC would not represent a synapomorphy of crown hominoids+*Oreopithecus* (*SI Appendix*, Fig. S3*G*; as previously suggested in ref. 27) but for the hominoid total group (Fig. 6 and *SI Appendix*, Fig. S10*C* and Table S3)—at least, including all the aforementioned stem hominoids.

#### Shape of the anterior portion of the anterior semicircular canal.

An anterosuperiorly projecting anterior portion of the ASC is characteristic of hylobatids (Table 1 and *SI Appendix*, Figs. S4*E* and S10*D*), *Pongo* (*SI Appendix*, Fig. S4*F*), and *Oreopithecus* (Table 1 and *SI Appendix*, Fig. S3*G*), but not found in any other anthropoid examined so far. The lack of this feature in *Ekembo*, *Nacholapithecus* (Fig. 3*D*, Table 1, and *SI Appendix*, Fig. S3*H*), and apparently *Nyanzapithecus* (14) may be interpreted as a convergence of *Oreopithecus*, hylobatids, and *Pongo* (*SI Appendix*, Figs. S3*G* and S4 *E* and *F*) (27, 29), or else as a synapomorphy of crown hominoids independently acquired only by *Oreopithecus* and subsequently reversed in hominines (African apes and humans) and dryopithecines (*SI Appendix*, Fig. S3 *I* and *J*) (30). The latter interpretation might be supported by the presence of an anterosuperiorly projecting anterior portion of the ASC in the extinct pongine *Lufengpithecus* (48) (which could not be included in the analyses) suggesting that this character might be characteristic of pongines as a whole rather than only extant orangutans.

#### Shape of the posterior semicircular canal.

The shape of the PSC discriminates crown catarrhines from other anthropoids, as the former are distinguished by either a round or a vertically compressed (instead of vertically elongate) PSC (27). Our results indicate that *Pliobates*, like *Epipliopithecus*, *Aegyptopithecus*, and platyrrhines (Fig. 3*B* and *SI Appendix*, Figs. S3 *E* and *F* and S4 *A* and *B*), retains the plesiomorphic condition for anthropoids (i.e., a vertically elongate PSC). The possession of a round PSC in the stem cercopithecoid *Victoriapithecus* and most stem hominoids (*Ekembo, Oreopithecus*, and, possibly, *N. alesi*; Fig. 3*D*, Table 1, and *SI Appendix*, Fig. S3 *F*–*H*) (13) suggests that this feature may be a synapomorphy of crown catarrhines, in accordance with the inferred morphology of the crown catarrhine LCA (Fig. 6 and *SI Appendix*, Figs. S9*E* and S10*E* and Table S3). However, *Nacholapithecus* (Table 1 and *SI Appendix*, Fig. S3*H*; as well as some *Pan* individuals) display the plesiomorphic condition for this feature, suggesting a reversal. This would also apply to *Saadanius* (Fig. 3*A*) if considered a stem cercopithecoid, thus hinting at a high evolvability for this feature.

#### Shape of the lateral semicircular canal ampullary portion.

The presence of a superiorly bent ampullary portion of the LSC was interpreted as a crown hominoid synapomorphy (27), despite some intraspecific variation (some *Symphalangus syndactylus* individuals show a flat LSC; *SI Appendix*, Fig. S4*H*). Our results indicate that a superiorly bent ampullary portion of the LSC is further displayed by *Ekembo* and *Nacholapithecus* (Fig. 3*D*, Table 1, and *SI Appendix*, Fig. S3*H*), and probably also *Nyanzapithecus* (13). This supports the interpretation that this feature is a synapomorphy of the hominoid total instead of crown group (Fig. 6 and *SI Appendix*, Fig. S10*F*), with the flat LSC of *Oreopithecus* (Table 1 and *SI Appendix*, Fig. S3*G*) likely being a reversal to the plesiomorphic catarrhine condition (27, 46). *Pliobates* (Fig. 3*B* and Table 1), like the older stem catarrhine *Aegyptopithecus* and *Saadanius* (Fig. 3*A*, Table 1, and *SI Appendix*, Fig. S3*E*), retains the plesiomorphic condition (flat LSC) for catarrhines (and anthropoids). In turn, *Epipliopithecus* (Table 1 and *SI Appendix*, Fig. S3*F*) displays an incipient bending of the LSC ampullary portion, albeit flatter than extant and extinct hominoids except *Oreopithecus* (Table 1 and *SI Appendix*, Fig. S3*G*), as well as the inferred hominoid total group LCA (Fig. 6 and *SI Appendix*, Fig. S10*F*).

#### Length of the common crus.

The possession of a long CC was proposed to be an anthropoid symplesiomorphy retained by platyrrhines and the stem catarrhines *Aegyptopithecus* and *Epipliopithecus* (Table 1 and *SI Appendix*, Fig. S3 *E* and *F*), with crown catarrhines being derived in the possession of a moderately long (cercopithecoids, *Nacholapithecus*, and *Pan*) to short (crown hominoids other than *Pan*) CC (27, 30). The moderately long CC of *Pliobates* and *Victoriapithecus* (Fig. 3 *B* and *C* and Table 1), and the short CC of *Ekembo* (Fig. 3*D* and Table 1), *Oreopithecus* (Table 1 and *SI Appendix*, Fig. S3*G*), and *Nyanzapithecus* (13), are in accordance with the previously highlighted pattern for anthropoids (27). The long CC of *Saadanius* (Fig. 3*A* and Table 1) is also in accordance with a stem catarrhine placement for this taxon, whereas if *Saadanius* is considered a stem cercopithecoid this would imply a reversal for this feature to the anthropoid plesiomorphic state. Another minor discrepancy is the difference in CC length between *Epipliopithecus* (long; Table 1 and *SI Appendix*, Fig. S3*F*) and *Pliobates* (moderately long and most closely resembling that of the inferred crown catarrhine LCA; Fig. 3*B*, Table 1, and *SI Appendix*, Fig. S10*G* and Table S3), thus indicating some degree of variation among pliopithecoids for this feature.

### Phylogenetic Implications for Early Catarrhines.

The results of our analysis are in agreement with a stem catarrhine position of *Saadanius* (Fig. 3*A*), more basal than that of other analyzed early catarrhines (*Pliobates*, *Victoriapithecus*, and *Ekembo*; Fig. 3 *B*–*D*). This is supported by its completely plesiomorphic anthropoid morphology of the semicircular canals and vestibule (i.e., the vertically elongated ASC and PSC, as well as the long CC), which most closely resembles that of the older propliopithecoid *Aegyptopithecus* and, to a lesser extent, of the stem anthropoid *Parapithecus* (*SI Appendix*, Fig. S3 *A* and *E*). The evaluation of the best fit evolutionary model under different phylogenetic hypotheses for *Saadanius* (stem catarrhine vs. stem cercopithecoid) further indicates that considering *Saadanius* as a stem cercopithecoid preceding *Victoriapithecus* is less likely than a stem catarrhine [coherently with its nonbilophodont post-canine dentition (10)], as it would entail several reversals to the anthropoid plesiomorphic condition for various aspects of semicircular canal and vestibule shape (ASC and PSC shape, as well as CC length), as well as longer branches between crown catarrhines and, especially, crown cercopithecoids relative to their respective LCAs. It is also noteworthy that, in the best supported evolutionary model for both hypotheses, *Saadanius* is attracted to the platyrrhine instead of the cercopithecoid adaptive optimum. This is at odds with the original interpretation (10), generally followed by subsequent authors (10, 11, 21), that *Saadanius* is more derived than both propliopithecoids and pliopithecoids, given the possession of a fully ossified and tubular ectotympanic more derived than that of the latter, as well as with the stem cercopithecoid status supported by some (2, 11) but not all (7, 8, 12) cladistic analyses. Our results therefore support the view that the tubular ectotympanic became independently ossified in *Saadanius* and crown catarrhines, as previously suggested by some (7, 18, 49), maybe as a result of its large body size (50).

Regarding pliopithecoids, our analysis does not identify any clear synapomorphy of the group in the inner ear. Instead, their vestibular morphology appears quite variable, which is not surprising given that cladistic analyses do not support a particularly close relationship between *Epipliopithecus* (a pliopithecid) and *Pliobates* (a crouzeliid) within the Pliopithecoidea (8). Both taxa exhibit a mosaic of plesiomorphic (platyrrhine-like) features and more derived (catarrhine-like) traits, even incipiently displaying some hominoid-like features (such as the slightly superiorly bent ampullary portion of the LSC in *Epipliopithecus*). Although a potential link between SC shape and function must be interpreted with utmost caution, the morphological variability of pliopithecoids might reflect their considerable locomotor diversity (51, 52). *Epipliopithecus* is generally reconstructed as a generalized arboreal quadruped with limited climbing and suspensory abilities (51, 53, 54), whereas *Pliobates* displays a more derived (hominoid-like) elbow and wrist joints (7, 55–57) as a result of convergence with crown hominoids (9).

Our results for early crown catarrhines show that the vestibular morphology of the stem cercopithecoid *Victoriapithecus* is remarkably similar to that of extant cercopithecoids despite its Middle Miocene (~15 Ma) age (15). This finding, combined with the observation that extant cercopithecoids are quite conservative in their inner ear morphology (27, 29), suggests that their vestibular configuration has remained relatively stable throughout their evolutionary history, ever since their initial divergence from hominoids and the subsequent radiation of crown cercopithecoids. Concerning apes, the stem hominoid *Ekembo* appears much more derived toward the crown hominoid condition than other stem hominoids (*Nacholapithecus* and *Oreopithecus*), even approaching the hominid condition. The derived inner ear of *Ekembo* agrees with the possession of other modern ape-like features in this taxon (such as its incipiently ape-like humeroradial joint), which contrast with its otherwise primitive postcranium indicative of a pronograde body plan (58–60). The inner ear morphology of *Ekembo* contrasts with the more primitive morphology of *Nacholapithecus*, which retains additional plesiomorphic features in the SC and vestibule morphology relative to crown hominoids. This is surprising given that cladistic analyses have generally recovered *Nacholapithecus* (25) or other afropithecids (13) as more derived stem hominoids than proconsulids such as *Ekembo* (1)—with the former, which displays further postcranial adaptations for powerful vertical climbing (61, 62), being even considered a stem hominid by some authors (63, 64). The lack of inner ear data from other afropithecids complicates the interpretation of the inner ear morphology of *Nacholapithecus* but, overall, it is more consistent with a stem hominoid status. This suggests that several features previously thought to be exclusive to crown hominoids, such as a vertically compressed ASC (27), would be characteristic of the hominoid total group. The derived, almost hominid-like morphology of *Ekembo* is difficult to reconcile with its widely accepted, basal stem hominoid status (25). Unless hylobatids diverged more basally than customarily assumed (see discussion in ref. 1), questioning the stem hominoid status of some early Miocene taxa, the morphology of *Ekembo* suggests that the vestibular system bony labyrinth is more prone to homoplasy than generally accepted. The latter possibility is reinforced by the combination in *Oreopithecus* of a generally plesiomorphic inner ear shape with some convergences with hominids superimposed (26, 29).

Overall, our results confirm the utility of inner ear morphology for reassessing the phylogenetic relationships among catarrhines, revealing divergent evolutionary pathways among the major catarrhine clades and helping to revise the systematic position of extinct taxa such as *Saadanius*. Among crown catarrhines, cercopithecoids appear overall more conservative in vestibular morphology than hominoids (particularly, hominids), which display higher morphological variation in this anatomical area, possibly related to their greater locomotor diversity. Furthermore, the conflicting signals recovered from *Ekembo*, *Nacholapithecus*, and especially *Oreopithecus* suggest that the hominoid inner ear did not evolve in a simple, stepwise fashion and that it is also affected by homoplasy to some extent. Therefore, these features should ideally be incorporated in formal cladistic analyses including at least other craniodental characters and subjected to sound morphofunctional analyses in relation to locomotor behaviors, to ascertain whether the purported convergences between putative stem hominoids and crown hominoids might be explained by locomotor adaptations.

## Materials and Methods

Detailed information about materials and methods employed in this study can be found in *SI Appendix, Supplementary Text* S2.

### Studied and Comparative Samples.

We digitally extracted the bony labyrinth 3D models from four extinct catarrhines (*Saadanius hijazensis*, *Pliobates cataloniae*, *Ekembo heseloni*, and *Victoriapithecus macinnesi*) and compared them with those of 31 extant anthropoid genera (*SI Appendix*, Table S4) and 11 extinct anthropoid species dated between 29.9 and 4 Ma (*SI Appendix*, Table S5).

### Shape Analysis Using Diffeomorphic Deformations.

Shape analysis was performed in Defometrica v. 4.3. (https://gitlab.com/icm-institute/aramislab/deformetrica) (65) using a DDA approach—a deformation-based 3D geometric morphometric method (41, 65). Computations were performed in the DeepFry cluster computer (LOEWE Centre for Translational Biodiversity Genomics, Frankfurt am Main) and shape data were analyzed in Rstudio v.2023.9.0.463 (66) for R v.4.3.1 (67). Patterns of shape variation in the raw shape data generated through DDA (deformation fields) were assessed by means of PCA and canonical variate analysis (CVA) using major anthropoid clades as grouping factor. Posterior and typicality probabilities of group membership were also computed.

### Phylogenetically Informed Analyses and Ancestral State Estimation.

The deformation fields were also analyzed with phylogenetic comparative methods that relied on two composite phylogenetic trees (*SI Appendix*, Fig. S6; see *SI Appendix*, Table S6 for tip ages), with*Saadanius* as a stem catarrhine (8) or as a stem cercopithecoid (2, 13). To visually assess the direction of evolutionary changes in the morphospace, we used phylogenetically aligned component analysis (PaCA) (68)—a variant of PCA that maximizes variation along the directions that capture phylogenetic signal (69). We also visualized the output of PaCA as a phylomorphospace including estimated ancestral nodes (70). To test the best evolutionary model fitting the observed distribution in the morphospace, we compared BM, EB, single OU, and multioptima models (*SI Appendix*, Fig. S8), run iteratively on the two alternative tree topologies. The ancestral shape of relevant nodes of the phylogeny was computed based on the PaCA scores, and their scores were subsequently translated back into deformation vectors for visualization. The most parsimonious polarity of previously defined character states for the bony semicircular and vestibule (*SI Appendix*, Tables S3 and S7) (8, 27) was mapped in Mesquite (71) for Windows onto the fixed topology derived from the composite phylogenetic trees.

## Supplementary Material

Appendix 01 (PDF)

## Data Availability

Datasets S1–S5 are available for download from the Zenodo project in ref. 72. The 3D model of the pliopithecid *Pliobates cataloniae* can be downloaded from MorphoSource ref. 73.

## References

[1] A. Urciuoli, D. M. Alba, Systematics of Miocene apes: State of the art of a neverending controversy. J. Hum. Evol. **175**, 103309 (2023). 10.1016/j.jhevol.2022.103309 36716680

[2] C. C. Gilbert , New middle miocene ape (Primates: Hylobatidae) from Ramnagar, India fills major gaps in the hominoid fossil record. Proc. Biol. Sci. **287**, 20201655 (2020). 10.1098/rspb.2020.1655 PMC7542791 32900315

[3] C. C. Gilbert , Additional analyses of stem catarrhine and hominoid dental morphology support Kapi ramnagarensis as a stem hylobatid. J. Hum. Evol. **199**, 103628 (2025). 10.1016/j.jhevol.2024.103628 39764860

[4] X. Ji , The earliest hylobatid from the Late Miocene of China. J. Hum. Evol. **171**, 103251 (2022). 10.1016/j.jhevol.2022.103251 36113226

[5] D. M. Alba, I. Casanovas-Vilar, M. Garcés, J. M. Robles, Ten years in the dump: An updated review of the Miocene primate-bearing localities from Abocador de Can Mata (NE Iberian Peninsula). J. Hum. Evol. **102**, 12–20 (2017). 10.1016/j.jhevol.2016.09.012 28012461

[6] D. M. Alba , A revised (earliest Vallesian) age for the hominoid-bearing locality of Can Mata 1 based on new magnetostratigraphic and biostratigraphic data from Abocador de Can Mata (Valles-Penedes Basin, NE Iberian Peninsula). J. Hum. Evol. **170**, 103237 (2022). 10.1016/j.jhevol.2022.103237 35988385

[7] D. M. Alba , Miocene small-bodied ape from Eurasia sheds light on hominoid evolution. Science **350**, aab2625 (2015). 10.1126/science.aab2625 26516285

[8] F. Bouchet , The Miocene primate *Pliobates* is a pliopithecoid. Nat. Commun. **15**, 2822 (2024). 10.1038/s41467-024-47034-9 PMC10984959 38561329

[9] F. Bouchet , Molar enamel–dentine junction shape of *Pliobates cataloniae* and other Iberian pliopithecoids. J. Hum. Evol. **195**, 103581 (2024). 10.1016/j.jhevol.2024.103581 39243703

[10] I. S. Zalmout , New Oligocene primate from Saudi Arabia and the divergence of apes and Old World monkeys. Nature **466**, 360–364 (2010). 10.1038/nature09094 20631798

[11] N. Stevens , Palaeontological evidence for an Oligocene divergence between Old World monkeys and apes. Nature **497**, 611–614 (2013). 10.1038/nature12161 23676680

[12] S. F. Al-Ashqar , An Early Miocene ape from the biogeographic crossroads of African and European Hominoidea. Science **391**, 1383–1386 (2026). 10.1126/science.adz4102 41886561

[13] I. Nengo , New infant cranium from the African Miocene sheds light on ape evolution. Nature **548**, 169–174 (2017). 10.1038/nature23456 28796200

[14] B. R. Benefit, *Victoriapithecus*: The key to Old World monkey and catarrhine origins. Evol. Anthropol. **7**, 155–174 (1999).

[15] B. R. Benefit, M. L. McCrossin, Earliest known Old World monkey skull. Nature **388**, 368–371 (1997). 10.1038/41078 9237753

[16] E. R. Seiffert, Revised age estimates for the later Paleogene mammal faunas of Egypt and Oman. Proc. Natl. Acad. Sci. U.S.A. **103**, 5000–5005 (2006). 10.1073/pnas.0600689103 PMC1458784 16549773

[17] J. M. G. Perry , Articular scaling and body mass estimation in platyrrhines and catarrhines: Modern variation and application to fossil anthropoids. J. Hum. Evol. **115**, 20–35 (2018). 10.1016/j.jhevol.2017.10.008 29150186

[18] D. R. Begun, “The Pliopithecoidea” in The Primate Fossil Record, W. C. Hartwig, Ed. (Cambridge University Press, 2002), pp. 221–240.

[19] H. Zapfe, Die Primatenfunde aus der miozänen Spaltenfüllung von Neudorf an der March (Děvínská Nová Ves), Tschechoslowakei. Schweiz. Palaeontol. Abh. **78**, 1–293 (1961).

[20] F. S. Szalay, E. Delson, Evolutionary History of the Primates (Academic Press, 1979).

[21] P. J. Andrews, T. Harrison, E. Delson, R. L. Bernor, L. Martin, “Distribution and biochronology of European and Southwest Asian Miocene catarrhines” in The Evolution of Western Eurasian Neogene Mammalian Faunas, R. L. Bernor, V. Fahlbusch, H.-W. Mittmann, Eds. (Columbia University Press, 1996), pp. 168–207.

[22] T. Harrison, “Catarrhine origins” in A Companion to Paleoanthropology, D. R. Begun, Ed. (Blackwell Publishing Ltd., 2013), pp. 376–396.

[23] E. L. Simons, J. G. Fleagle, “The history of extinct gibbon-like primates” in Gibbon and Siamang Vol. 2. Anatomy, Dentition, Taxonomy, Molecular Evolution and Behavior, D. M. Rumbaugh, Ed. (Karger, 1973), pp. 121–148.

[24] S. Almécija , Early anthropoid femora reveal divergent adaptive trajectories in catarrhine hind-limb evolution. Nat. Commun. **10**, 4778 (2019). 10.1038/s41467-019-12742-0 PMC6838095 31699998

[25] K. D. Pugh, Phylogenetic analysis of Middle-Late Miocene apes. J. Hum. Evol. **165**, 103140 (2022). 10.1016/j.jhevol.2021.103140 35272113

[26] D. M. Alba , Miocene ape evolution: Where does *Oreopithecus* fit in? Boll. Soc. Paleontol. Ital. **63**, 153–182 (2024).

[27] A. Urciuoli , A comparative analysis of the vestibular apparatus in *Epipliopithecus vindobonensis*: Phylogenetic implications. J. Hum. Evol. **151**, 102930 (2021). 10.1016/j.jhevol.2020.102930 33422741

[28] N. Morimoto , Variation of bony labyrinthine morphology in Mio–Plio–Pleistocene and modern anthropoids. Am. J. Phys. Anthropol. **173**, 276–292 (2020). 10.1002/ajpa.24098 32529656

[29] A. Urciuoli , The evolution of the vestibular apparatus in apes and humans. eLife **9**, e51261 (2020). 10.7554/eLife.51261 PMC7054002 32122463

[30] A. Urciuoli , Reassessment of the phylogenetic relationships of the late Miocene apes *Hispanopithecus* and *Rudapithecus* based on vestibular morphology. Proc. Natl. Acad. Sci. U.S.A. **118**, e2015215118 (2021). 10.1073/pnas.2015215118 PMC7865142 33495351

[31] J. del Río , Allometry, function and shape diversification in the inner ear of platyrrhine primates. J. Mamm. Evol. **28**, 135–143 (2021).

[32] F. Spoor, B. Wood, F. Zonneveld, Implications of early hominid labyrinthine morphology for evolution of human bipedal locomotion. Nature **369**, 645–648 (1994). 10.1038/369645a0 8208290

[33] F. Spoor , The primate semicircular canal system and locomotion. Proc. Natl. Acad. Sci. U.S.A. **104**, 10808–10812 (2007). 10.1073/pnas.0704250104 PMC1892787 17576932

[34] A. Walker, T. M. Ryan, M. T. Silcox, E. L. Simons, F. Spoor, The semicircular canal system and locomotion: The case of extinct lemuroids and lorisoids. Evol. Anthropol. **17**, 135–145 (2008).

[35] R. David , Motion from the past: A new method to infer vestibular capacities of extinct species. C. R. Palevol **9**, 397–410 (2010).

[36] R. David, A. Stoessel, A. Berthoz, F. Spoor, D. Bennequin, Assessing morphology and function of the semicircular duct system: Introducing new in-situ visualization and software toolbox. Sci. Rep. **6**, 32772 (2016). 10.1038/srep32772 PMC5015051 27604473

[37] F. Spoor, F. Zonneveld, Comparative review of the human bony labyrinth. Yearb. Phys. Anthropol. **41**, 211–251 (1998). 10.1002/(sici)1096-8644(1998)107:27+<211::aid-ajpa8>3.3.co;2-m 9881527

[38] A. Urciuoli , Semicircular canals shed light on bottleneck events in the evolution of the Neanderthal clade. Nat. Commun. **16**, 972 (2025). 10.1038/s41467-025-56155-8 PMC11842635 39979299

[39] Y. Zhang, A. Urciuoli, C. Zanolli, O. Kullmer, X. J. Wu, Three-dimensional geometric morphometric analysis of the bony labyrinth of Xujiayao 6. J. Hum. Evol. **189**, 103514 (2024). 10.1016/j.jhevol.2024.103514 38547737

[40] C. M. Smith , Divergent otolithic systems in the inner ear of *Paranthropus* *robustus* and *Australopithecus* *africanus*. J. Hum. Evol. **199**, 103624 (2025). 10.1016/j.jhevol.2024.103624 39675267

[41] S. Durrleman , Morphometry of anatomical shape complexes with dense deformations and sparse parameters. Neuroimage **101**, 35–49 (2014). 10.1016/j.neuroimage.2014.06.043 PMC4871626 24973601

[42] A. Beaudet, Investigating the primate fossil record using diffeomorphic deformation: What have we learnt? Bull. Mem. Soc. Anthropol. Paris **37**, 98–104 (2025).

[43] H. Akaike, A new look at the statistical model identification. IEEE Trans. Autom. Control **19**, 716–723 (1974).

[44] R. Quam, C. Lorenzo, I. Martínez, A. Gracia-Téllez, J. L. Arsuaga, The bony labyrinth of the middle Pleistocene Sima de los Huesos hominins (Sierra de Atapuerca, Spain). J. Hum. Evol. **90**, 1–15 (2016). 10.1016/j.jhevol.2015.09.007 26767955

[45] M. Conde-Valverde , The bony labyrinth in the Aroeira 3 Middle Pleistocene cranium. J. Hum. Evol. **124**, 105–116 (2018). 10.1016/j.jhevol.2018.08.003 30201119

[46] A. Urciuoli , New evidence from the inner ear and teeth of the Late Miocene ape *Oreopithecus* based on a partial cranium from Baccinello. J. Mammal. Evol. **33**, 6 (2026).

[47] A. Le Maître, F. Guy, G. Merceron, D. S. Kostopoulos, Morphology of the bony labyrinth supports the affinities of *Paradolichopithecus* with the Papionina. Int. J. Primatol. **44**, 209–236 (2023). 10.1007/s10764-022-00329-4 PMC9931825 36817734

[48] Y. Zhang , *Lufengpithecus* inner ear provides evidence of a common locomotor repertoire ancestral to human bipedalism. Innovation **5**, 100580 (2024). 10.1016/j.xinn.2024.100580 PMC10928440 38476202

[49] D. R. Begun, “Evolution of the Pliopithecoidea” in The International Encyclopedia of Primatology, A. Fuentes, Ed. (John Wiley & Sons, Hoboken, 2017), 10.1002/9781119179313.wbprim0165.

[50] D. R. Begun, “Catarrhine origins and evolution” in A Companion to Biological Anthropology, C. S. Larsen, Ed. (John Wiley & Sons, Hoboken, ed. 2, 2023), pp. 381–399.

[51] J. Arias-Martorell , Morphological affinities of the proximal humerus of *Epipliopithecus* *vindobonensis* and *Pliopithecus* *antiquus*: Suspensory inferences based on a 3D geometric morphometrics approach. J. Hum. Evol. **80**, 83–95 (2015). 10.1016/j.jhevol.2014.08.012 25234205

[52] J. Arias-Martorell , A proximal radius of *Barberapithecus* *huerzeleri* from Castell de Barberà: Implications for locomotor diversity among pliopithecoids. J. Hum. Evol. **157**, 103032 (2021). 10.1016/j.jhevol.2021.103032 34233242

[53] T. R. Rein, T. Harrison, C. P. E. Zollikofer, Skeletal correlates of quadrupedalism and climbing in the anthropoid forelimb: Implications for inferring locomotion in Miocene catarrhines. J. Hum. Evol. **61**, 564–574 (2011). 10.1016/j.jhevol.2011.07.005 21872907

[54] T. R. Rein, K. Harvati, T. Harrison, Inferring the use of forelimb suspensory locomotion by extinct primate species via shape exploration of the ulna. J. Hum. Evol. **78**, 70–79 (2015). 10.1016/j.jhevol.2014.08.010 25234204

[55] G. Raventós-Izard, O. Monclús-Gonzalo, S. Moyà-Solà, D. M. Alba, J. Arias-Martorell, Ulnar morphology of *Pliobates cataloniae* (Pliopithecoidea: Crouzeliidae): Insights into catarrhine locomotor diversity and forelimb evolution. J. Hum. Evol. **202**, 103663 (2025). 10.1016/j.jhevol.2025.103663 40101397

[56] J. Arias-Martorell , Ape-like locomotor adaptations in the radius of the stem catarrhine *Pliobates* shed light on hominoid evolution. iScience **29**, 114622 (2026). 10.1016/j.isci.2025.114622 PMC12857360 41623466

[57] J. Arias-Martorell , The distal humerus of *Pliobates cataloniae* (Primates: Pliopithecoidea): Functional and locomotor inferences. J. Mammal. Evol. **33**, 36 (2026).

[58] C. V. Ward, A. Walker, M. F. Teaford, I. Odhiambo, Partial skeleton of *Proconsul nyanzae* from Mfangano Island, Kenya. Am. J. Phys. Anthropol. **90**, 77–111 (1993). 10.1002/ajpa.1330900106 8470757

[59] C. V. Ward, “Postcranial and locomotor adaptations of hominoids” in Handbook of Paleoanthropology 2nd Edition, W. Henke, I. Tattersall, Eds. (Springer, 2015), pp. 1011–1030.

[60] J. Arias-Martorell, A. Urciuoli, S. Almécija, D. M. Alba, M. Nakatsukasa, The radial head of the Middle Miocene ape *Nacholapithecus kerioi*: Morphometric affinities, locomotor inferences, and implications for the evolution of the hominoid humeroradial joint. J. Hum. Evol. **178**, 103345 (2023). 10.1016/j.jhevol.2023.103345 36933453

[61] M. Nakatsukasa, Y. Kunimatsu, *Nacholapithecus* and its importance for understanding hominoid evolution. Evol. Anthropol. **18**, 103–119 (2009).

[62] M. Pina, M. Nakatsukasa, New quantitative analyses of the *Nacholapithecus kerioi* proximal ulna confirm morphological affinities with *Equatorius* and large papionins. Am. J. Biol. Anthropol. **185**, e25000 (2024). 10.1002/ajpa.25000 39049556

[63] D. M. Alba, Fossil apes from the Vallès-Penedès Basin (NE Iberian Peninsula): Phylogenetic, paleobiogeographic and paleobiological implications. Evol. Anthropol. **21**, 254–269 (2012). 10.1002/evan.21312 23280922

[64] Y. Kunimatsu, M. Nakatsukasa, D. Shimizu, Y. Nakano, H. Ishida, Loss of the subarcuate fossa and the phylogeny of *Nacholapithecus*. J. Hum. Evol. **131**, 22–27 (2019).

[65] A. Bône, M. Louis, B. Martin, S. Durrleman, “Deformetrica 4: An open-source software for statistical shape analysis” in Shape in Medical Imaging. Shape MI 2018, M. Reuter , Eds. (Springer, 2018), pp. 3–13.

[66] RStudio Team, RStudio: Integrated development for R (Version 2023.9.0.463, PBC, Boston, MA 2023). https://posit.co/products/open-source/rstudio/.

[67] R Core Team, R: A language and environment for statistical computing (Version 4.3.1, R Foundation for Statistical Computing, 2023).

[68] C. M. Smith, R. David, S. Almécija, J. T. Laitman, A. S. Hammond, First evolutionary insights into the human otolithic system. Comm. Biol. **7**, 1244 (2024). 10.1038/s42003-024-06966-0 PMC11447226 39358583

[69] M. L. Collyer, D. C. Adams, Phylogenetically aligned component analysis. Methods Ecol. Evol. **12**, 359–372 (2021).

[70] B. Sidlauskas, Continuous and arrested morphological diversification in sister clades of characiform fishes: A phylomorphospace approach. Evolution **62**, 3135–3156 (2008). 10.1111/j.1558-5646.2008.00519.x 18786183

[71] W. P. Maddison, D. R. Maddison, Mesquite: A modular system for evolutionary analysis (Version 4.01, 2025). https://www.mesquiteproject.org.

[72] A. Urciuoli , Supplementary data for the manuscript titled “Early catarrhine evolution in the light of semicircular canal and vestibule shape” ref. PNAS2025-35242. Zenodo. 10.5281/zenodo.21357634. Deposited 14 July 2026. PMC13624677 42784509

[73] A. Urciuoli , Supplementary Online Material (3D Model). MorphoSource. 10.17602/M2/M907650. Deposited 29 August 2026.

