# Supplementary material for: Early catarrhine evolution in the light of semicircular canal and vestibule shape
Source: Proc Natl Acad Sci U S A. 2026 Sep 24;123(39):e2535242123. doi: 10.1073/pnas.2535242123 (PMC13624677; doi:10.1073/pnas.2535242123)
Supplement: Supplementary file 1 [file pnas.2535242123.sapp.pdf]

Supplementary Information

Early catarrhine evolution in the light of semicircular canal and vestibule shape

Alessandro Urciuoli*, Florian Bouchet, Clément Zanolli, Sergio Almécija, Júlia Arias-Martorell, Oriol Monclús-Gonzalo, Salvador Moyà-Solà, David M. Alba.

*To whom correspondence may be addressed: alessandro.urciuoli@pim.uzh.ch

This file includes:

Supplementary Text 1–2

Supplementary Figures S1–12

Supplementary Tables S1-7

Supplementary References

Supplementary Text S1. Description and comparisons of early catarrhines vestibular system bony labyrinth.

Pliobates cataloniae – The three semicircular canals and the vestibule from the left side are well preserved in the *Pliobates* holotype (IPS58443.1)[1], without visible fractures or appreciable diagenetic deformation. The entire bony labyrinth is filled with sediment, which nevertheless shows a good contrast relative to the surrounding fossilized bone and thus allows for the digital extraction of the 3D model (Fig. 3b). The canals are slender as in non-hominid anthropoids (Fig. 3b) and oriented almost orthogonally to one another. The bony vestibule is moderately large relative to canal radius size, albeit less so than in hominids (Supplementary Fig. S4g–h). The anterior semicircular canal (ASC) is planar and round, as in crown catarrhines (Fig. 3c–d; Supplementary Figs. S3g–k, S4c–h) and *Epipliopithecus* (Supplementary Fig. S3f), despite of the presence of a somewhat straight and vertical lateral portion (just above the ASC ampulla). The posterior semicircular canal (PSC) deviates to some extent

from planarity (appearing somewhat twisted in posterolateral view) and is vertically elongate, albeit less so than in most platyrrhines (both extant and fossil; Supplementary Figs. S3b–d, S4a–b) and in older African stem catarrhines (*Aegyptopithecus* and *Saadanius*; Fig. 3a; Supplementary Fig. S3e), rather resembling *Epipliopithecus* (Supplementary Fig. S3f). The lateral semicircular canal (LSC) is flat, as in non-hominoid anthropoids (Fig. 3a–c; Supplementary Figs. S3a–f, S4a–e), and slightly compressed mediolaterally. Its slender portion connects with the ampulla forming a right angle, as in some extant and fossil platyrrhines (Supplementary Figs. S3b–d, S4a–b). The common crus (CC) is moderately long, closer to that of crown catarrhines (Fig. 3c–d; Supplementary Figs. S3g–k, S4c–h) and *Epipliopithecus* (Supplementary Fig. S3f) than platyrrhines (Supplementary Fig. S4a–b) and other stem catarrhines (Fig. 3a; Supplementary Fig. S3e), in which it is longer. At the CC apex, the ASC and PSC connect forming an acute angle, as in several platyrrhines (Supplementary Fig. S4a–b), *Aegyptopithecus* (Supplementary Fig. S3e), and, to a lesser extent, *Saadanius* (Fig. 3a).

<u>*Saadanius hijazensis*</u> – The temporal bone petrous portion of the *Saadanius hijazensis* holotype (the partial cranium SGS-UM 2009-002)[2,3] is completely preserved (although isolated) and does not show neither diagenetic deformations nor major fractures. The three SCs (Fig. 3a) are similarly undistorted and complete, although the canal lumen is completely filled with sediment. Thanks to the good contrast between the fossilized bone and the sediment matrix, the segmentation was performed semiautomatically. The three canals have almost orthogonally orientation to one another. The ASC shows some medially oriented bending of its superiormost portion, whereas both the LSC and the PSC are coplanar and do not present twisting. Relative to the vestibular recesses, the SCs are large and slender, as in non-hominid

anthropoids. The ASC and PSC are superiorly elongated, and the CC is markedly long, as in extinct and extant platyrrhines and in the stem anthropoid *Parapithecus* (Supplementary Fig. S3a) and the stem catarrhine *Aegyptopithecus* (Supplementary Fig. S3e). The LSC is flat (as in non-hominoid primates; Fig. 3a–c; Supplementary Figs. S3a–f, S4a–e) and elongated laterally, resulting in almost triangular shape, unlike most other anthropoids. The LSC is positioned inferiorly relative to the PSC, almost touching the ampullary portion of the latter as in several platyrrhines (Supplementary Fig. S4a–b) and *Aegyptopithecus* (Supplementary Fig. S3e). The angle at the CC apex is acute, yet not too distant from the right angle, as in *Lagothrix* (Supplementary Fig. S4a), but less acute than in most platyrrhines, *Pliobates* (Fig. 3b), and *Aegyptopithecus* (Supplementary Fig. S3e).

Ekembo heseloni – The temporal bone KNM-RU 2036al belonging to the holotype of *Ekembo heseloni* is well preserved (although isolated relative to the cranium), without deformations and/or fractures that may affect the bony labyrinth shape. The lumen of the SCs, vestibule, and cochlea is filled with sediment. Given the relatively poor contrast between the sediment and the fossilized petrosal bone, the segmentation required careful manual corrections. The three SCs and vestibule are, however, intact and were digitally extracted (Fig. 3d). The SCs are slender (as in non-hominid anthropoids; Fig. 3a–c; Supplementary Figs. S3a–f, S4a–e), equally sized, and moderately small relative to the size of the osseous vestibule (as in extant and extinct hominoids besides hylobatids; Supplementary Figs. S3g–k, S4g–h). The PSC and LSC, as well as the ASC and PSC, are perpendicular to one another, while the ASC and LSC form an acute angle. The ASC and the LSC (less so) show a twisting of their path. Particularly, the ASC superiormost portion markedly bends medially, whereas the LSC shows a superiorly bent ampullary portions (as in crown hominoids

and *Nacholapithecus*; Supplementary Figs. S3h–k, S4f–h). The ASC is also vertically compressed (as in crown hominoids and *Oreopithecus*; Supplementary Figs. S3g, i–k, S4f–h), without an anterosuperiorly projecting anterior portion (unlike *Oreopithecus*, orangutans, and hylobatids; Supplementary Figs. S3g, S4f–h). The PSC is flat and its lateralmost portion is somewhat projecting, not dissimilar from the condition found in some *Pan* individuals, while the LSC is round. The CC is quite thick and short (as in crown hominoids and *Oreopithecus*; Supplementary Figs. S3g, i–k, S4f–h). At the CC apex, the ASC and PSC form an obtuse angle, as consequence of the marked bending of the ASC.

<u>*Victoriapithecus macinnesi*</u> – The petrosal bone of the *Victoriapithecus macinnesi* holotype KNM-BM 29100 is well preserved and still attached to the cranium. The bony labyrinth lumen is partially filled with sediments, which enabled a straightforward digital segmentation of the cavity. The SCs (Fig. 3c) are large relative to the vestibule and slender (as in non-hominoid anthropoids; Fig. 3a–b; Supplementary Figs. S3a–f, S4a–e), round (as in crown catarrhines; Fig. 3d; Supplementary Figs. S3g–k, S4c–h), and form right angles between one another. The ASC and LSC are similar in size, whereas the PSC is somewhat smaller. The ASC superiormost portion is somewhat inclined medially, whereas both the PSC and LSC are flat. The CC is moderately long (as in crown catarrhines; Fig. 3d; Supplementary Figs. S3g–k, S4c–h), somewhat shorter than in *Pliobates* (Fig. 3b) but longer than in *Ekembo* (Fig. 3d) and in hominoids (Supplementary Figs. S3i–k, S4f–h). The ASC and PSC form a right angle at the CC apex, similar to that of *Epipliopithecus* (Supplementary Fig. S3f) and most extant cercopithecoids (Supplementary Fig. S4c–e).

Supplementary Text S2. Extended materials and methods descriptions.

Studied and comparative samples

Studied sample We digitally extracted the bony labyrinth 3D models from the following analyzed catarrhine species: *Saadanius hijazensis*, *Pliobates cataloniae*, *Ekembo heseloni*, and *Victoriapithecus macinnesi*. The left temporal bone belonging to the holotype of *S. hijazensis* (the partial cranium SGS-UM 2009-002) is housed at the Paleontology Unit of the Saudi Geological Survey in Jeddah (Saudi Arabia). It was µCT scanned using a OMNI-X HD600 high-resolution computed tomography system at the Center for Quantitative Imaging at Pennsylvania State University, obtaining a pixel size of 64.4 µm and an interslice distance of 69.0 µm[3]. The holotype of *P. cataloniae* (IPS58443) from ACM/C8-A4 is housed at the Institut Català de Paleontologia Miquel Crusafont (Sabadell, Spain). The main cranial fragment (IPS58443.1) was scanned at the Centro Nacional de Investigación sobre la Evolución Humana (Burgos, Spain) using a V|Tome|X s 240 GE high-resolution µCT system, obtaining an isometric voxel size of 21.0 µm. The specimen had previously been scanned using both X-ray[1] and neutron-based µCT[4], yet the detailed morphology of the bony labyrinth was poorly visible due to low contrast of the sediments filling the cavities. Only the left bony labyrinth, albeit filled with sediment, was available for analysis, whereas the right one was excluded due to the presence of extensive fractures. The temporal bone from the holotype of *E. heseloni* (KNM-RUal 2036) and the complete cranium of *V. macinnesi* (KNM-MB 29100) are housed at the National Museums of Kenya (Nairobi) and were µCT scanned in situ by Fred Spoor, Fredrick Kyalo Manthi, and Isaiah Nengo, using a BIR ACTIS 225/300 scanner belonging to the Max Planck Institute for Evolutionary Anthropology (Leipzig, Germany), achieving a voxel size of 63.8 µm and 44.0 µm, respectively. Only the left temporal bone of

Victoriapithecus was available for analysis. All fossils were partly or entirely filled with sediments but showed good contrast between the sediment matrix and the fossilized bone tissue, thus enabling the digital extraction of the 3D models of the semicircular canals and vestibule. The segmentation was performed in Avizo v.8.1 (FEI Visualization Sciences Group, Houston) using multiple gray value thresholds and manual corrections due to the pervasive presence of matrix infill.

<u>Comparative sample</u> The extant comparative sample includes 198 μCT scans of dried and ethanol-preserved crania and temporal bones belonging to 31 anthropoid genera (nine platyrrhines, 15 cercopithecoids, four hominids, and three hylobatids; see Supplementary Table S5 for additional details). We also included 11 extinct anthropoid species (Supplementary Table S6) dated between 29.9 and 4 Ma, thus covering most of anthropoid evolution. Most of the μCT scans and/or bony labyrinth 3D models of the anthropoid comparative sample (Supplementary Tables S5, S6) were downloaded from MorphoSource.org (https://www.morphosource.org) with the following exceptions: *Homo sapiens* individuals were either previously published in ref. 5 or downloaded from Zenodo[6]; some *Gorilla*, *Pan*, and *Pongo* specimens were obtained from the digital repository of the European Synchrotron Radiation Facility (ESFR; https://paleo.esrf.fr/) and originally published in ref. 7.

As for the studied material, all the μCT slice stacks were processed using Avizo. The bony labyrinth was segmented using the semiautomatic 'watershed' Avizo segmentation module, applying additional manual corrections when the canals were (at least partially) filled by sediments. The left bony labyrinth was digitally extracted when available, otherwise that from the right side was extracted and mirrored. The semicircular canals and vestibule were separated from the cochlea following a

previously established protocol[5,8], which performs a straight cut on the 3D mesh using landmarks placed along the maximum curvature of the junction between the vestibule and the cochlea as reference. The obtained 3D meshes were postprocessed in Geomagic Studio v. 2014.3.0 (3D Systems, Rock Hill) to fill the hole generated from the cut and correct possible imperfections in the mesh. Each model was decimated to 30,000 (±100) triangles and then aligned in Avizo using the 'Align Surface' module with the 'rigid + uniform scale' option, which minimizes the distance between the triangular faces composing the surfaces by rotating, translating, and rescaling each object relative to a template.

Diffeomorphic deformation analysis

Shape analysis was performed by means of DDA, a deformation-based 3D geometric morphometric method that considers the geometric correspondence of continuous surfaces, thus computing the magnitude and direction of deformation of the analyzed meshes from a template—i.e., the sample average shape[9–10]. The DDA approach produces similar results to those obtained with standard landmark-based 3DGM methods, while having the advantage of being less prone to interobserver error related to landmark placement, as the alignment and surface matching procedures are automatized. This approach is particularly relevant for structures with a complex 3D morphology (such as the vestibular system), which does not allow clear definition of homologous points, and has been successfully applied to several structures in paleoanthropology[5,8,11 12–15]. To analyze shape, we used the 'estimate' function of the open-source software Deformetrica v. 4.3. (http://www.deformetrica.org/)[10] to compute a deterministic atlas of the deformation vectors, using the 'GradientAscent' optimization method. The computations were performed in the DeepFry cluster

computer (LOEWE Centre for Translational Biodiversity Genomics, Frankfurt am Main). The shape data was imported and analyzed in Rstudio v.2023.9.0.463[16] for R v.4.3.1[17] using the RToolsForDeformetrica v.0.1 package[18].

Shape analysis

Patterns of shape variation in the deformation fields (i.e., the raw shape data generated through DDA) were assessed by means of a canonical variate analysis (CVA), performed using major anthropoid clades (i.e., platyrrhines, cercopithecoids, hylobatids, and hominids) as grouping factor. Due to methodological limitations of the CVA approach when applied to highly-multidimensional data (as in the case of most 3DGM analyses), we first computed in R a PCA of the deformation fields data to reduce the dimensionality (Supplementary Figure S1). The CVA was then performed on the minimum number of PCs that yielded the highest percentage of correctly classified individuals after cross-validation to avoid overestimating among group variance[15]. We thus selected the first seven PCs (70.0% cumulative variance). The CVA was computed using the 'cva' function of the Morpho v.2.7 package for R[19].

To evaluate the morphological affinities of fossil specimens to the a priori defined groups for the CVA, we computed posterior and typicality probabilities using the 'typprobClass' function of the Morpho package. Typicality probabilities, unlike posterior probabilities, do not assume that the tested individuals must belong to one of the groups, so that they do not sum to unity. They instead reflect how 'typical' a given observation is within a specific model or distribution by measuring the probability of observing a Mahalanobis distance as large as, or larger than, the one calculated for the specific individual, assuming it belongs to the group. Hence, a significant

probability (p < 0.05) is interpreted to indicate that the individual is an outlier for the group distribution.

Phylogeny-informed analyses and ancestral state estimation

Phylogenetic trees The deformation fields were also analyzed with phylogenetic comparative methods that relied on two composite phylogenetic trees (Supplementary Fig. S6). The trees were obtained by using a recently published molecular phylogeny based on the whole genome of 233 extant primate species[20], to which extinct species were appended based on published cladistic analyses: one included *Saadanius* as a stem catarrhine more basal than pliopithecoids and more derived than propliopithecoids[21]; the other considered *Saadanius* as a stem cercopithecoid less derived than *Victoriapithecus*[2,7]. The divergence time and branch lengths for *Hoolock* and *Symphalangus* (the latter genus was not included in ref. 20) were taken from a different study that also relied on the whole genome of 52 primate species[22]. The divergence of extinct species was arbitrarily placed 1 Myr before the estimated divergence age of the next derived node or before the first appearance datum of the considered species (when older). Tip ages for extinct primates were based on chronostratigraphic data and are summarized in Supplementary Table S6.

Phylogenetically aligned component analysis of shape data To visually assess the direction of evolutionary changes in the morphospace, we employed a recently developed method termed phylogenetically aligned component analysis (PACA) that has received some attention in anthropological analyses[e.g.,23]. This variant of PCA enables the rearrangement of the eigenvectors to maximize the amount of phylogenetic signal captured by the first components[24], thus enabling us to assess which shape patterns are most correlated with phylogenetic signal. Contrary to the

phylogenetic PCA[25], which allows to remove the effect of phylogeny in shape analyses, the PACA computes the singular value decomposition of the cross-products between the matrix of the residuals and the phylogenetic covariance matrix. Hence, PACA maximizes variation along the directions that capture phylogenetic signal, while ensuring that the Euclidean distances between observations in the morphospace remain unchanged[24]. To assess the evolutionary distances, we visualized the output of PACA as a phylomorphospace based on species shape averages and on a phylogeny to estimate ancestral nodes (see below)[26]. The analysis was carried out in R using the 'gm.prcomp' function of the geomorph v.4.0.8 package for R[27] with enabled 'align.to.phy' option.

Evolutionary modeling and adaptive optima analysis To test the best evolutionary model fitting the observed distribution in the morphospace, we compared BM, EB, single OU, and multi-optima models (Supplementary Fig. S7; Supplementary Data S4). To avoid overparameterization while capturing the most relevant evolutionary variance, the analyses were performed on the first two phylogenetically aligned components (PaC1 and PaC2), which maximize the phylogenetic signal of the morphospace. Due to the contradictory phylogenetic placement of *Saadanius* based on latest cladistic analyses[7,21,28], all macroevolutionary models were run iteratively on the two alternative composite tree topologies. We fitted the evolutionary models to the shape data using the mvMORPH (v.1.2.1)[27] package for R, while we mapped the a priori defined adaptive regimes onto the branches of the phylogenies using the 'paintSubTree' function from the phytools (v.2.5.-2)[29] R package. We used: the 'mvBM' function to fit a single-rate BM, which assumes a random-walk evolution with constant variance; the 'mvEB' function to fit an EB model, which simulates an initial rapid phenotypic divergence followed by a progressive deceleration in evolutionary rates;

we employed the ‘mvOU’ function to fit OU models, which model evolution as a random walk pulled toward one or more adaptive peaks (optima). We tested both a single-optimum model (OU1) for the entire tree and several multiple-optima models (OUM). These included adaptive landscapes with three and four optima (i.e., positively selected adaptive peaks around which species that evolve under a similar adaptive regime gravitate), each one with multiple configurations designed to test whether particular taxa (such as stem hominoids, pliopithecoids, and *Saadanius*) fit with their respective ancestral adaptive regime or had already shifted toward a new optimum (Supplementary Data S4). To determine the best supported evolutionary model, we relied on the small-sample size corrected AICc and Akaike weights, which were computed using the ‘aicw’ function in mvMORPH.

<u>Ancestral node estimation</u> The ancestral shapes of relevant nodes (i.e., those corresponding last common ancestors [LCAs] of major anthropoid clades) of the phylogeny were computed based on the PACA scores estimated by the ‘gm.prcomp’. The LCA PaC1 and PaC2 scores were estimated under the best-fitting OUM model using the ‘estim’ function of mvMORPH with the 'asr = TRUE' method. The ancestral states for the remaining higher-order components (PaC3–PaC45) were estimated under a standard Brownian motion model using maximum likelihood[30] via the 'fastAnc' function in the phytools package. The reasons behind this hybrid reconstruction approach are in the high dimensionality of the score matrix, which would cause the OUM model to trailing components that represent negligible variance results in an ill-conditioned, rank-deficient evolutionary covariance matrix; this causes severe numerical instability and leads to biologically impossible shape extrapolations[31–32]. The matrix of the LCA scores was subsequently translated back into deformation vectors (momenta) to compute the 3D iterative deformations of the sample average

using the 'compute' function of Deformetrica, allowing us to accurately visualize the hypothesized ancestral morphologies. All interactive plots for the phylomorphospaces and alternative adaptive landscape hypotheses were prepared using the plotly v.4.12.0[33] R package.

Estimation of semicircular canal character state polarity The most parsimonious polarity of previously defined character states for the bony semicircular and vestibule (Supplementary Table S3)[8,21] was mapped in Mesquite[34] for Windows onto the fixed topology derived from the composite phylogenetic trees.

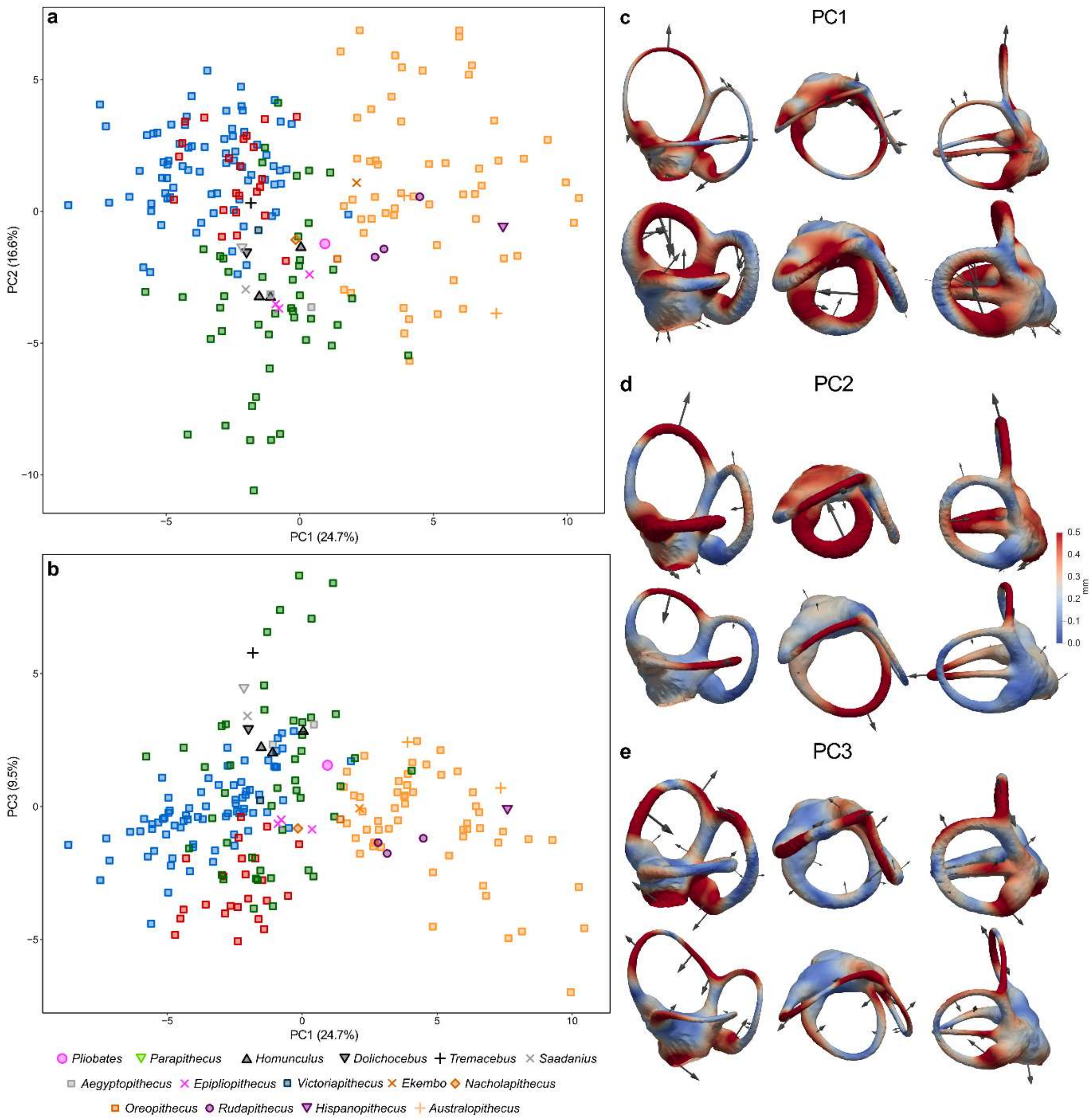

Supplementary Figure S1. a–b) Results of a principal component analysis performed on the deformation fields (the raw shape data) derived from the diffeomorphic deformation analysis, as shown by bivariate plots of PC2 (a) and PC3 (b) relative to PC1. Variance captured by each axis is given within parentheses. Symbols and colors are based on taxonomy as in Fig. 4. c–e) Renderings of 3D surfaces, in posterolateral (left), superior (middle), and posteromedial (right) views, corresponding to the maximum (top) and minimum (bottom) conformations for PC1 (c), PC2 (d), and PC3 (e). Cumulative deformations from the sample mean are rendered as pseudocolors

with warmer and colder colors corresponding to maximum and minimum displacement, respectively.

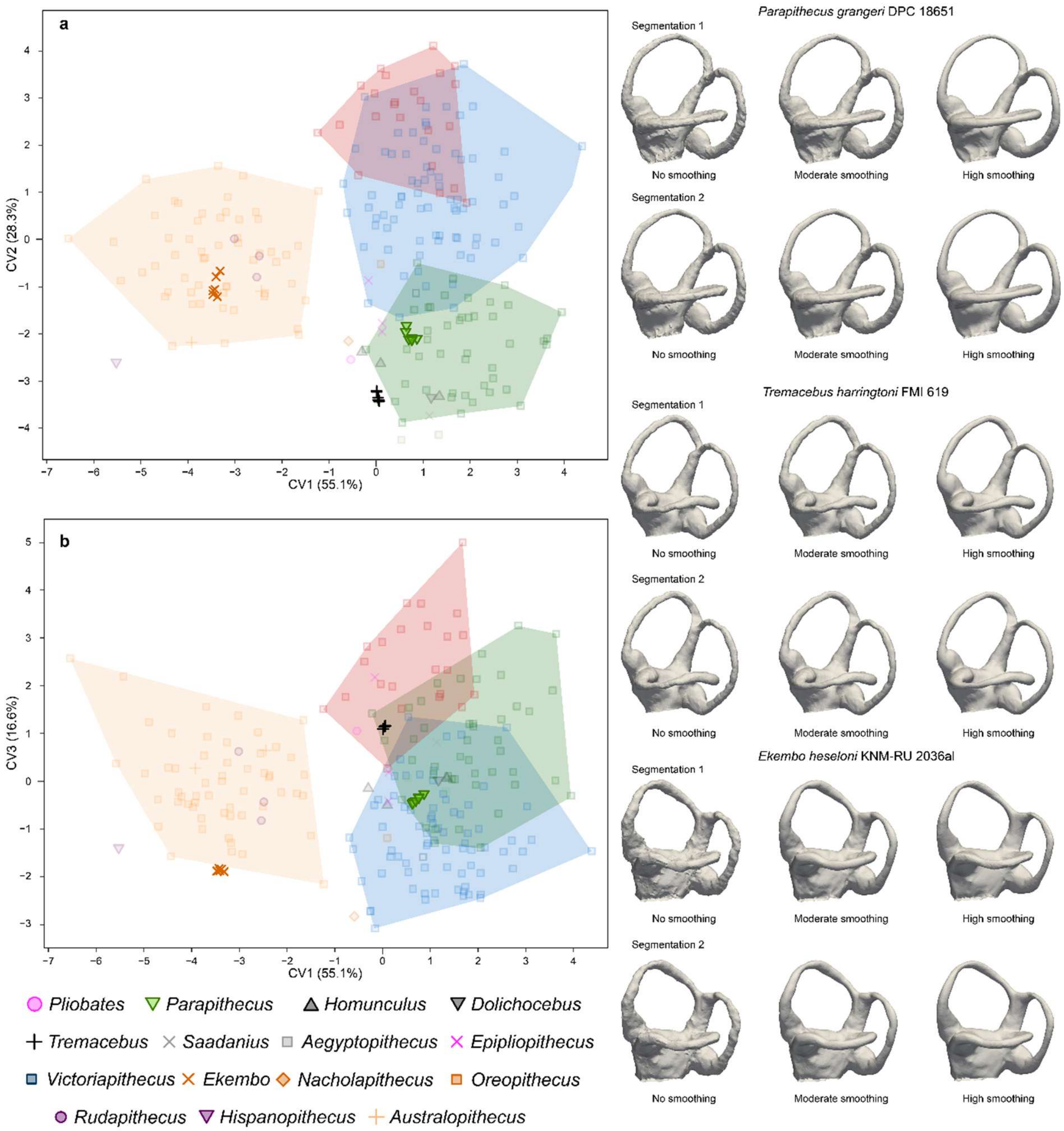

Supplementary Figure S2. Sensitivity analysis for the diffeomorphic deformation analysis (DDA) showing the effect of manual segmentation and smoothing. Bivariate plots of a canonical variate analysis (CVA) performed on the first seven principal components of a principal component analysis (Supplementary Fig. S1) computed on the raw shape data derived from diffeomorphic deformation analysis highlight the tight scatter of the points corresponding to multiple segmentations and different levels of smoothing performed for *Parapithecus, Tremacebus*, and *Ekembo.* a) CV2 vs. CV1;

b) CV3 vs. CV1. Variance for each CV is given within parentheses. Symbols are color coded as in Supplementary Fig. S1.

v

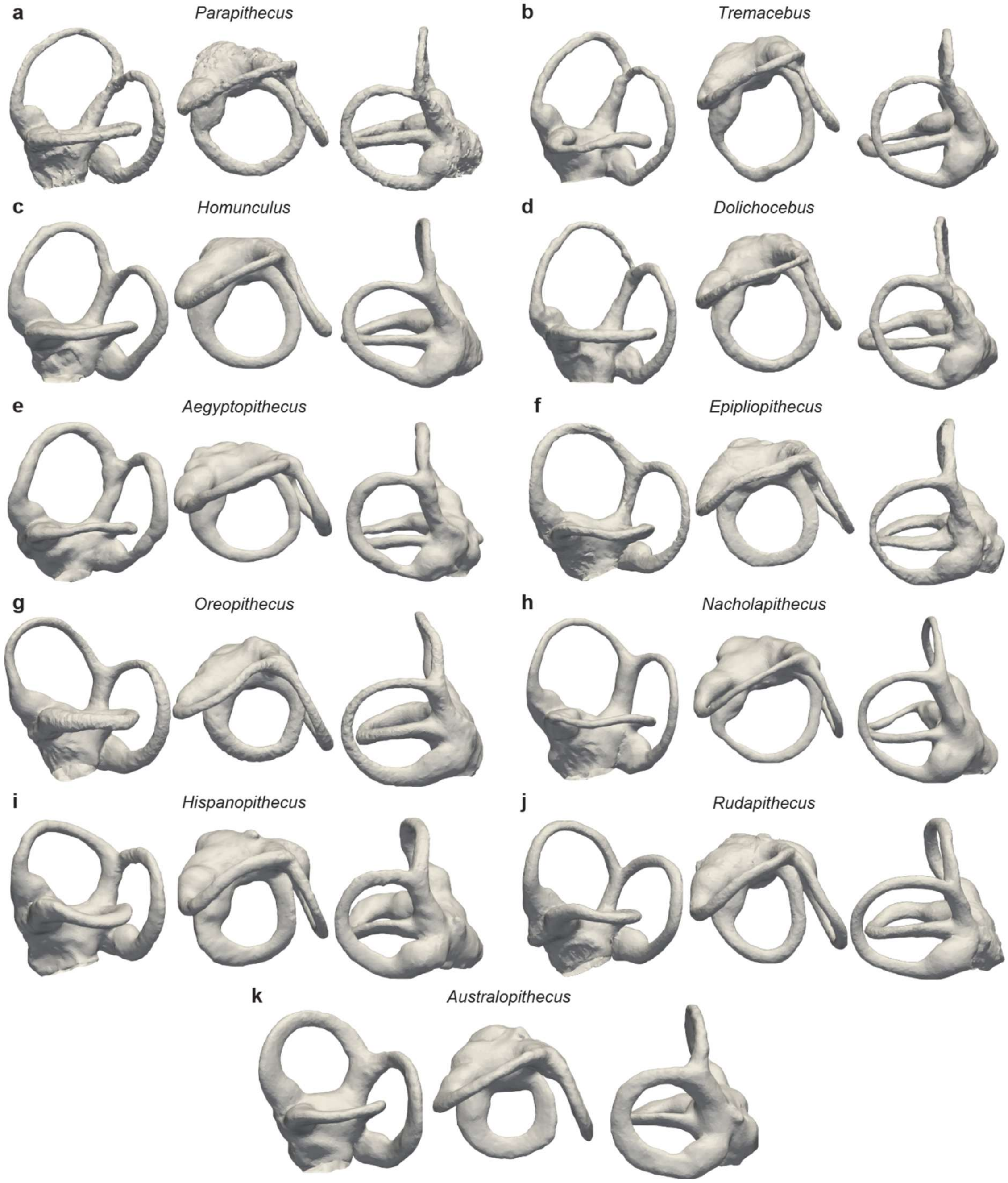

Supplementary Figure S3. Rendering of the semicircular canal and vestibule 3D models of selected extinct anthropoid primates discussed in the present study, from left to right in posterolateral, superior, and posteromedial views (not to scale); a) *Parapithecus grangeri* (DPC 18651); b) *Tremacebus harringtoni* (FMI-619); c) *Homunculus patagonicus* (MPM-PV 3501); d) *Dolichocebus gaimanensis* (MACN

14128); e) *Aegyptopithecus zeuxis* (CGM 85785); f) *Epipliopithecus vindobonensis* (NHMW 1970/1397/0003); g) *Oreopithecus bambolii* (Bac 183); h) *Nacholapithecus kerioi* (KNM-BG 42744); i) *Hispanopithecus laietanus* (IPS18000); j) *Rudapithecus hungaricus* (RUD200); k) *Australopithecus africanus* (StW 578).

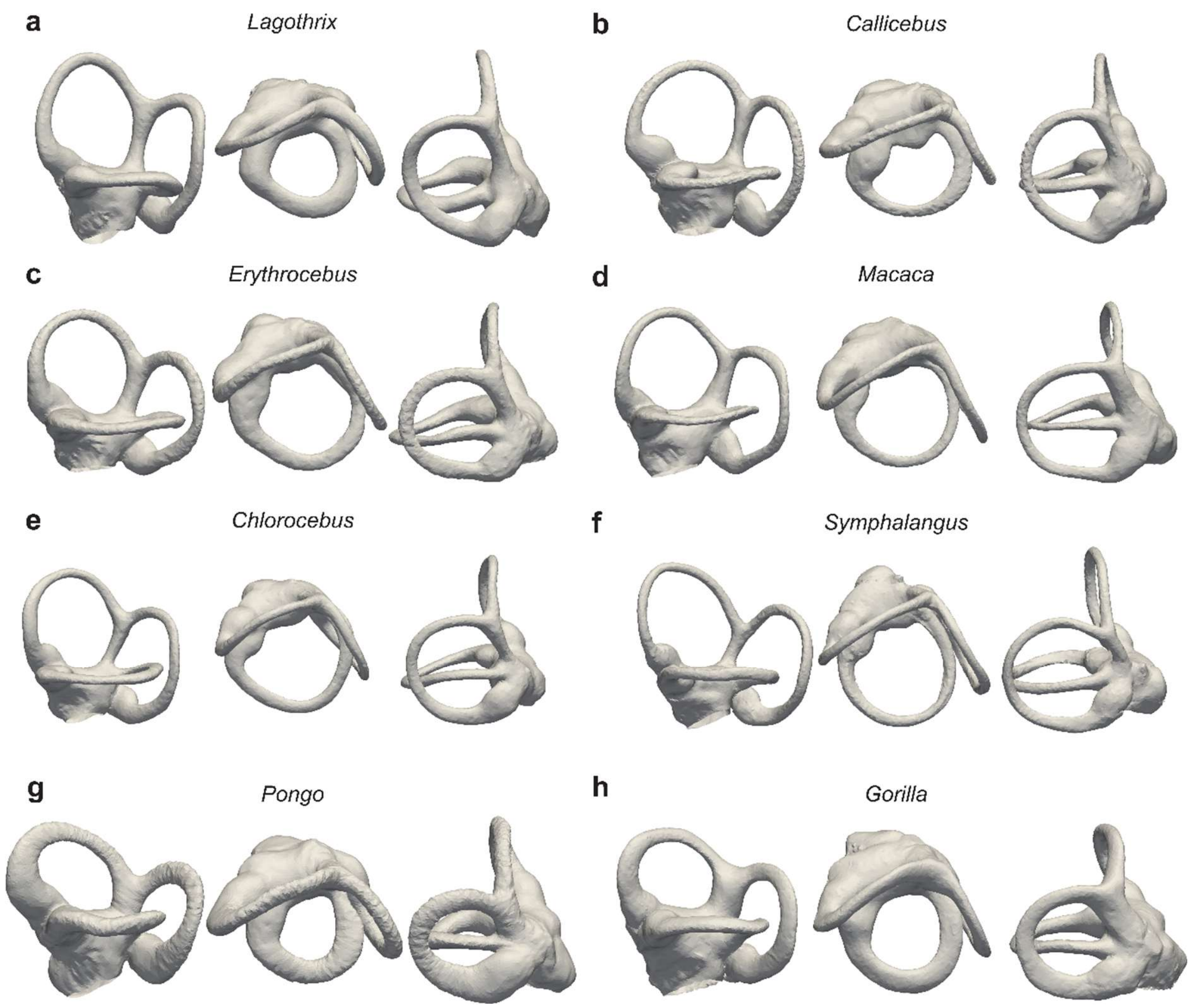

Supplementary Figure S4. Rendering of the semicircular canal and vestibule 3D models of selected extant anthropoid primates discussed in the present study, from left to right in posterolateral, superior, and posteromedial views (not to scale); a) *Lagothrix lagothricia* (USNM 461930); b) *Callicebus moloch* (MCZ 26922); c) *Erythrocebus patas* (MCZ 47015); d) *Macaca fascicularis* (MCZ 12758); e) *Chrolocebus pygerythrus* (SIU 4794); f) *Symphalangus syndactylus* (ESFR 50001912); g) *Pongo pygmaeus* (ESR F50001801); h) *Gorilla gorilla* (ESFR 50001994).

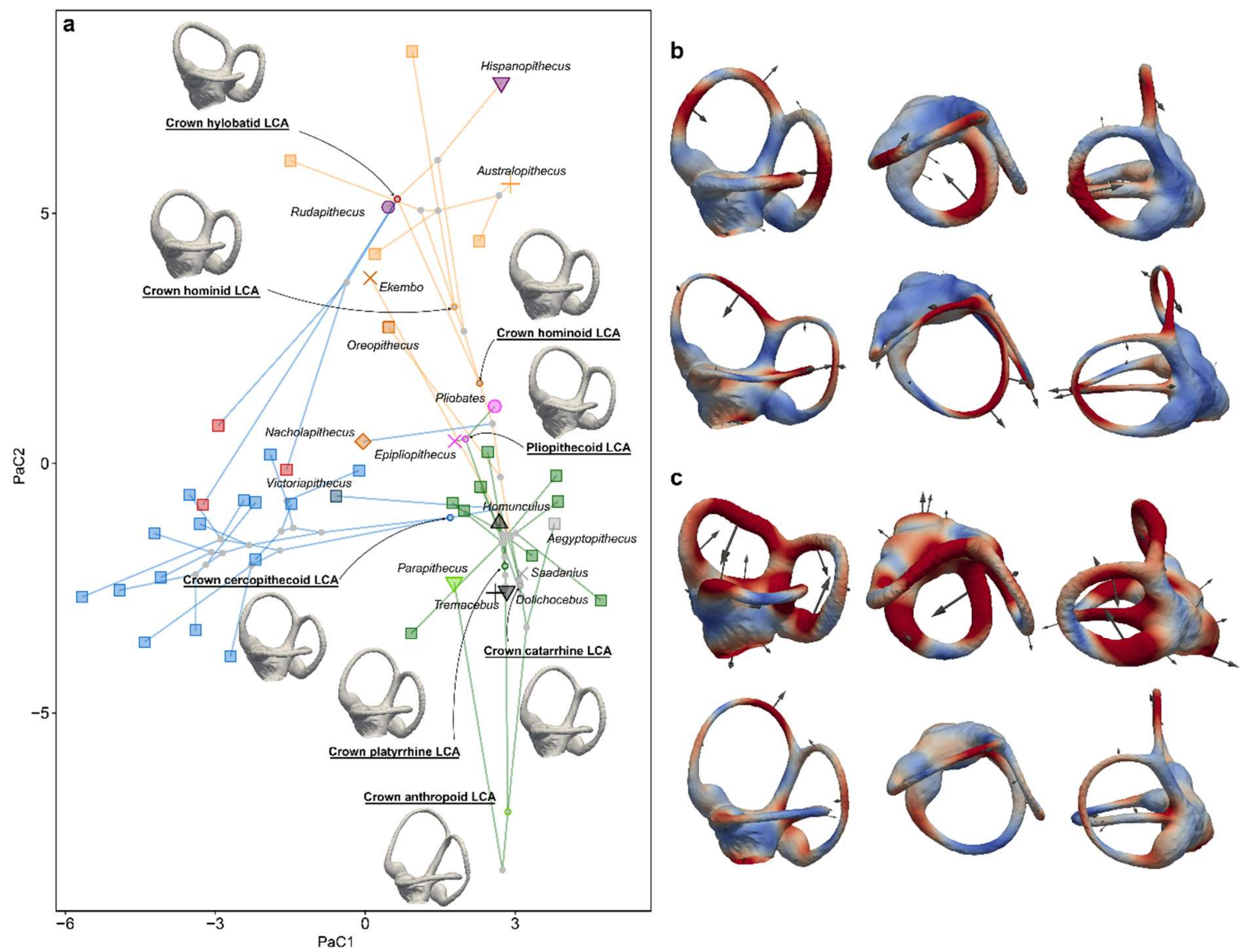

Supplementary Figure S5. Phylomorphospace based on the results of the phylogenetically aligned component analysis by plotting the phylogenetic tree including *Saadanius* as a stem cercopithecoid (Supplementary Figure S6b; see the phylomorphospace plot with the alternative phylogenetic hypothesis in Fig. 5) onto the morphospace and estimating the internal nodes with maximum likelihood based on an Ornstein-Uhlenbeck evolutionary model of evolution with three adaptive optima. a) Bivariate plot of PaC2 vs. PaC1. The 3D models (in posterolateral view) of the estimated LCAs are shown next to the corresponding nodes. To the right, renderings of 3D surfaces, in posterolateral (left), superior (middle), and posteromedial (right) views, corresponding to the maximum (top) and minimum (bottom) conformations for: b) PaC1; c) PaC2. Cumulative deformations from the sample mean are rendered as pseudocolors, with warmer and colder colors corresponding to maximum and

minimum displacement, respectively. Symbols are colored based on taxonomy as follows: stem anthropoids = light green; stem platyrrhines = black; stem catarrhines = gray; stem cercopithecoids = navy blue; crown cercopithecoids = dark turquoise blue; stem hominoids = dark orange; stem hominids = purple; crown platyrrhines = dark green; crown hylobatids = red; crown hominids = light orange. Branches are colored based on the assigned adaptive regime: green = base anthropoid; blue = cercopithecoid; orange = hominid.

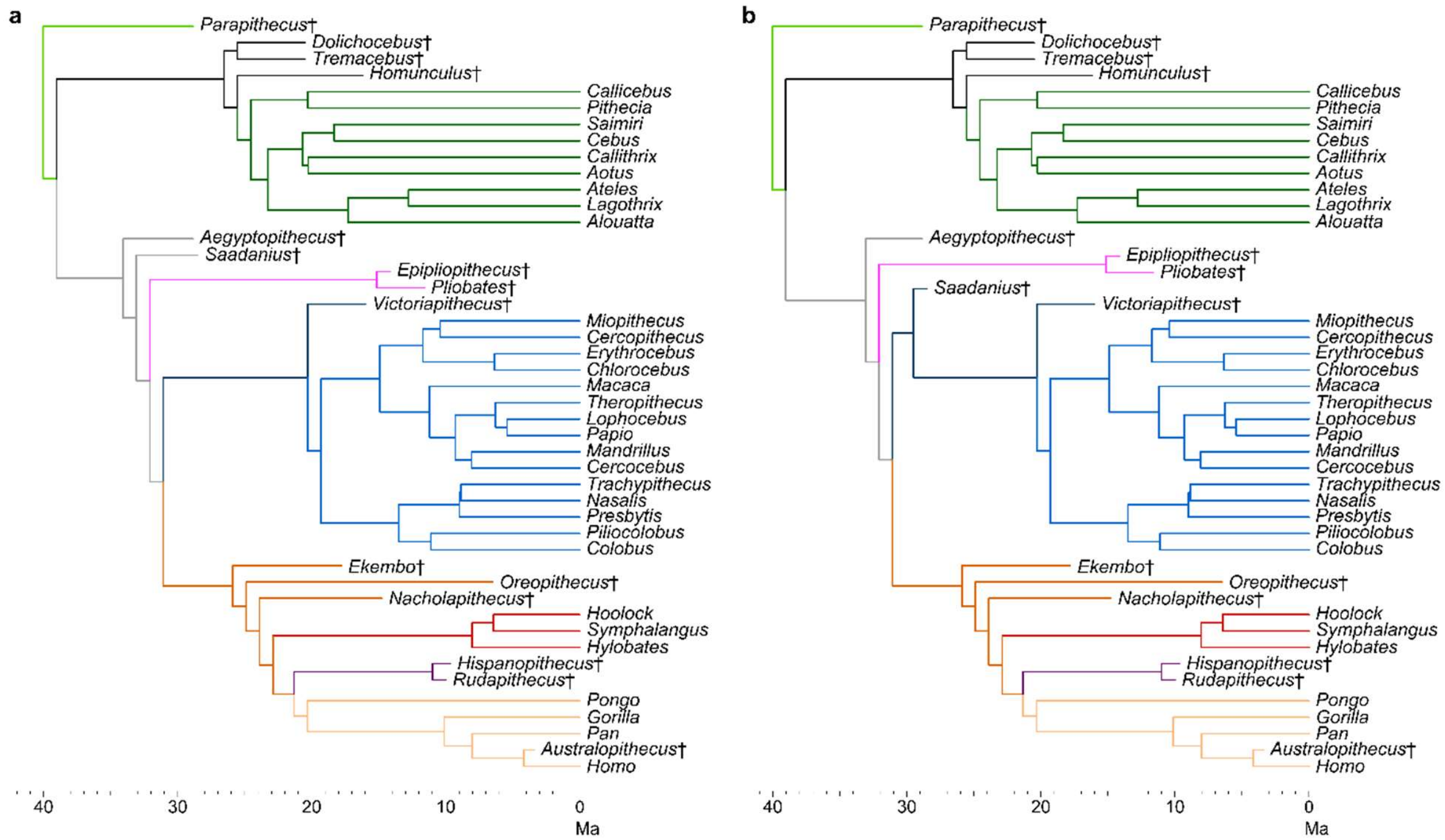

Supplementary Figure S6. Phylogenetic trees based on a molecular phylogeny of extant anthropoid primates[20,22]. Extinct taxa (†) are placed following morphology-based cladistic analyses of extant and extinct anthropoids[7,21,35–37]. Two phylogenetic hypotheses have been considered: a) *Saadanius* as a stem catarrhine less derived than pliopithecoids[21]; b) *Saadanius* as a stem cercopithecoid less derived than *Victoriapithecus*[7]. Tip ages for extinct primates are found in Supplementary Table S6. Branches are color-coded based on taxonomy as follows: stem anthropoids = light green; stem platyrrhines = black; stem catarrhines = gray; stem cercopithecoids = navy blue; crown cercopithecoids = dark turquoise blue; stem hominoids = dark orange; stem hominids = purple; crown platyrrhines = dark green; crown hylobatids = red; crown hominids = light orange.

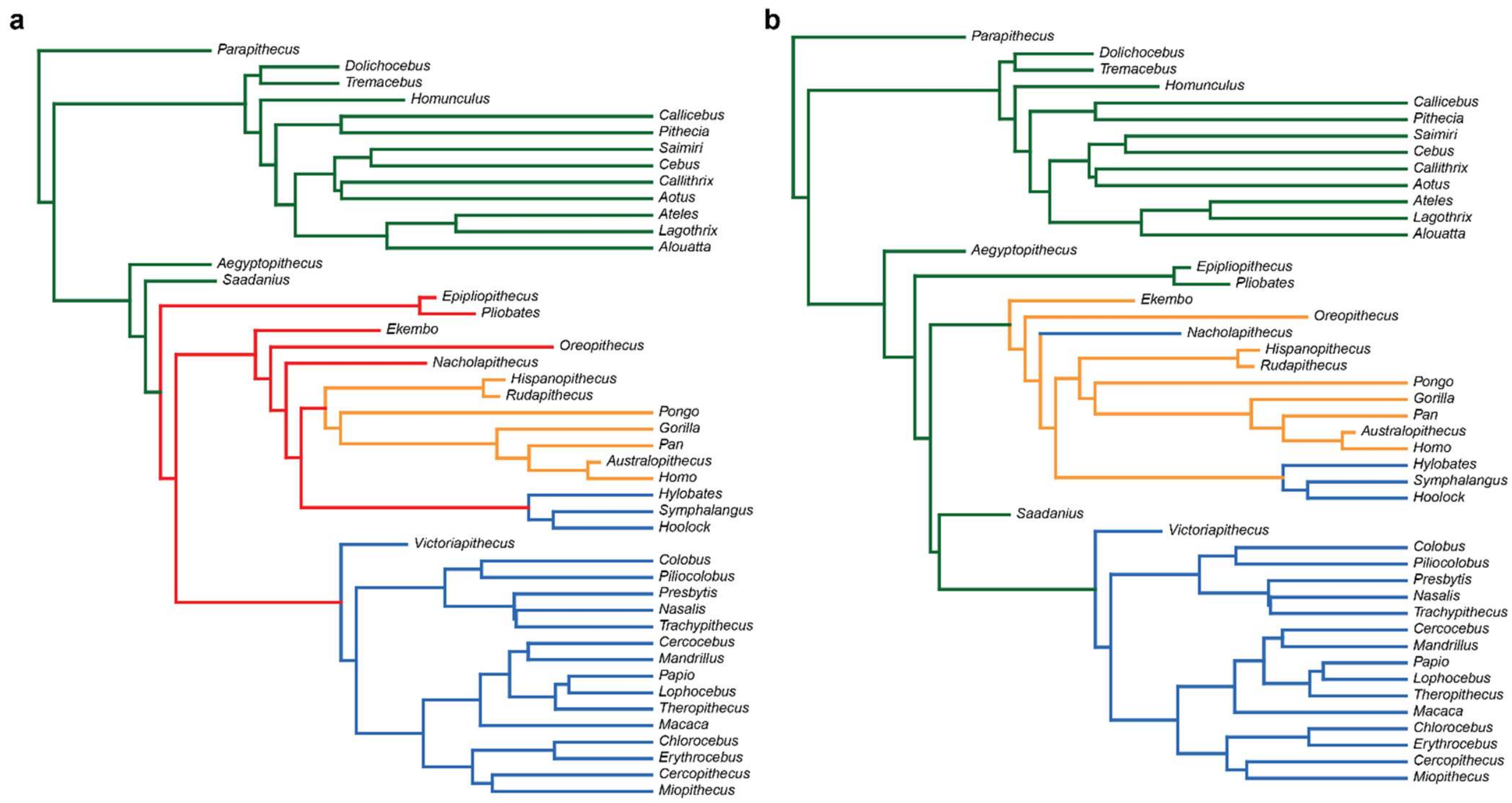

Supplementary Figure S7. Best fit adaptive landscapes for *Saadanius* considered as a stem catarrhine less derived than pliopithecoids but more than propliopithecoids[21] (a; four adaptive optima) and as a stem cercopithecoid less derived than *Victoriapithecus*[7] (b; three adaptive optima). Alternative trees tested in the evolutionary model analysis are shown in Supplementary Data S4. Branches are colored based on the assigned adaptive regime: green = base anthropoid; blue = cercopithecoid; orange = hominid.

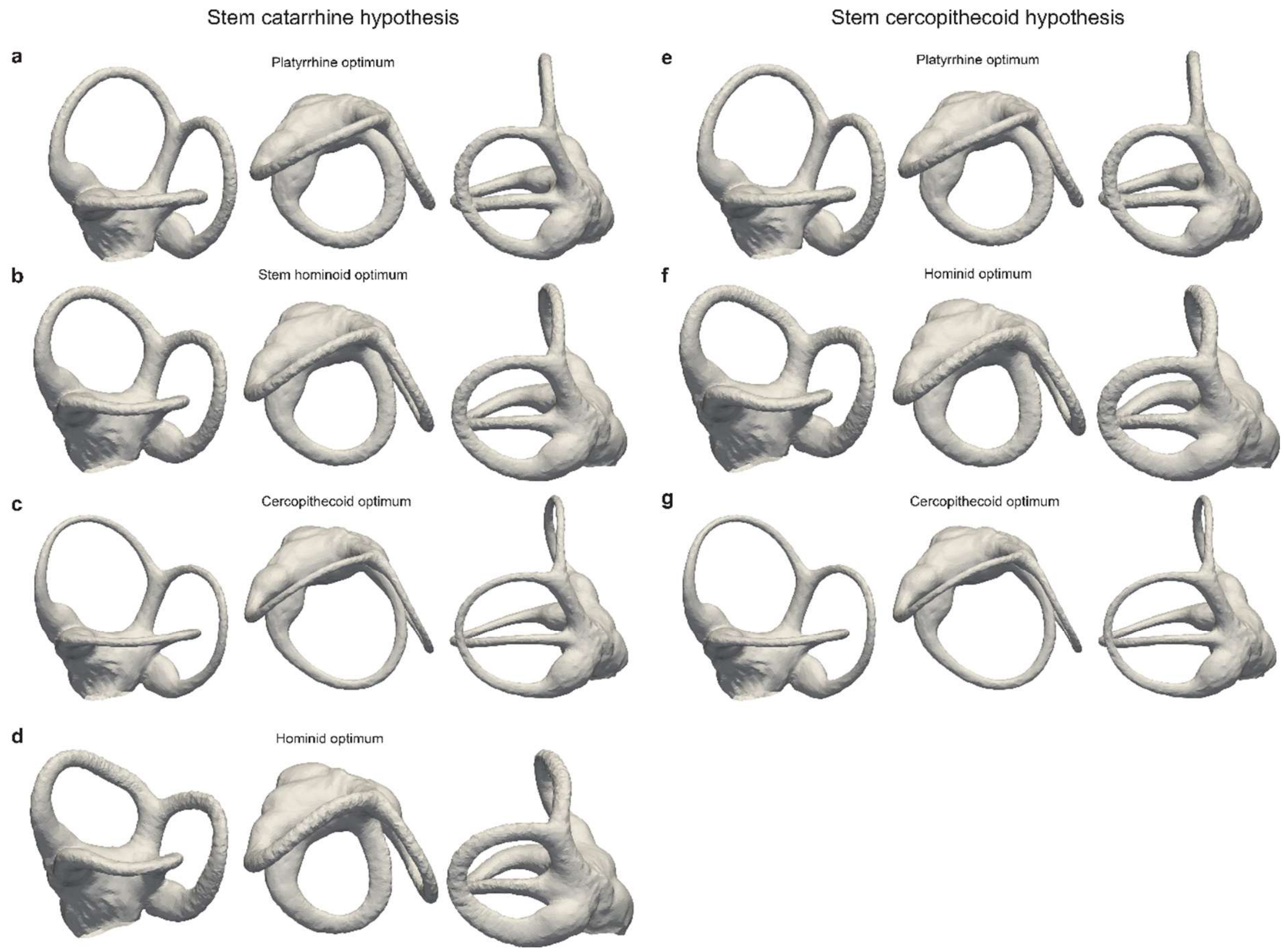

Supplementary Figure S8. Inferred shape for the adaptive optima based on the multi-regime evolutionary landscapes and phylogenetic hypotheses shown in Supplementary Fig. S7. (a–d) adaptive optima reconstructed based on a stem catarrhine hypothesis for *Saadanius* and four adaptive regimes; (e–f) adaptive optima reconstructed based on a stem cercopithecoid hypothesis for *Saadanius* and three adaptive regimes.

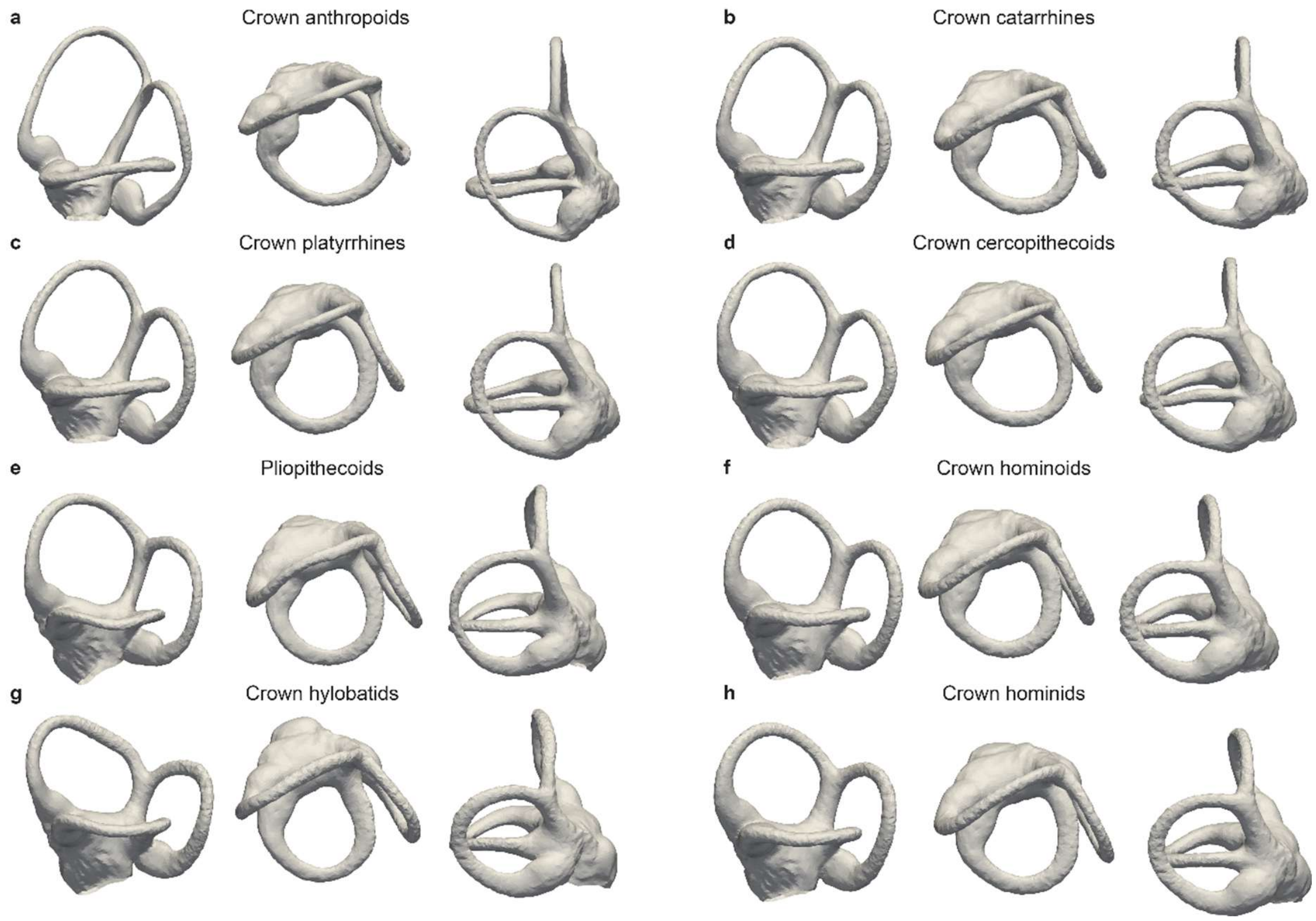

Supplementary Figure S9. Shape of the last common ancestors (LCAs) of major anthropoid clade estimated using maximum likelihood (assuming an Ornstein-Uhlenbeck evolutionary model of evolution with three adaptive optima) based on the results of the PACA performed using the phylogenetic tree including *Saadanius* as a stem cercopithecoid[8] and shown in Supplementary Figures S6 and S7a. The estimated scores of the LCAs are then rotated back into the shape space to visualize the corresponding shape. The 3D models (not to scale) are shown in posterolateral (left), superior (middle), and posteromedial (right) view.

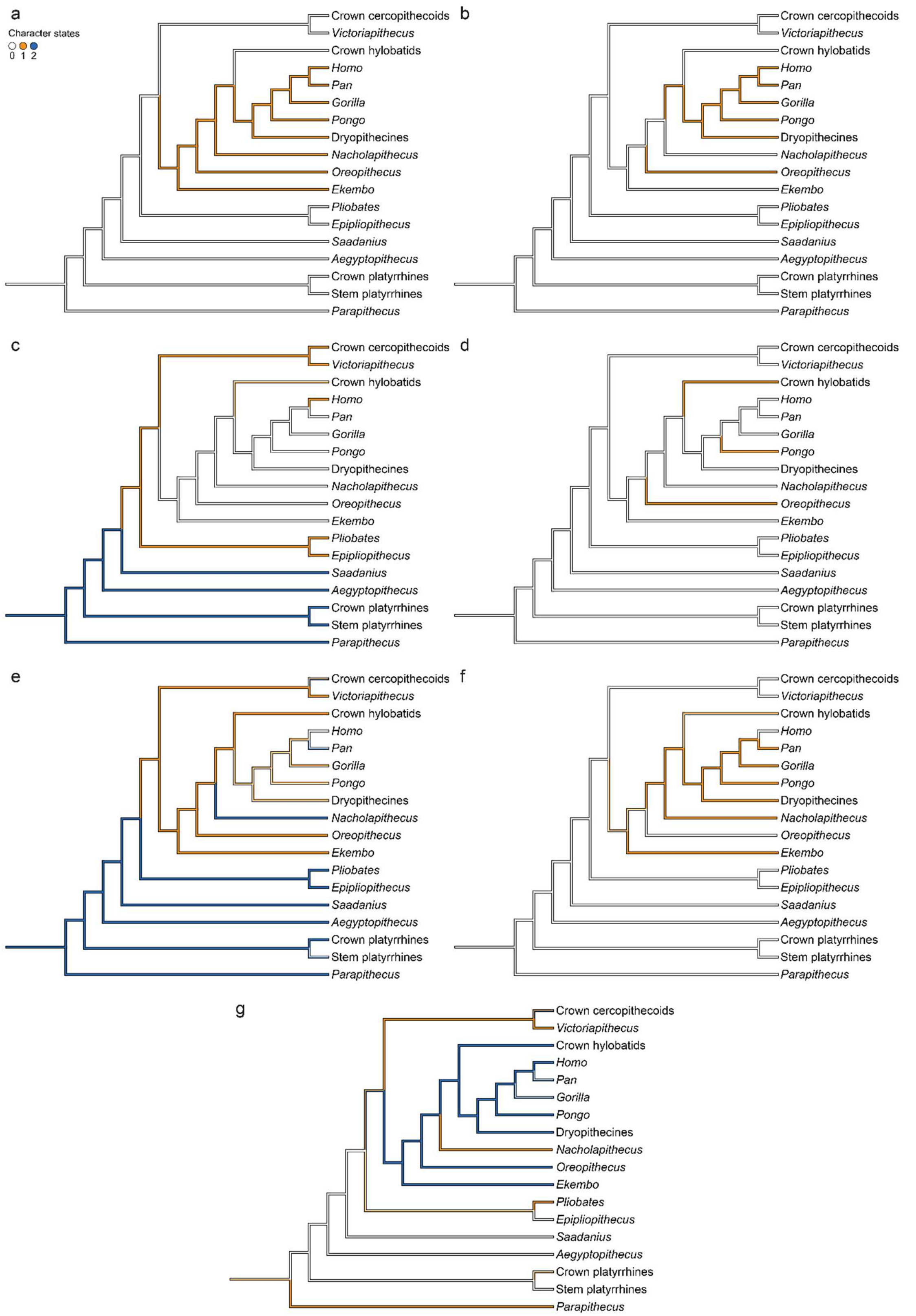

Supplementary Figure S10. Mapping of character states defined for the semicircular

canal and vestibule based on refs. 8, 21 and reported in Supplementary Table S7. a-f) Character #1–#7 coding mapped onto a simplified cladogram of extant anthropoids and extinct taxa based on the stem catarrhine hypothesis for *Saadanius*[21] as shown in Supplementary Figure S6a.

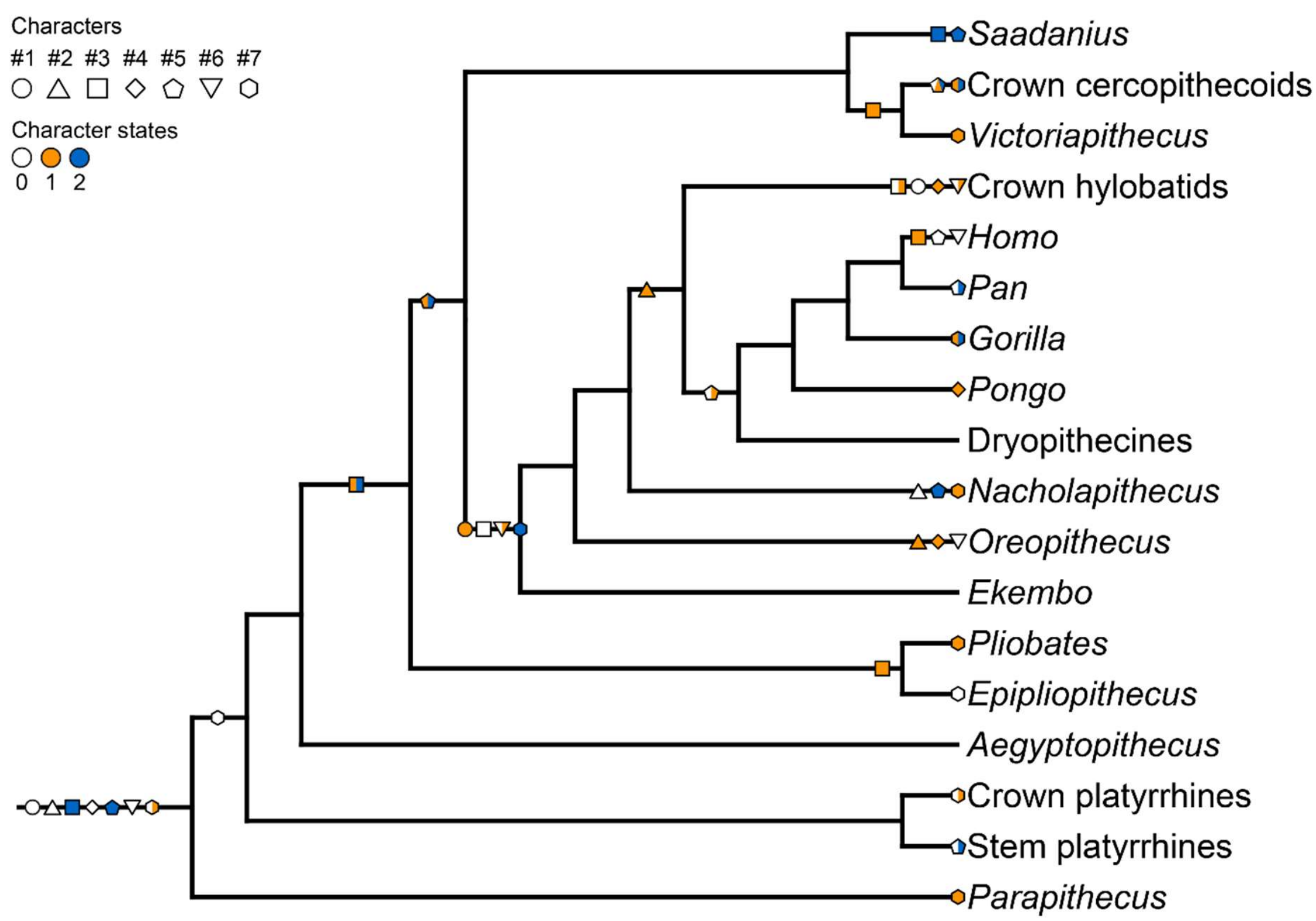

Supplementary Figure S11. Simplified cladogram of extant anthropoids and selected extinct taxa mapping the morphological characters defined for the semicircular canal and vestibule based on refs. 8, 21 and reported in Supplementary Fig. S12 and Supplementary Table S3. Cladogram topology is based on the stem cercopithecoid phylogenetic hypothesis for *Saadanius*[21] (see Fig. 6 for the results based on the alternative phylogenetic hypothesis). Character definitions are as follows: #1 Size of the vestibule relative to the SCs: 0 = small; 1 = large; #2 Robusticity of the SCs: 0 = slender; 1 = stout; #3 Shape of the anterior SC: 0 = vertically compressed; 1 = rounded; 2 = elongated superiorly; #4 Shape of the anterior portion of the anterior SC: 0 = non-projecting anterosuperiorly; 1 = anterosuperiorly projecting; #5 Shape of the posterior SC: 0 = vertically compressed; 1 = rounded; 2 = elongated superiorly; #6 Shape of the lateral SC ampullary portion: 0 = flat or only slightly bent superiorly; 1 = markedly bent

superiorly; #7 Length of the CC: 0 = long; 1 = intermediate; 2 = short. Shape and colors correspond to characters and coding.

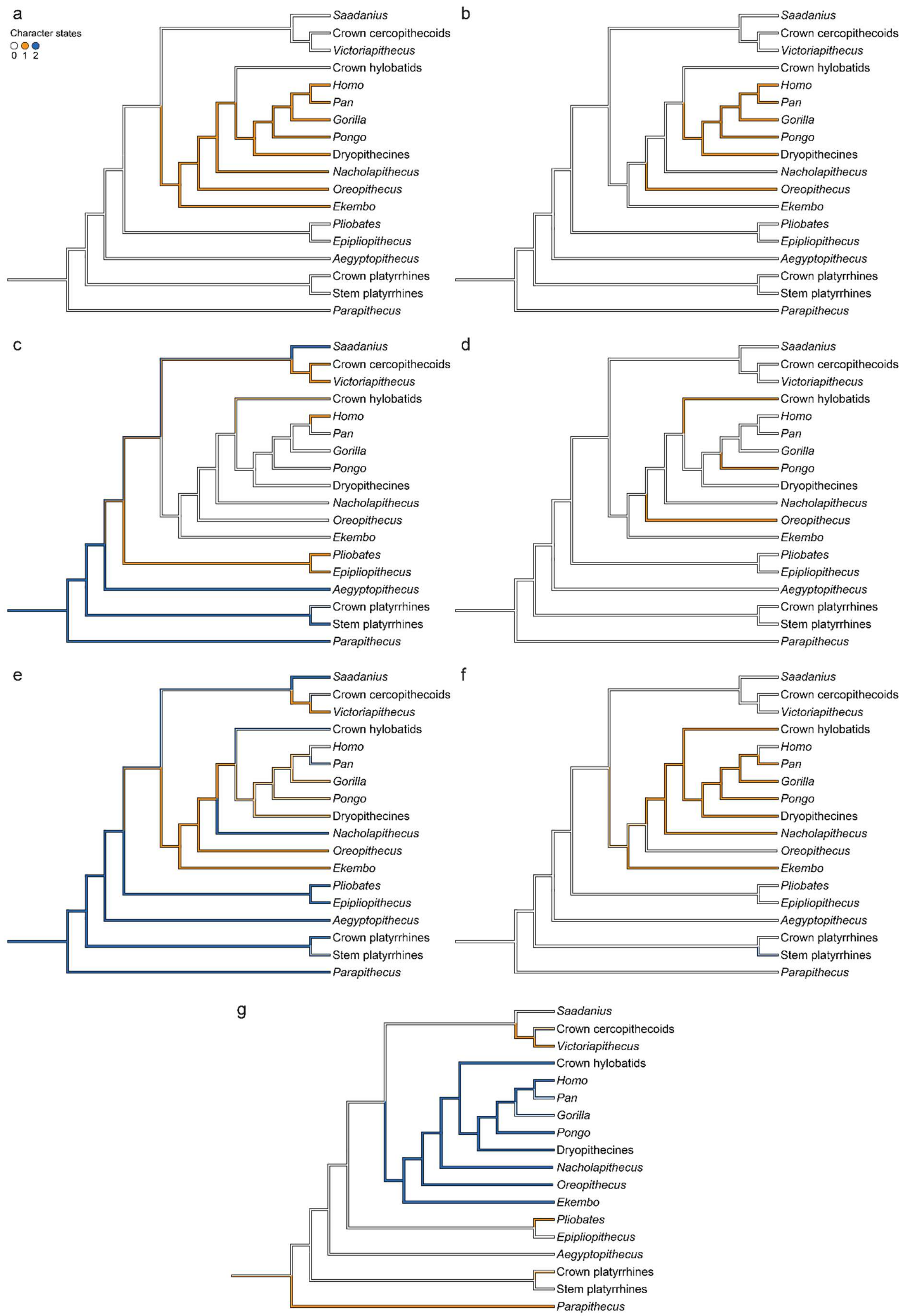

Supplementary Figure S12. Mapping of character states defined for the semicircular

canal and vestibule based on refs. 8, 21 and reported in Supplementary Table S3. a-f) Character #1–#7 coding mapped onto a simplified cladogram of extant anthropoids and extinct taxa based on the stem cercopithecoid hypothesis for *Saadanius*[7,28] as shown in Supplementary Figure S6b.

Supplementary Table S1. Posterior and typicality probabilities of belonging to platyrrhines, cercopithecoids, hylobatids, and hominids computed on canonical variate analysis scores of selected fossil specimens and based on the distribution of the whole anthropoid sample.

Species (catalog No.)	Posterior probabilities				Typicality probabilities[a]			
	Platyrrhini	Cercopithecoidea	Hylobatidae	Hominidae	Platyrrhini	Cercopithecoidea	Hylobatidae	Hominidae
Parapithecus grangeri (DPC 18651)	**98.07%**	1.93%	0.00%	0.00%	**0.588**	0.003	< 0.001	< 0.001
Aegyptopithecus zeuxis (CGM 85785)	**100.00%**	0.00%	0.00%	0.00%	**0.115**	< 0.001	< 0.001	< 0.001
Aegyptopithecus zeuxis (DPC12081)	**100.00%**	0.00%	0.00%	0.00%	**0.209**	< 0.001	< 0.001	< 0.001
Saadanius hijazensis (SGS-UM 2009-002)	**100.00%**	0.00%	0.00%	0.00%	**0.464**	< 0.001	< 0.001	< 0.001

Pliobates cataloniae (IPS58443)	**98.38%**	0.10%	0.00%	1.52%	**0.125**	< 0.001	< 0.001	0.002
Epipliopithecus vindobonensis (NHMW 1970/1397/0003)	**88.73%**	1.66%	7.51%	2.10%	0.044	0.001	0.005	0.001
Epipliopithecus vindobonensis (NMB OE 303a)	**96.06%**	3.55%	0.00%	0.39%	**0.330**	0.018	< 0.001	0.002
Epipliopithecus vindobonensis (NMB OE 303b)	**93.33%**	6.42%	0.00%	0.43%	**0.230**	0.021	< 0.001	0.002
Victoriapithecus macinnesi (KNM-MB-29100)	16.11%	**83.87%**	0.02%	0.00%	0.054	**0.228**	< 0.001	< 0.001
Ekembo heseloni (KNM-RU-2036al)	0.00%	0.00%	0.00%	**100.00%**	< 0.001	0.001	< 0.001	**0.268**

Oreopithecus bambolii (BAC 183)	3.38%	**96.10%**	0.00%	0.52%	0.02	0.372	< 0.001	0.004
Nacholapithecus kerioi (KNM-BG-42744)	13.80%	**74.60%**	0.00%	11.60%	< 0.001	0.002	< 0.001	< 0.001

Abbreviations: IPS = acronym of the collections of the Institut Català de Paleontologia Miquel Crusafont (formerly Institut de Paleontologia de Sabadell), Sabadell, Spain; DPC = Duke Primate Center, Durham, NC, USA; CGM = Egyptian Geological Museum, Cairo, Egypt; SGS = Saudi Geological Survey in Jeddah, Saudi Arabia; NHMW = Naturhistorisches Museum of Wien, Austria; NMB = Naturhistorisches Museum of Basel, Switzerland; Bac = NMB accession number for *Oreopithecus bambolii* specimens; KNM = National Museums of Kenya, Nairobi, Kenya.

[a] The highest probability for each specimen is bolded. Note that typicality probabilities denote the likelihood of an individual representing an outlier with respect to a group defined a priori based on Mahalanobis distances from the group centroid. They estimate the probability of belonging to a particular group defined a priori given a particular score, based on the distribution of specimens from that group relative to the group's centroid, so that when $p < 0.5$ the null hypothesis of group membership is rejected.

Supplementary Table S2. Results of the evolutionary model analysis computed on the phylogenetically aligned components of the anthropoid semicircular canal and vestibule shape variation. Small sample size corrected Akaike information criterion (AICc) and Akaike weights (Aw) are reported for each evolutionary model, based on both the stem catarrhine[21] and stem cercopithecoid[7] hypothesis for *Saadanius*. The smallest AICc denotes the model with the best support, whereas Aw represents the probability for each of the tested models. Evolutionary model hypotheses are summarized as colored trees in Supplementary Fig. S7 and Supplementary Data S4.

Stem catarrhine hypothesis		
Evolutionary model	AICc	Aw
OUM3c	322.828	47.7%
OUM4d	323.307	37.5%
OUM3a	327.152	5.5%
OUM4a	327.641	4.3%
OUM3d	328.120	3.4%
OUM4b	330.041	1.3%
OUM3b	333.785	0.2%
OUM4c	334.297	0.1%
OU1	375.800	0%
EB	380.638	0%
BM	380.926	0%
Stem cercopithecoid hypothesis		

OUM4a	321.440	38.2%
OUM3c	322.411	23.4%
OUM4d	322.869	18.7%
OUM4b	323.221	15.7%
OUM3a	326.803	2.6%
OUM3b	330.054	0.5%
OUM4c	330.592	0.4%
OUM3d	332.145	0.2%
OUM3d	332.145	0.2%
OUM4e	332.717	0.1%
OU1	372.442	0.0%
BM	377.124	0.0%
EB	377.185	0.0%

Supplementary Table S3. Character states estimated for selected ancestral nodes based on the two phylogenetic hypotheses for *Saadanius* reported in Supplementary Fig. S6.

	Stem catarrhine hypothesis							Stem cercopithecoid hypothesis						
LCAs	#1	#2	#3	#4	#5	#6	#7	#1	#2	#3	#4	#5	#6	#7
Crown anthropoid LCA	0	0	2	0	2	0	0	0	0	2	0	2	0	0
Crown platyrrhine LCA	0	0	2	0	2	0	0	0	0	2	0	2	0	0
Crown catarrhine LCA	0	0	1	0	1	0	1	0	0	**2**	0	**2**	0	**0**
Crown cercopithecoid LCA	0	0	1	0	1	0	1	0	0	1	0	1	0	1
Crown hominoid LCA	**1**	0	0	0	1	1	2	**1**	0	0	0	1	1	2
Crown hylobatid LCA	0	0	0	1	1	1	2	0	0	0	1	1	1	2
Crown hominid LCA	1	1	0	0	0	1	2	1	1	0	0	0	1	2

[a] Character states of LCAs that changed relative to previous codings[8,21] are marked in bold

Supplementary Table S4. Extant anthropoids specimens included in the study as comparative sample.

Species	Catalog No.	Scan source/facility	Voxel size (mm)	DOI/ark	References
Alouatta palliata	DU-EA-LP02	MorphoSource	0.06	10.17602/M2/M6439	5, 8, 11
Alouatta palliata	DU-EA-LP04	MorphoSource	0.05	10.17602/M2/M3748	5, 8, 11
Alouatta palliata	DU-EA-LP06	MorphoSource	0.07	10.17602/M2/M5635	5, 8, 11
Alouatta palliata	DU-EA-LP10	MorphoSource	0.05	10.17602/M2/M3785	5, 8, 11
Alouatta palliata	DU-EA-LP12	MorphoSource	0.06	10.17602/M2/M5628	5, 8, 11
Aotus trivirgatus	MCZ-19801	MorphoSource	0.04	10.17602/M2/M2870	8
Aotus trivirgatus	MCZ-19802	MorphoSource	0.04	10.17602/M2/M2871	8
Aotus trivirgatus	MCZ-19805	MorphoSource	0.04	10.17602/M2/M2872	8
Aotus trivirgatus	MCZ-27214	MorphoSource	0.04	10.17602/M2/M2874	8
Aotus trivirgatus	MCZ-30562	MorphoSource	0.04	10.17602/M2/M2875	8
Ateles geoffroyi	MCZ-10138	MorphoSource	0.06	10.17602/M2/M2917	5, 8, 11
Ateles geoffroyi	MCZ-29626	MorphoSource	0.06	10.17602/M2/M2918	5, 8, 11
Ateles geoffroyi	MCZ-29628	MorphoSource	0.06	10.17602/M2/M2919	5, 8, 11
Ateles geoffroyi	MCZ-29658	MorphoSource	0.06	10.17602/M2/M2920	5, 8, 11
Ateles geoffroyi	MCZ-BOM-5351	MorphoSource	0.06	10.17602/M2/M2889	5, 8, 11
Callithrix argentata	MCZ-30579	MorphoSource	0.04	10.17602/M2/M5192	8
Callithrix argentata	MCZ-30580	MorphoSource	0.04	10.17602/M2/M5193	8
Callithrix argentata	MCZ-30582	MorphoSource	0.04	10.17602/M2/M5194	8
Callithrix argentata	MCZ-30583	MorphoSource	0.04	10.17602/M2/M5195	8
Callithrix argentata	MCZ-32164	MorphoSource	0.04	10.17602/M2/M5196	8
Callicebus moloch	MCZ-20186	MorphoSource	0.05	10.17602/M2/M5158	8
Callicebus moloch	MCZ-20188	MorphoSource	0.05	10.17602/M2/M5159	8
Callicebus moloch	MCZ-26922	MorphoSource	0.05	10.17602/M2/M5160	8
Callicebus moloch	MCZ-30559	MorphoSource	0.05	10.17602/M2/M5161	8
Callicebus moloch	MCZ-30564	MorphoSource	0.05	10.17602/M2/M5167	8

Cebus apella	MCZ-49635	MorphoSource	0.06	10.17602/M2/M5208	5, 8, 11
Cebus apella	MCZ-27891	MorphoSource	0.06	10.17602/M2/M5213	5, 8, 11
Cebus apella	MCZ-31063	MorphoSource	0.07	10.17602/M2/M5218	5, 8, 11
Cebus apella	MCZ-37833	MorphoSource	0.06	10.17602/M2/M5219	5, 8, 11
Cebus apella	MCZ-41090	MorphoSource	0.05	10.17602/M2/M5220	5, 8, 11
Lagothrix lagotricha	DU-BAA-X	MorphoSource	0.06	87602/m4/M1867	8
Lagothrix lagotricha	USNM-269839	MorphoSource	0.06	10.17602/M2/M10193	8
Lagothrix lagotricha	USNM-194342	MorphoSource	0.06	10.17602/M2/M9717	8
Lagothrix lagotricha	USNM-461930	MorphoSource	0.06	10.17602/M2/M10187	8
Lagothrix lagotricha	USNM-461931	MorphoSource	0.06	10.17602/M2/M10188	8
Pithecia monachus	MCZ-20265	MorphoSource	0.05	10.17602/M2/M4710	8
Pithecia monachus	MCZ-20266	MorphoSource	0.05	10.17602/M2/M4709	8
Pithecia monachus	MCZ-27124	MorphoSource	0.05	10.17602/M2/M4708	8
Pithecia monachus	MCZ-30720	MorphoSource	0.05	10.17602/M2/M4705	8
Pithecia monachus	MCZ-BOM-5057	MorphoSource	0.05	10.17602/M2/M4711	8
Saimiri sciureus	AMNH-M-33875	MorphoSource	0.04	87602/m4/M6322	8
Saimiri sciureus	AMNH-M-72068	MorphoSource	0.04	10.17602/M2/M6324	8
Saimiri sciureus	AMNH-M-72074	MorphoSource	0.03	10.17602/M2/M6328	8
Saimiri sciureus	AMNH-M-72078	MorphoSource	0.03	10.17602/M2/M6330	8
Saimiri sciureus	AMNH-M-72079	MorphoSource	0.04	10.17602/M2/M6330	8
Cercocebus galeritus	AMNH-M-52634	MorphoSource	0.06	87602/m4/M13568	5, 8, 11
Cercocebus galeritus	AMNH-M-52635	MorphoSource	0.06	87602/m4/M13608	5, 8, 11
Cercocebus galeritus	AMNH-M-52640	MorphoSource	0.06	87602/m4/M13610	5, 8, 11
Cercocebus galeritus	AMNH-M-52641	MorphoSource	0.07	87602/m4/M13614	5, 8, 11
Cercocebus galeritus	AMNH-M-52645	MorphoSource	0.08	87602/m4/M13620	5, 8, 11
Cercopithecus mitis	MCZ-22734	MorphoSource	0.08	10.17602/M2/M2928	5, 8, 11
Cercopithecus mitis	MCZ-25022	MorphoSource	0.08	10.17602/M2/M2929	5, 8, 11
Cercopithecus mitis	MCZ-26832	MorphoSource	0.08	10.17602/M2/M2930	5, 8, 11
Cercopithecus mitis	MCZ-39389	MorphoSource	0.08	10.17602/M2/M2933	5, 8, 11

Cercopithecus mitis	MCZ-44264	MorphoSource	0.08	10.17602/M2/M2935	5, 8, 11
Chlorocebus pygerythrus	SIU-4792	MorphoSource	0.05	10.17602/M2/M13646	5, 8, 11
Chlorocebus pygerythrus	SIU-4793	MorphoSource	0.05	10.17602/M2/M13652	5, 8, 11
Chlorocebus pygerythrus	SIU-4794	MorphoSource	0.05	10.17602/M2/M13657	5, 8, 11
Chlorocebus pygerythrus	SIU-4795	MorphoSource	0.05	10.17602/M2/M13656	5, 8, 11
Chlorocebus pygerythrus	SIU-4796	MorphoSource	0.05	10.17602/M2/M13658	5, 8, 11
Colobus guereza	AMNH-M-52211	MorphoSource	0.07	87602/m4/M13432	5, 8, 11
Colobus guereza	AMNH-M-52213	MorphoSource	0.07	87602/m4/M12510	5, 8, 11
Colobus guereza	AMNH-M-52225	MorphoSource	0.05	87602/m4/M13434	5, 8, 11
Colobus guereza	AMNH-M-52237	MorphoSource	0.07	87602/m4/M13435	5, 8, 11
Colobus guereza	AMNH-M-52249	MorphoSource	0.07	87602/m4/M12516	5, 8, 11
Erythrocebus patas	MCZ-37280	MorphoSource	0.08	10.17602/M2/M2922	5, 8, 11
Erythrocebus patas	MCZ-47015	MorphoSource	0.08	10.17602/M2/M2923	5, 8, 11
Erythrocebus patas	MCZ-47016	MorphoSource	0.08	10.17602/M2/M2924	5, 8, 11
Erythrocebus patas	MCZ-47017	MorphoSource	0.06	10.17602/M2/M2925	5, 8, 11
Erythrocebus patas	MCZ-47018	MorphoSource	0.08	10.17602/M2/M2926	5, 8, 11
Lophocebus albigena	AMNH-M-52596	MorphoSource	0.07	87602/m4/M13551	5, 8, 11
Lophocebus albigena	AMNH-M-52603	MorphoSource	0.06	87602/m4/M13493	5, 8, 11
Lophocebus albigena	AMNH-M-52606	MorphoSource	0.07	87602/m4/M13545	5, 8, 11
Lophocebus albigena	AMNH-M-52607	MorphoSource	0.09	87602/m4/M13553	5, 8, 11
Lophocebus albigena	AMNH-M-52615	MorphoSource	0.07	87602/m4/M12495	5, 8, 11
Macaca fascicularis	MCZ-12758	MorphoSource	0.06	10.17602/M2/M3028	5, 8, 11
Macaca fascicularis	MCZ-22277	MorphoSource	0.06	10.17602/M2/M3029	5, 8, 11
Macaca fascicularis	MCZ-23812	MorphoSource	0.06	10.17602/M2/M3030	5, 8, 11
Macaca fascicularis	MCZ-23813	MorphoSource	0.06	10.17602/M2/M3031	5, 8, 11
Macaca fascicularis	MCZ-35765	MorphoSource	0.06	10.17602/M2/M3033	5, 8, 11
Mandrillus sphinx	AMNH-A-99.1-2049	MorphoSource	0.08	87602/m4/M26166	5, 8, 11
Mandrillus sphinx	AMNH-A-99.1-2056	MorphoSource	0.08	87602/m4/M26168	5, 8, 11
Mandrillus sphinx	AMNH-M-89362	MorphoSource	0.08	87602/m4/M18098	5, 8, 11

Mandrillus sphinx	AMNH-M-89364	MorphoSource	0.10	87602/m4/M18106	5, 8, 11
Mandrillus sphinx	AMNH-M-89365	MorphoSource	0.06	87602/m4/M18119	5, 8, 11
Miopithecus talapoin	MCZ-19976	MorphoSource	0.05	10.17602/M2/M5095	5, 8, 11
Miopithecus talapoin	MCZ-23196	MorphoSource	0.05	10.17602/M2/M5094	5, 8, 11
Miopithecus talapoin	MCZ-23197	MorphoSource	0.05	10.17602/M2/M5093	5, 8, 11
Miopithecus talapoin	MCZ-34264	MorphoSource	0.05	10.17602/M2/M5092	5, 8, 11
Miopithecus talapoin	MCZ-37278	MorphoSource	0.05	10.17602/M2/M5086	5, 8, 11
Nasalis larvatus	MCZ-37342	MorphoSource	0.07	10.17602/M2/M5079	5, 8, 11
Nasalis larvatus	MCZ-41555	MorphoSource	0.07	10.17602/M2/M5059	5, 8, 11
Nasalis larvatus	MCZ-41559	MorphoSource	0.07	10.17602/M2/M5057	5, 8, 11
Nasalis larvatus	MCZ-41560	MorphoSource	0.07	10.17602/M2/M5056	5, 8, 11
Nasalis larvatus	MCZ-41562	MorphoSource	0.07	10.17602/M2/M5055	5, 8, 11
Papio anubis	AMNH-M-52677	MorphoSource	0.09	87602/m4/M13626	5, 8, 11
Papio anubis	MCZ-17342	MorphoSource	0.11	10.17602/M2/M4885	5, 8, 11
Papio anubis	MCZ-26473	MorphoSource	0.10	10.17602/M2/M4890	5, 8, 11
Papio anubis	MCZ-31619	MorphoSource	0.11	10.17602/M2/M4892	5, 8, 11
Papio anubis	MCZ-BOM-8466	MorphoSource	0.08	10.17602/M2/M4889	5, 8, 11
Piliocolobus badius	MCZ-24080	MorphoSource	0.08	10.17602/M2/M2892	5, 8, 11
Piliocolobus badius	MCZ-24775	MorphoSource	0.09	10.17602/M2/M2893	5, 8, 11
Piliocolobus badius	MCZ-24793	MorphoSource	0.08	10.17602/M2/M2894	5, 8, 11
Piliocolobus badius	MCZ-25627	MorphoSource	0.08	10.17602/M2/M2895	5, 8, 11
Piliocolobus badius	MCZ-25631	MorphoSource	0.09	10.17602/M2/M2896	5, 8, 11
Presbytis rubicunda	MCZ-22276	MorphoSource	0.08	10.17602/M2/M4557	5, 8, 11
Presbytis rubicunda	MCZ-35704	MorphoSource	0.08	10.17602/M2/M4552	5, 8, 11
Presbytis rubicunda	MCZ-35705	MorphoSource	0.08	10.17602/M2/M4551	5, 8, 11
Presbytis rubicunda	MCZ-35706	MorphoSource	0.08	10.17602/M2/M4556	5, 8, 11
Presbytis rubicunda	MCZ-35712	MorphoSource	0.08	10.17602/M2/M4555	5, 8, 11
Theropithecus gelada	AMNH-M-19549	MorphoSource	0.08	87602/m4/M17896	5, 8, 11
Theropithecus gelada	AMNH-M-238034	MorphoSource	0.12	87602/m4/M20353	5, 8, 11

Theropithecus gelada	AMNH-M-60568	MorphoSource	0.08	87602/m4/M13676	5, 8, 11
Theropithecus gelada	AMNH-M-80126	MorphoSource	0.09	87602/m4/M13676	5, 8, 11
Theropithecus gelada	AMNH-M-90309	MorphoSource	0.09	87602/m4/M18336	5, 8, 11
Thrachypithecus cristatus	MCZ-35567	MorphoSource	0.05	10.17602/M2/M4439	5, 8, 11
Thrachypithecus cristatus	MCZ-35584	MorphoSource	0.05	10.17602/M2/M4436	5, 8, 11
Thrachypithecus cristatus	MCZ-35586	MorphoSource	0.05	10.17602/M2/M4435	5, 8, 11
Thrachypithecus cristatus	MCZ-35597	MorphoSource	0.05	10.17602/M2/M4433	5, 8, 11
Thrachypithecus cristatus	MCZ-35603	MorphoSource	0.05	10.17602/M2/M4431	5, 8, 11
Hoolock hoolock	AMNH-201743	MorphoSource	0.06	87602/m4/M49871	5, 8, 11
Hoolock hoolock	AMNH-M-112673	MorphoSource	0.04	10.17602/M2/M38950	5, 8, 11
Hoolock hoolock	AMNH-M-112720	MorphoSource	0.05	87602/m4/M21962	5, 8, 11
Hoolock hoolock	AMNH-M-112983	MorphoSource	0.04	10.17602/M2/M38898	5, 8, 11
Hoolock hoolock	AMNH-M-201742	MorphoSource	0.07	87602/m4/M25947	5, 8, 11
Hoolock hoolock	AMNH-M-83421	MorphoSource	0.04	10.17602/M2/M38899	5, 8, 11
Hoolock hoolock	AMNH-M-83425	MorphoSource	0.08	10.17602/M2/M76648	5, 8, 11
Hylobates lar	AMNH-M-184598	MorphoSource	0.04	10.17602/M2/M38900	5, 8, 11
Hylobates lar	MCZ-41411	MorphoSource	0.07	10.17602/M2/M2959	5, 8, 11
Hylobates lar	MCZ-41412	MorphoSource	0.07	10.17602/M2/M2961	5, 8, 11
Hylobates lar	MCZ-41416	MorphoSource	0.07	10.17602/M2/M2965	5, 8, 11
Hylobates lar	MCZ-41418	MorphoSource	0.07	10.17602/M2/M2967	5, 8, 11
Hylobates lar	MCZ-41421	MorphoSource	0.07	10.17602/M2/M2969	5, 8, 11
Hylobates lar	MCZ-41424	MorphoSource	0.07	10.17602/M2/M3007	5, 8, 11
Hylobates lar	MCZ-41452	MorphoSource	0.07	10.17602/M2/M3011	5, 8, 11
Hylobates lar	MCZ-41455	MorphoSource	0.07	10.17602/M2/M3014	5, 8, 11
Hylobates lar	MCZ-41469	MorphoSource	0.07	10.17602/M2/M3023	5, 8, 11
Symphalangus syndactylus	AMNH-M-102724	MorphoSource	0.06	87602/m4/M26157	5, 8, 11
Symphalangus syndactylus	AMNH-M-106583	MorphoSource	0.08	10.17602/M2/M382914	5, 8, 11
Symphalangus syndactylus	EA-258	MorphoSource	0.11	10.17602/M2/M58633	8, 11
Symphalangus syndactylus	CCEC 50001912	ESRF	0.02	10.15151/ESRF-DC-1634387677	8, 11

Symphalangus syndactylus	MCZ-36031	MorphoSource	0.08	10.17602/M2/M4444	5, 8, 11
Symphalangus syndactylus	MCZ-36032	MorphoSource	0.07	10.17602/M2/M4443	5, 8, 11
Gorilla gorilla	AMNH-A-99-9686	MorphoSource	0.13	87602/m4/M22291	5, 8, 11
Gorilla gorilla	AMNH-A-99-9687	MorphoSource	0.08	87602/m4/M22235	5, 8, 11
Gorilla gorilla	AMNH-M-167338	MorphoSource	0.13	87602/m4/M21964	5, 8, 11
Gorilla gorilla	AMNH-M-54356	MorphoSource	0.11	87602/m4/M22291	5, 8, 11, 23
Gorilla gorilla	CCEC 50001994	ESRF	0.05	10.15151/ESRF-DC-1634319693	8, 11
Gorilla gorilla	MCZ-14750	MorphoSource	0.13	10.17602/M2/M2938	5, 8, 11
Gorilla gorilla	MCZ-17684	MorphoSource	0.13	10.17602/M2/M2943	5, 8, 11
Gorilla gorilla	MCZ-26850	MorphoSource	0.12	10.17602/M2/M2947	5, 8, 11
Gorilla gorilla	MCZ-37264	MorphoSource	0.13	10.17602/M2/M2949	5, 8, 11
Gorilla gorilla	MCZ-37625	MorphoSource	0.12	10.17602/M2/M2950	5, 8, 11
Gorilla gorilla	MCZ-49006	MorphoSource	0.13	10.17602/M2/M2957	5, 8, 11
Homo sapiens	EMBR-121	PSU	0.04	—	5, 8, 11
Homo sapiens	EMBR-179	PSU	0.04	—	5, 8, 11
Homo sapiens	EMBR-212	PSU	0.04	—	5, 8, 11
Homo sapiens	EMBR-281	PSU	0.04	—	5, 8, 11
Homo sapiens	EMBR-473	PSU	0.04	—	5, 8, 11
Homo sapiens	EMBR-479	PSU	0.04	—	5, 8, 11
Homo sapiens	EMBR-583	PSU	0.04	—	5, 8, 11
Homo sapiens	F01	University of Bern	0.06	10.5281/zenodo.3355271	6, 8, 11
Homo sapiens	F03	University of Bern	0.06	10.5281/zenodo.3355271	6, 8, 11
Homo sapiens	F04	University of Bern	0.06	10.5281/zenodo.3355271	6, 8, 11
Homo sapiens	F05	University of Bern	0.06	10.5281/zenodo.3355271	6, 8, 11
Homo sapiens	F06	University of Bern	0.06	10.5281/zenodo.3355271	6, 8, 11
Pan paniscus	AMNH-M-86857	AMNH	0.12	—	5, 8, 11
Pan paniscus	IPS9033	CENIEH	0.07	—	5, 8, 11
Pan paniscus	MCZ-38018	MorphoSource	0.09	10.17602/M2/M4399	5, 8, 11
Pan paniscus	MCZ-38019	MorphoSource	0.09	10.17602/M2/M4398	5, 8, 11

Pan paniscus	MCZ-38020	MorphoSource	0.10	10.17602/M2/M4397	5, 8, 11
Pan sp.	CCEC 50001738	ESRF	0.05	10.15151/ESRF-DC-1634320871	8, 11
Pan sp.	CCEC 50001754	ESRF	0.05	10.15151/ESRF-DC-1634320895	8, 11
Pan sp.	CCEC 50001759	ESRF	0.02	10.15151/ESRF-DC-1634320183	8, 11
Pan sp.	CCEC 50001797	ESRF	0.05	—	8, 11
Pan sp.	CCEC 50001988	ESRF	0.05	10.15151/ESRF-DC-1634320175	8, 11
Pan sp.	CCEC 50002604	ESRF	0.05	10.15151/ESRF-DC-1634320523	8, 11
Pan troglodytes	AMNH-M-167342	MorphoSource	0.09	87602/m4/M21967	5, 8, 11
Pan troglodytes	AMNH-M-167344	MorphoSource	0.11	87602/m4/M23479	5, 8, 11
Pan troglodytes	AMNH-M-51204	MorphoSource	0.08	87602/m4/M21952	5, 8, 11
Pan troglodytes	CCEC 50001793	ESRF	0.05	10.15151/ESRF-DC-1634320191	8, 11
Pan troglodytes	CCEC 50001795	ESRF	0.05	10.15151/ESRF-DC-1634320515	8, 11
Pan troglodytes	CCEC 50001796	ESRF	0.05	10.15151/ESRF-DC-1634320199	8, 11
Pan troglodytes	CCEC 50001799	ESRF	0.05	10.15151/ESRF-DC-1634320855	8, 11
Pan troglodytes	IPS5698	CENIEH	0.08	—	5, 8, 11
Pan troglodytes	MCZ-23167	MorphoSource	0.11	10.17602/M2/M4390	5, 8, 11
Pan troglodytes	MCZ-37260	MorphoSource	0.10	10.17602/M2/M4387	5, 8, 11
Pan troglodytes	MCZ-46414	MorphoSource	0.08	10.17602/M2/M4386	5, 8, 11
Pan troglodytes	MCZ-46415	MorphoSource	0.09	10.17602/M2/M4385	5, 8, 11
Pongo pygmaeus	CCEC 50001801	ESRF	0.05	10.15151/ESRF-DC-1634320879	8, 11
Pongo pygmaeus	IPS10647	CENIEH	0.08	—	5, 8, 11
Pongo pygmaeus	IPS10651	CENIEH	0.08	—	5, 8, 11
Pongo pygmaeus	IPS9031	CENIEH	0.08	—	5, 8, 11
Pongo pygmaeus	IPSSN	CENIEH	0.08	—	5, 8, 11
Pongo pygmaeus	MHNT-ZOO-201108	PSU	0.04	—	5, 8, 11
Pongo pygmaeus	SMF-1576	PSU	0.01	—	5, 8, 11
Pongo pygmaeus	SMF-6782	PSU	0.01	—	5, 8, 11
Pongo sp.	MRAC-5593	PSU	0.01	—	5, 8, 11

Abbreviations of scanning facilities: AMNH, American Museum of Natural History, New York, NY, USA; CENIEH, Centro Nacional de Investigación sobre la Evolución Humana, Burgos, Spain; ESRF, European Synchrotron Radiation Facility, Grenoble, France; PSU, Paul Sabatier University, Toulouse, France.

Supplementary Table S5

List of µCT image stacks of the fossil comparative sample.

Catalog No.	Taxon	DOI/ark	Voxel size (mm)[a]	Reference
DPC 18651	*Parapithecus grangeri*	87602/m4/M39638	0.0353	38
FMI-619	*Tremacebus harringtoni*	10.17602/M2/M9339	0.042 × 0.042 × 0.0466	39
MACN 14128	*Dolichocebus gaimanensis*	10.17602/M2/M9459	0.047	35
MPM-PV 3501	*Homunculus patagonicus*	10.17602/M2/M9341	0.040	39
MPM-PV 3502	*Homunculus patagonicus*	10.17602/M2/M9345	0.039	39
MPM-PV 3503	*Homunculus patagonicus*	10.17602/M2/M9347	0.044	39
CGM 85785	*Aegyptopithecus zeuxis*	10.17602/M2/M35988	0.05600 × 0.05600 × 0.06379	40
DPC 12081	*Aegyptopithecus zeuxis*	87602/m4/M36825	0.035903	40
SGS-UM 2009-002	*Saadanius hijazensis*	10.7302/t0ty-pj64	0.0644 × 0.0644 × 0.069	2,3
NMBOE 303a (individual III)	*Epipliopithecus vindobonensis*	10.17602/M2/M113933	0.022	8
NMBOE 303b (individual III)	*Epipliopithecus vindobonensis*	10.17602/M2/M113935	0.022	8
NHMW 1970/1397/0003 (individual II)	*Epipliopithecus vindobonensis*	10.17602/M2/M113932	0.025	8
KNM-MB 29100	*Victoriapithecus macinnesi*	—	0.043793	—
KNM-RU-2036al[c]	*Ekembo heseloni*	—	0.063865	—

Bac 183[b]	*Oreopithecus bambolii*	10.17602/M2/M166428	0.02905	41
KNM-BG 42744	*Nacholapithecus kerioi*	87602/m4/M166427	0.05	42
IPS18000	*Hispanopithecus laietanus*	10.17602/M2/M126217	0.0295	11
RUD 77R	*Rudapithecus hungaricus*	10.17602/M2/M126214	0.0136	11
RUD 77L	*Rudapithecus hungaricus*	10.17602/M2/M126215	0.0136	11
RUD 200	*Rudapithecus hungaricus*	10.17602/M2/M126216	0.0136	11
StW 573	*Australopithecus africanus*	87602/m4/M68769	0.09	43
StW 578	*Australopithecus africanus*	87602/m4/M68767	0.07	43

Abbreviations: IPS = acronym of the collections of the Institut Català de Paleontologia Miquel Crusafont (formerly Institut de Paleontologia de Sabadell), Sabadell, Spain; DPC = Duke Primate Center, Durham, NC, USA; CGM = Egyptian Geological Museum, Cairo, Egypt; SGS = Saudi Geological Survey in Jeddah, Saudi Arabia; NHMW = Naturhistorisches Museum of Wien, Austria; MPM-PV = Museo Regional Provincial Padre M.J. Molina, Rio Gallegos, Argentina; MACN = Museo Argentino de Ciencias Naturales, Buenos Aires, Argentina; NMB = Naturhistorisches Museum of Basel, Switzerland; Bac = NMB accession number for *Oreopithecus bambolii* specimens; KNM = National Museums of Kenya, Nairobi, Kenya; StW = Sterkfontein, housed at Wits University, Johannesburg, South Africa; RUD = Rudabánya, housed at the Geological Museum of the Mining and Geological Survey of Hungary, Budapest, Hungary; FMI = Facultad de Ciencias Naturales e Instituto Miguel Lillo: Colección de Paleontología de Vertebrados Lillo, Tucumán, Argentina.

[a] Voxel size for isometric μCT image stacks repeated just once.

[b] The individual was incorrectly labeled as Bac 208 in some previous papers [5, 8, 11,41]

[c] Access to the µCT scans was kindly provided by Fred Spoor, Fredrick Kyalo Manthi, and Isaiah Nengo.

Supplementary Table S6. Extinct anthropoids included in the studied and comparative samples.

Taxon	Tip age	References
Parapithecus grangeri	29.85 Ma[a]	44
Aegyptopithecus zeuxis	29.85 Ma[a]	44
Saadanius hijazensis	28.5 Ma[a]	2
Pliobates cataloniae	11.6 Ma	45–46
Epipliopithecus vindobonensis	14.15 Ma[a]	47
Dolichocebus gaimanensis	20.5 Ma[a]	35
Homunculus patagonicus	17.2 Ma[a]	35
Tremacebus harringtoni	20.5 Ma[a]	35
Victoriapithecus macinnesi	15 Ma	48–49
Ekembo heseloni	17.25 Ma[a]	50
Hispanopithecus laietanus	9.6 Ma	51
Rudapithecus hungaricus	10.1 Ma	51
Oreopithecus bambolii	6.75 Ma[a]	52
Nacholapithecus kerioi	14.77 Ma	53–54
Australopithecus africanus	4.02 Ma[b]	55

[a] Tip age based on a range: *Saadanius,* average of 29.0–28.0 Ma; *Epipliopithecus,* average of maximum-minimum age range for MN6 in central Europe, 14.85–13.45 Ma; *Parapithecus* and *Aegyptopithecus*, age range of the fauna of quarries I and M of the Jebel Qatrani Formation of the Fayum depression, 30.2–29.5 Ma; *Dolichocebus* and *Tremacebus*, 21.0–20.0 Ma; *Homunculus*, 17.9–16.5 Ma; *Oreopithecus*, 7.0–6.5 Ma; *Ekembo*, average of Mfangano Island sites and the Hiwegi, Kulu, and Kiahera formations at Rusinga Island (18.5–16.0 Ma);

[b] The tip for *Australopithecus* corresponds to the first appearance datum for *Australopithecus africanus* including the Jacovec specimens

Supplementary Table S7

Matrix of character states for the extant genera included in the analysis[a]

Taxon	#1	#2	#3	#4	#5	#6	#7
Alouatta	0	0	B	0	2	0	0
Aotus	0	0	2	0	2	0	A
Ateles	0	0	2	0	2	0	0
Callicebus	0	0	2	0	2	0	0
Callithrix	0	0	2	0	2	0	0
Cebus	0	0	B	0	2	0	0
Cercocebus	0	0	1	0	1	0	1
Cercopithecus	0	0	1	0	B	0	1
Chlorocebus	0	0	1	0	1	0	1
Colobus	0	0	1	0	1	0	1
Erythrocebus	0	0	1	0	1	0	1
Gorilla	1	1	0	0	A	1	B
Homo	1	1	1	0	0	0	2
Hoolock	0	0	A	1	1	1	2
Hylobates	0	0	0	1	1	1	2
Lagothrix	0	0	2	0	2	0	0
Lophocebus	0	0	1	0	B	0	1
Macaca	0	0	1	0	1	0	1
Mandrillus	0	0	1	0	1	0	1
Miopithecus	0	0	1	0	1	0	A
Nasalis	0	0	1	0	A	0	2
Pan	1	1	0	0	B	1	B
Papio	0	0	1	0	1	0	1
Piliocolobus	0	0	2	0	2	0	1

Pithecia	0	0	1	0	1	0	B
Pongo.	0	0	1	0	2	0	A
Presbytis	1	1	0	1	A	1	2
Saimiri	0	0	1	0	1	0	2
Symphalangus	0	0	2	0	2	0	0
Theropithecus	0	0	A	1	A	A	2
Trachypithecus	0	0	1	0	0	0	1

[a] Abbreviations for variable characters: A = (0,1); B = (0,2). For coding of the last common ancestors of main anthropoid clades see Table 1.

Supplementary References

1. Alba, D. M., *et al.* Miocene small-bodied ape from Eurasia sheds light on hominoid evolution. *Science* **350**, aab2625 (2015).
2. Zalmout, I. S., *et al.* New Oligocene primate from Saudi Arabia and the divergence of apes and Old World monkeys. *Nature* **466**, 360–364 (2010).
3. Zalmout, I. S., *et al.* CT data of the partial cranium of SGS-UM 2009-002, *Saadanius hijazensis* (holotype). *University of Michigan - Deep Blue Data* (2020).
4. Urciuoli, A., et al. Neutron-based computed microtomography: *Pliobates cataloniae* and *Barberapithecus huerzeleri* as a test-case study. *Am. J. Phys. Anthropol.* **166**, 987–993 (2018).
5. Urciuoli, A., *et al.* The evolution of the vestibular apparatus in apes and humans. *eLife* **9**, e51261 (2020).
6. Wimmer, W., *et al.* Human bony labyrinth dataset: co-registered CT and micro-CT images, surface models and anatomical landmarks. *Data Brief* **27**, 104724 (2019).
7. Nengo, I., *et al.* New infant cranium from the African Miocene sheds light on ape evolution. *Nature* **548**, 169–174 (2017).
8. Urciuoli, A., *et al.* A comparative analysis of the vestibular apparatus in *Epipliopithecus vindobonensis*: phylogenetic implications. *J. Hum. Evol.* **151**, 102930 (2021).
9. Durrleman, S., *et al.* Morphometry of anatomical shape complexes with dense deformations and sparse parameters. *Neuroimage* **101**, 35–49 (2014).
10. Bône, A., Louis, M., Martin, B., Durrleman, S. Deformetrica 4: an open-source software for statistical shape analysis. In: *Shape in Medical Imaging. Shape MI 2018* (eds Reuter, M., *et al.*) 3–13 (Springer, 2018).
11. Urciuoli, A., *et al.* Reassessment of the phylogenetic relationships of the late Miocene apes *Hispanopithecus* and *Rudapithecus* based on vestibular morphology. *Proc. Natl. Acad. Sci. USA* **118**, e2015215118.
12. Beaudet, A., *et al.* The atlas of StW 573 and the late emergence of human-like head mobility and brain metabolism. *Sci. Rep.* **10**, 4285 (2020).
13. Dumoncel, J., *et al.* Are endocasts reliable proxies for brains? A 3D quantitative comparison of the extant human brain and endocast. *J. Anat.* **238**, 480–488 (2021).

14. Fornai, C., *et al.* New insights on hip bone sexual dimorphism in adolescents and adults using deformation-based geometric morphometrics. *J. Anthropol. Sci.* **99**, 117–134 (2021).
15. Zanolli, C., *et al.* A reassessment of the distinctiveness of dryopithecine genera from the Iberian Miocene based on enamel-dentine junction geometric morphometric analyses. *J. Hum. Evol.* **177**, 103326 (2023).
16. RStudio Team. RStudio: Integrated Development for R (2023).
17. R Core Team. R: A Language and Environment for Statistical Computing. R Foundation for Statistical Computing (2023).
18. Dumoncel, J. *RToolsForDeformetrica*. R package version 0.1 (2021).
19. Schlager, S. Chapter 9 - Morpho and Rvcg – Shape analysis in r: r-packages for geometric morphometrics, shape analysis and surface manipulations. In: *Statistical Shape and Deformation Analysis* (eds. Zheng, G., Li, S. & Székely, G.) (Academic Press, 2017).
20. Kuderna, L. F. K., *et al*. A global catalog of whole-genome diversity from 233 primate species. *Science* **380**, 906–913 (2023).
21. Bouchet, F., *et al.* The Miocene primate *Pliobates* is a pliopithecoid. *Nat. Commun.* **15**, 2822 (2024).
22. Shao, Y., *et al*. Phylogenomic analyses provide insights into primate evolution. *Science* **380**, 913–924 (2023).
23. Smith, C. M., *et al.* Divergent otolithic systems in the inner ear of *Paranthropus robustus* and *Australopithecus africanus*. *J. Hum. Evol.* **199**, 103624 (2025).
24. Collyer, M. L., Adams, D. C. Phylogenetically aligned component analysis. *Methods Ecol. Evol.* **12**, 359–372 (2021).
25. Revell, L. J. Size-correction and principal components for interspecific comparative studies. *Evolution* **63**, 3258–3268 (2009).
26. Sidlauskas, B. Continuous and arrested morphological diversification in sister clades of characiform fishes: A phylomorphospace approach. *Evolution* **62**, 3135–3156 (2008).
27. Clavel, J., Morlon, H. Reliable phylogenetic regressions for multivariate comparative data: Illustration with the MANOVA and application to the effect of diet on mandible morphology in phyllostomid bats. *Syst. Biol.* **69**, 927–943 (2020).

28. Gilbert, C. C., *et al.* New Middle Miocene ape (Primates: Hylobatidae) from Ramnagar, India fills major gaps in the hominoid fossil record. *Proc. R. Soc. B* **287**, 20201655 (2020).
29. Revell, L. J. phytools 2.0: an updated R ecosystem for phylogenetic comparative methods (and other things). *PeerJ* **12**, e16505 (2024).
30. Adams, D. C., Collyer, M. L. Multivariate phylogenetic comparative methods: Evaluation, comparisons, and recommendations. *Syst. Biol.* **67**, 14–31 (2018).
31. Adams, D. C., Collyer, M. L. Multivariate phylogenetic comparative methods: Evaluation, comparisons, and recommendations. *Syst. Biol.* **67**, 14–31 (2018).
32. Adams, D. C., Collyer, M. L., Kaliontzopoulou, A., Baken, E. Geomorph: software for geometric morphometric analyses. *R package version 4.0.8* (2024).
33. Plotly Technologies. Collaborative Data Science https://plot.ly (2015).
34. Maddison, W. P., Maddison, D. R. Mesquite: a modular system for evolutionary analysis. *Version 4.01* (2025).
35. Kay, R. F. Biogeography in deep time – What do phylogenetics, geology, and paleoclimate tell us about early platyrrhine evolution? *Mol. Phylogenet. Evol.* **82**, 358–374 (2015).
36. Kay, R. F., *et al.* The anatomy of *Dolichocebus gaimanensis*, a stem platyrrhine monkey from Argentina. *J. Hum. Evol.* **54** 323–382 (2008).
37. Pugh, K. D. Phylogenetic analysis of Middle-Late Miocene apes. *J. Hum. Evol.* **165**, 103140 (2022).
38. Bush, E. C., Simons, E. L., Allman, J. M. High-resolution computed tomography study of the cranium of a fossil anthropoid primate, *Parapithecus grangeri*: New insights into the evolutionary history of primate sensory systems. *Anat. Rec.* **281A**, 1083–1087 (2004).
39. Fulwood, E. L., Boyer, D. M., Kay, R. F. Stem members of Platyrrhini are distinct from catarrhines in at least one derived cranial feature. *J. Hum. Evol.* **100**, 16–24 (2016).
40. Simons, E. L., Seiffert, E. R., Ryan, T. M., Attia, Y. A remarkable female cranium of the early Oligocene anthropoid *Aegyptopithecus zeuxis* (Catarrhini, Propliopithecidae). *Proc. Natl. Acad. Sci. USA* **104**, 8731–8736 (2007).
41. Rook, L., Bondioli, L., Casali, F., Rossi, M., Köhler, M., Moyà-Solà, S., Macchiarelli, R. The bony labyrinth of *Oreopithecus bambolii*. *J. Hum. Evol.* **46**, 347–354 (2004).

42. Morimoto, N., *et al*. Variation of bony labyrinthine morphology in Mio−Plio−Pleistocene and modern anthropoids. *Am. J. Phys. Anthropol.* **173**, 276–292 (2020).

43. Beaudet, A., *et al*. The bony labyrinth of StW 573 (“Little Foot”): Implications for early hominin evolution and paleobiology. *J. Hum. Evol.* **127**, 67–80 (2019).

44. Seiffert, E. R. Revised age estimates for the later Paleogene mammal faunas of Egypt and Oman. *Proc. Natl. Acad. Sci. USA* **103**, 5000–5005 (2006).

45. Alba, D. M., Casanovas-Vilar, I., Garcés, M., Robles, J. M. Ten years in the dump: An updated review of the Miocene primate-bearing localities from Abocador de Can Mata (NE Iberian Peninsula). *J. Hum. Evol.* **102**, 12–20 (2017).

46. Alba, D. M., *et al*. A revised (earliest Vallesian) age for the hominoid-bearing locality of Can Mata 1 based on new magnetostratigraphic and biostratigraphic data from Abocador de Can Mata (Valles-Penedes Basin, NE Iberian Peninsula). *J. Hum. Evol.* **170**, 103237 (2022).

47. van der Meulen, A. J., *et al*. Biostratigraphy or biochronology? Lessons from the Early and Middle Miocene small Mammal Events in Europe. *Geobios* **44**, 309–321 (2011).

48. Benefit, B. R. *Victoriapithecus*: the key to Old World monkey and catarrhine origins. *Evol. Anthropol.* **7**, 155–174 (1999).

49. Benefit, B. R., McCrossin, M. L. Earliest known Old World monkey skull. *Nature* **388**, 368–371 (1997).

50. Van Couvering, J., Delson, E. African land mammal ages. *J. Vert. Paleontol.* **40**, e1803340 (2020).

51. Casanovas-Vilar, I., Alba, D. M., Garcés, M., Robles, J. M., Moyà-Solà, S. Updated chronology for the Miocene hominoid radiation in Western Eurasia. Proc. Natl. Acad. Sci. USA **108**, 5554–5559 (2011).

52. Rook, L., Oms, O., Benvenuti, M., Papini, M. Magnetostratigraphy of the Late Miocene Baccinello–Cinigiano basin (Tuscany, Italy) and the age of *Oreopithecus bambolii* faunal assemblages. *Palaeogeogr. Palaeoclimatol. Palaeoecol*. **305**, 286–294 (2011).

53. Nakatsukasa, M., Kunimatsu, Y. *Nacholapithecus* and its importance for understanding hominoid evolution. *Evol. Anthropol.* **18**, 103–119 (2009).

54. Kunimatsu, Y., Nakatsukasa, M., Shimizu, D., Nakano, Y., Ishida, H. Loss of the subarcuate fossa and the phylogeny of *Nacholapithecus*. *J. Hum. Evol.* **131**, 22–27 (2019).

55. Wood, B., Boyle, E. K. Hominin taxic diversity: Fact or fantasy? *Am. J. Phys. Anthropol.* **159**, S37–S78 (2016).
